# A Novel Dynamic Impact Approach **(**DIA**)** for Functional Analysis of Time-Course Omics Studies: Validation Using the Bovine Mammary Transcriptome

**DOI:** 10.1371/journal.pone.0032455

**Published:** 2012-03-16

**Authors:** Massimo Bionaz, Kathiravan Periasamy, Sandra L. Rodriguez-Zas, Walter L. Hurley, Juan J. Loor

**Affiliations:** 1 Department of Animal Sciences, University of Illinois, Urbana, Illinois, United States of America; 2 Institute for Genomic Biology, University of Illinois, Urbana, Illinois, United States of America; 3 Animal Production and Health Section, Seibersdorf Laboratories Joint FAO/IAEA Division of Nuclear Techniques in Food and Agriculture International Atomic Energy Agency, Vienna, Austria; 4 Division of Nutritional Sciences, University of Illinois, Urbana, Illinois, United States of America; University of South Florida College of Medicine, United States of America

## Abstract

The overrepresented approach (ORA) is the most widely-accepted method for functional analysis of microarray datasets. The ORA is computationally-efficient and robust; however, it suffers from the inability of comparing results from multiple gene lists particularly with time-course experiments or those involving multiple treatments. To overcome such limitation a novel method termed Dynamic Impact Approach (DIA) is proposed. The DIA provides an estimate of the biological impact of the experimental conditions and the direction of the impact. The impact is obtained by combining the proportion of differentially expressed genes (DEG) with the log2 mean fold change and mean –log P-value of genes associated with the biological term. The direction of the impact is calculated as the difference of the impact of up-regulated DEG and down-regulated DEG associated with the biological term. The DIA was validated using microarray data from a time-course experiment of bovine mammary gland across the lactation cycle. Several annotation databases were analyzed with DIA and compared to the same analysis performed by the ORA. The DIA highlighted that during lactation both BTA6 and BTA14 were the most impacted chromosomes; among Uniprot tissues those related with lactating mammary gland were the most positively-impacted; within KEGG pathways ‘Galactose metabolism’ and several metabolism categories related to lipid synthesis were among the most impacted and induced; within Gene Ontology “lactose biosynthesis” among Biological processes and “Lactose synthase activity” and “Stearoyl-CoA 9-desaturase activity” among Molecular processes were the most impacted and induced. With the exception of the terms ‘Milk’, ‘Milk protein’ and ‘Mammary gland’ among Uniprot tissues and SP_PIR_Keyword, the use of ORA failed to capture as significantly-enriched (i.e., biologically relevant) any term known to be associated with lactating mammary gland. Results indicate the DIA is a biologically-sound approach for analysis of time-course experiments. This tool represents an alternative to ORA for functional analysis.

## Introduction

The gold standard for the functional analysis of high-throughput datasets is the enrichment analysis, also called overrepresented approach or ORA [1]. To gain more mechanistic insights into the underlying biology, ORA analysis is often conducted to investigate whether gene sets associated with particular biological functions, for example, as represented by Gene Ontology (GO) annotations, are statistically overrepresented in the identified gene groups. In ORA the most commonly used statistical test is the hypergeometric or the binomal approximation [2]. The P-value indicates the probability of observing the same number or more genes in the list that pertain to the particular GO term by chance (relative to all the genes in the list and all the genes known to pertain to the GO term). When the proportion of genes associated with a particular biological term is higher than what is expected by chance, the biological term is considered to be “enriched” or “overrepresented” [3]. The null-hypothesis can be calculated using a 2×2 contingency table [3] in association with the above-mentioned statistical approaches. The enrichment of genes associated with a particular biological term is a strong indicator that the cells have changed the functions associated with the biological term in a non-random fashion (i.e., the cells attempt to change their biology through alteration of gene expression). This in turn indicates that the biological term is functionally-relevant under the conditions studied.

The ORA can provide a quick and reliable way to identify the most important biological terms in a list of annotated genes/proteins. However, the approach has several limitations [1], [4]. Among those it is particularly important to mention the marked effect of the gene list size on the final results; this in turn does not allow to compare results from different experimental conditions, as is the case with experiments including multiple treatments or time points [1]. Time-course experiments lend themselves to use of microarray analysis (or any high-throughput technique) because the dynamic nature of the changing transcriptome can be captured. This approach allows for the study of adaptations of the tissue to the environment in “real-time”; thus, one can infer the biological adaptations of the tissue using the transcriptome. Clearly, the inability of ORA to capture the dynamic changes in functions inferred by the transcriptome limits its use in high-throughput time-course experiments. As reported previously [1] there is an urgent need to develop a new approach to functional analysis of microarray datasets from time-course experiments.

In the present manuscript a novel method for functional analysis of high-throughput data that overcomes most of the limitations related to the application of ORA is proposed. The method is referred to as Dynamic Impact Approach or DIA. The DIA was validated using microarray data from a large time-course experiment of bovine mammary tissue during an entire lactation cycle. The criterion to validate the DIA was suggested by Huang Da et al. [1]: “The notion that the enriched terms [or results from any other approach, N/A] should make sense based on a priori biological knowledge of the study is the most important guideline […]”. Thus, the validation of any new functional analysis system has to be performed relative to established knowledge of the biological system. The mammary gland offers an excellent system to verify such new approaches because the main biological functions of this organ are well-established (i.e., production of milk). The results of DIA were compared to the well-known functions of the lactating mammary gland. Validation was further performed by comparing the results of DIA *vs*. the results from the ORA.

## Results and Discussion

The use of false discovery rate (FDR) [5] ≤0.001 for the overall time effect and a post-hoc P-value≤0.001 between each comparison uncovered >6,000 genes differentially expressed (DEG) (Figure S1), suggesting that the mammary gland transcriptome experiences a tremendous degree of adaptation during lactation, with some comparisons showing ca. 4,000 DEG (Table 1).

**Table 1:** Number of differentially-enriched functions and pathways in Ingenuity Pathway Analysis (IPA) using different false discovery rate (FDR) correction thresholds of the raw P-values.

	FDR cut off for Functions						FDR cut off for Pathways						DEG	
**Comparison**	***0.05***	***0.10***	***0.30***	***0.50***	***0.70***	***1.00***	***0.05***	***0.10***	***0.30***	***0.50***	***0.70***	***1.00***	**Overall**	**Eligible IPA**
–15 *vs.* –30	1	1	1	1	39	39	0	0	0	3	3	3	1320	696
1 *vs.* –30	0	0	0	0	0	42	0	0	8	12	29	38	3688	2027
15 *vs.* –30	0	0	0	1	39	39	0	0	0	0	0	10	2703	1507
30 *vs.* –30	0	0	0	0	40	40	0	0	0	2	2	2	3242	1877
60 *vs.* –30	0	0	0	0	0	39	0	1	1	2	10	32	3980	2175
120 *vs.* –30	0	0	0	0	0	37	0	0	0	0	8	9	3821	2092
240 *vs.* –30	1	6	21	36	36	36	0	1	2	13	21	91	1502	874
300 *vs.* –30	0	0	0	0	40	40	0	1	1	3	3	29	1522	843
1 *vs.* –15	0	0	7	10	40	40	0	0	0	9	18	38	2038	1198
15 *vs.* 1	0	0	3	42	42	42	0	0	0	0	0	0	1326	753
30 *vs.* 15	0	0	41	41	41	41	0	0	0	69	110	112	235	146
60 *vs.* 30	0	0	0	44	44	44	2	2	17	38	81	97	378	211
120 *vs.* 60	0	0	0	43	43	43	0	0	0	1	1	5	327	184
240 *vs.* 120	0	0	0	0	0	45	0	0	0	0	0	0	2027	1107
300 *vs.* 240	0	0	0	41	41	41	1	2	5	5	52	83	356	215

Reported is the number of functions, pathways and DEG enriched at the specified FDR cut-off. Legend: FDR = false discovery rate cut-off applied in the function/pathway analysis; DEG = differentially expressed genes, with “Overall” denoting the total number of DEG and “Eligible in IPA” the number of DEG eligible for function/pathway analysis in IPA.

### Data mining through enrichment analysis

We used Ingenuity Pathway Analysis® (IPA) and Database for Annotation, Visualization and Integrated Discovery (DAVID) [6] for the functional analysis by means of a statistical over-representation approach [1]. The inability of the ORA to provide biologically-relevant information in our time-course experiment was exemplified in data from IPA reported in Table 1. Based on those data and using the Benjamini-Hochberg [5] multiple testing correction the number of significantly-enriched functions and pathways was minimal (Table 1). When this criteria was applied, despite having large transcriptomic differences between comparisons (>3,000 DEG), no (or very few for only a couple of comparisons) biological terms could have been considered significantly-enriched and, thus, discussed (Table 1).

Based on the results of the functional analysis using IPA (which relies on ORA; complete discussion of IPA results using the non-FDR corrected enrichment results are available in file S1) we concluded that the ORA is not an adequate approach for functional analysis in time-course experiments as also pointed out previously [1]. The ORA analysis can be successfully applied in time-course experiments or those involving multiple treatments only with gene lists that result from methods such as clustering or principal component analyses. Those methods allow reducing the dataset or the gene lists and the functional analysis can be performed with high confidence using the ORA. The primary objective of the combinations of those approaches is uncovering co-regulated genes/functions. However, because of the reduction of gene lists or separation of the dataset into smaller gene lists, those tools do not provide a holistic and integral view of the dynamism of impacted functions or pathways (or any biological term) through time.

### Dynamic Impact Approach **(**DIA**)**

#### Overview

**Figure 1:**
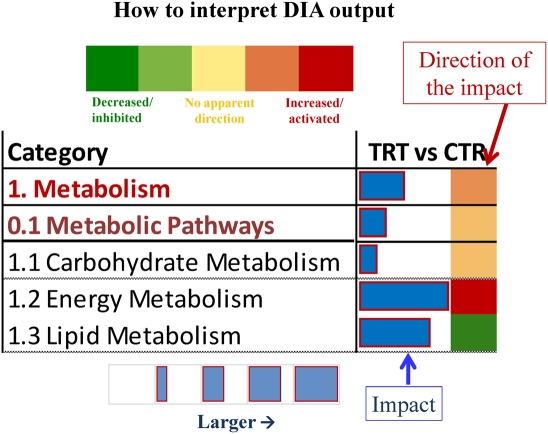
Visual explanation for interpretation of the Dynamic Impact Approach output. On the extreme left are reported the biological terms (in the present figure it is reported, for instance, the main categories of KEGG pathways). On the right of the column with the biological term is the column of the comparison considered. The column presents two sub-columns. In the left sub-column it is reported the horizontal blue bar that denotes the overall impact of the differentially expressed genes on the biological term. Larger the horizontal bar larger the impact. In the right sub-column it is reported a colored square that denotes the direction of the impact (green = inhibited/decreased; red = activated/increased). Darker the color larger the activation (if red) or inhibition (if green) of the biological term.

The DIA attempts to capture the biological *impact* of any condition as inferred through the transcriptome (or any other high-throughput technique) and to visualize the dynamism of such impact, especially through time. In addition, the DIA offers a way to interpret the biology of the impact by providing the *direction of the impact* (see Figure 1 for explanation of DIA outputs and their interpretation). The detailed description of the DIA is available in file S2.

Based on the criteria suggested previously [1] and reported above any reliable approach for functional analysis of the longitudinal bovine mammary microarray dataset should capture at the very least the main functions of the tissue during lactation; thus, the results from the DIA in the present study were consistently verified relative to prior knowledge of the bovine mammary gland. In addition, we have compared the results from the DIA with results using DAVID, a powerful web-based tool for functional analysis of microarray data that relies on ORA. The team that developed and maintains DAVID kindly provided the annotation databases used by DIA (with the exception of the KEGG pathways that were downloaded from the KEGG web-site, see file S2). The use of the same annotation databases by the two tools allowed for a meaningful comparison.

### Dynamic Impact of DEG on chromosomes

**Figure 2:**
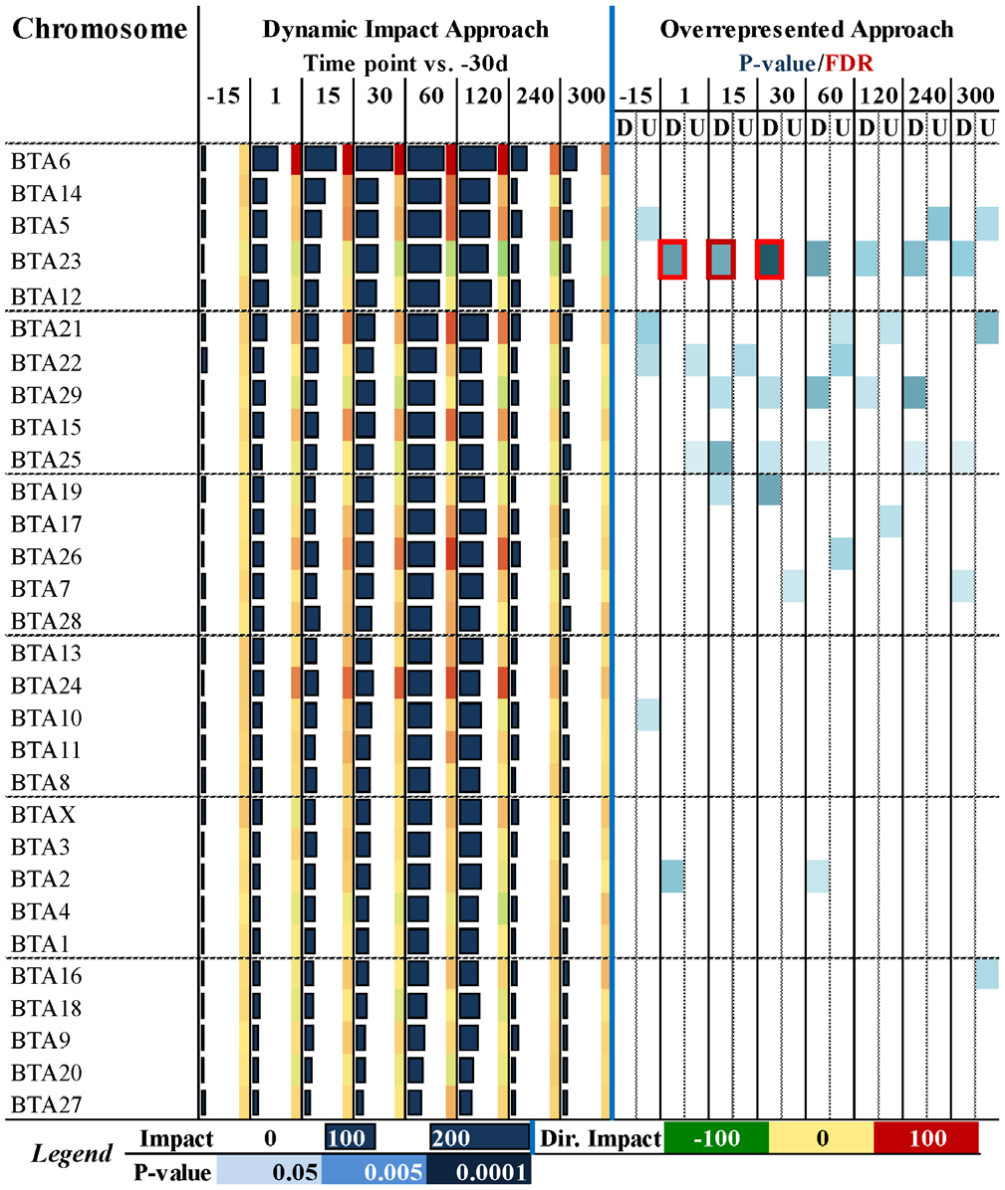
Dynamic impact approach (DIA) and overrepresented approach (ORA) results of DEG during lactation vs. −30d on bovine chromosomes. The chromosomes are sorted in descending order by the overall impact as calculated by the DIA during lactation (from 15 to 120 vs. –30d). For the DIA the horizontal bars denote the impact and the colored squares on the right denote the direction of the impact (red = increase; green = decrease) (see Figure 1 for detailed explanation). For the ORA reported are the results from analysis with DAVID of up-regulated and down-regulated DEG per each comparison. Highlighted in blue are the chromosome significantly enriched with P≤0.05 for the comparison (darker lower the P-value). Red squares denote a false discovery rate (FDR)≤0.05. The overall data indicate that BTA6 was the most impacted and activated chromosomes during lactation, followed by BTA14 and BTA5. The BTA23 was highly impacted but it appeared that transcription of the genes contained in this autosome was generally inhibited during lactation. This chromosome was also the only one showing a significant enrichment with a FDR≤0.05 in few comparisons.

Determining the impact of DEG on chromosomes can be useful to identify those regions experiencing greater (or lower) transcription during lactation (i.e., euchromatic vs. heterochromatic regions). This information can help in genetic selection and identification of genes in quantitative trait loci (QTL) regions. The impact and the direction of the impact of the DEG on bovine chromosome are reported in Figure 2.

The most-impacted chromosomes during lactation and with apparent greater overall activated transcribed regions were BTA6, BTA14, BTA5, and BTA21. Highly-impacted but with an overall decrease in transcription were BTA23 and BTA29. The BTA12 was among the most impacted but there was equilibrium between genes with an increase vs. a decrease in transcription. Since the first report of a QTL for milk production in dairy cows [7] the BTA6 has been confirmed as the chromosome with the greatest number of QTL regions [8], [9], [10]. The casein genes are present in BTA6. Those genes form a cluster in a relatively small region of BTA6 whose expression appeared to be under a *locus* control region that also includes statherin (Figure S2). Our results showed a large increase in expression of the statherin gene during lactation (file S3), confirming the *locus* control region regulation, as also reported previously [11]. Besides casein genes, BTA6 also contains a plethora of genes that have been shown to have a strong effect on selection for milk yield. For instance, *ABCG2* and *SPP1* have been demonstrated to be excellent candidate genes [12], [13]. The *ABCG2* is also one of the 2 QTN found thus far in bovine [14]. A detailed topological visualization of the relative impact of each gene in the BTA6 is reported in Figure S2 (for details see file S4).

The BTA14 has been recognized to have several QTL for milk traits [15], particularly for milk fat synthesis [16], [17], including genes such as *DGAT1* [14], [18]; in our microarray data *DGAT1* was not among the significant DEG at an FDR≤0.001 (but had an FDR = 0.03, see file S3), however, *DGAT1* was ca. 2-fold up-regulated during lactation when measured by qPCR [19]. Novel genes related to milk production were uncovered in this chromosome (see Figure S3 and file S4). The BTA5 also has been shown to harbor QTL regions for milk production [20], [21], such as *LALBA* (Figure S4 and file S4).

Our results suggested that transcription of most of the genes contained in the BTA23 decreases in order for the mammary tissue to initiate and carry out lactation (Figure 2). The BTA23 has not been previously considered as a QTL for milk traits in dairy cows. Most of the genes related to the MHC (both class I and II) are located in this autosome. In a region of the chromosomes that spans ca. 2,000,000 bp we observed 7 highly-impacted genes related to MHC that were inhibited during lactation; in addition, in a region spanning ca. 400,000 bp there were 4 consecutive genes belonging to the MHC class I that were significantly down-regulated during lactation (Figure S5 and details in file S4).

The BTA12 is associated with milk fat synthesis in Finnish Ayrshire dairy cattle [22], but no reports exist in Holstein cows. None of the highly-impacted genes during lactation in this chromosome have been reported previously to be related to milk synthesis (Figure S6 and file S4).

The results using the DIA are supported by previous QTL studies [10], providing evidence of the biological relevance of the approach. In addition, the DIA allowed identifying the most impacted genes in each autosome, uncovering a plethora of new genes which might be associated with milk traits and could be used to uncover new QTN [14]. In contrast, the use of ORA with a Benjamini-Hochberg FDR≤0.05 failed to uncover any of the previously-identified QTL chromosomes as being significantly-enriched, with only BTA23 significantly enriched at few time points during lactation and few other BTA enriched at a liberal EASE score [6] (Figure 2 and file S5 for details of direct comparisons between ORA and DIA).

### Tissue-specificity of the DEG

**Figure 3:**
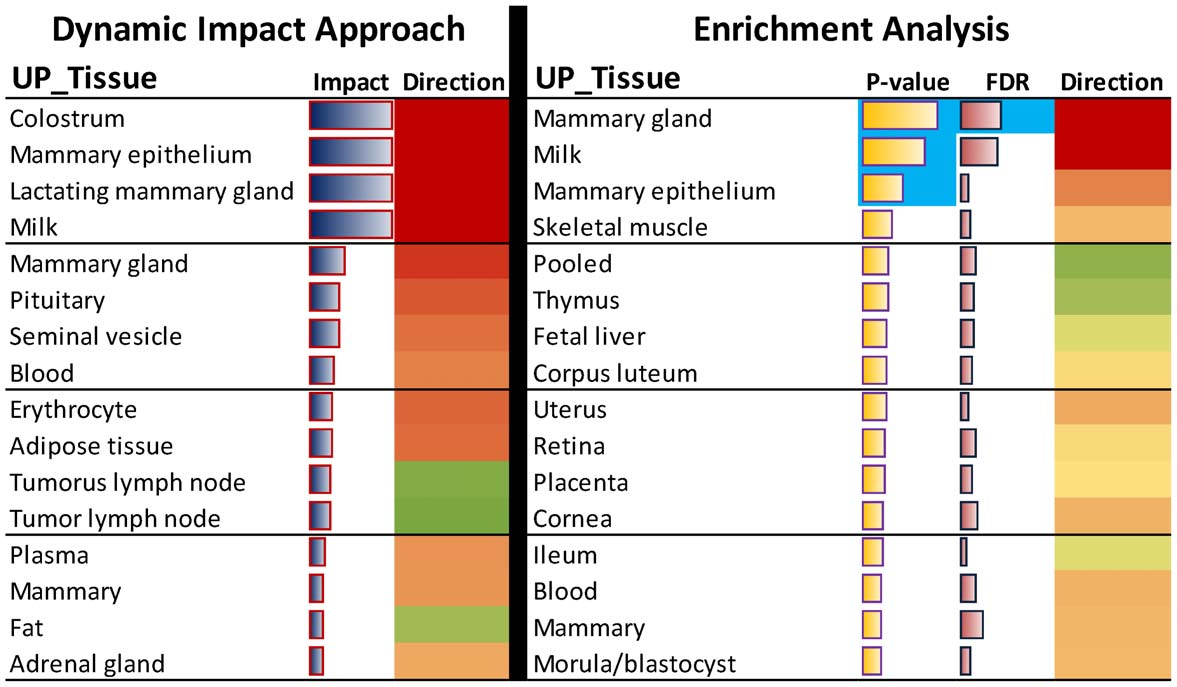
Direct comparison between the Uniprot Tissue analysis results from the Dynamic Impact Approach (DIA) and enrichment or overrepresented approach (ORA) during lactation (from 15 to 120 vs. −30d). Reported are the 16 top Uniprot Tissue term from each analysis. For the DIA shown are the tissue name, the average impact (horizontal blue bars) and direction of the impact (red = induced; green = inhibited) during lactation. For the ORA shown are the name of the tissue, the average P-value (i.e., average –log P-value; orange horizontal bars), the average Benjamini-Hochberg corrected P-value (i.e., average –log Benjamini-Hochberg corrected P-value or FDR; horizontal purple bars), and the estimated direction of the enrichment (i.e., [mean –log P-value enrichment of up-regulated DEG] – [mean –log P-value of down-regulated DEG]). In light blue shade are highlighted the tissues with a mean enrichment P-value or FDR≤0.05 (or –log P-value≥1.33).

In order to further test the validity of the DIA analysis we have performed an analysis to determine if the approach was able to capture the tissue-specificity of the significantly-affected transcriptome. To accomplish this we used the Uniprot tissue (UP_tissue) annotation database. The top 16 tissues are reported in Figure 3 for both the DIA and the ORA, additional data and comparisons are available in file S5 (sheet “UP TISSUE”).

From 15 to 300 vs. –30d and at 1 vs. –15d, the tissue types significant in ORA with a multiple testing correction (Benjamini-Hochberg or B-H) were ‘Mammary gland’ and ‘Milk’ (file S5). The former was the only one with a mean FDR<0.05 during the peak of lactation (Figure 3). The only other tissue types related to mammary and enriched significantly with an EASE score <0.05 was ‘Mammary epithelial’, enriched at few time comparisons (file S5) and with a mean enrichment P-value during peak lactation of <0.05 (Figure 3). No other tissue types related to mammary were significantly enriched (Figure 3 and file S5). The use of the mean –log10 P-value during lactation to rank the most enriched tissues uncovered, from the most to the least enriched, ‘Mammary gland’, ‘Milk’, and ‘Mammary epithelial’, followed by ‘Skeletal muscle’ and ‘Pooled tissue’ (Figure 3). The term ‘Mammary’ also was among the top 16 enriched UP-Tissue terms, but was not significantly enriched in any comparison. The DIA ranked ‘Colostrum’, ‘Mammary epithelial’, ‘Lactating mammary gland’, ‘Milk’, and ‘Mammary gland’ as the first 5 most impacted UP_tissue. The term ‘Mammary’ was among the top 16 impacted UP_tissue terms (Figure 3 and file S5). All those terms were clearly induced during lactation.

The data clearly showed that the DIA captured more accurately all the UP-tissue terms related to lactating mammary, while ORA failed to uncover several terms such as ‘Colostrum’ (DIA suggested to be important during the whole lactation) and ‘Lactating mammary’.

### Impact of DEG on KEGG pathways

The solids fraction of milk in dairy cows is composed in large part by three components: lactose, fat, and protein. Thus, we expected to find that the synthesis and secretion of lactose, fat, and protein were among the most relevant functions in the mammary gland in early lactation [23]. In addition, functions related to the immune system would be expected to appear due to the evolutionary origin and role of mammary gland in the immune system [24].

**Figure 4:**
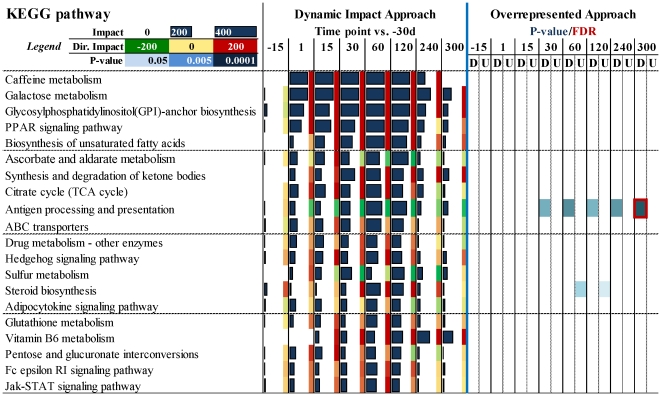
Twenty most impacted KEGG pathways during lactation (excluding the pathways in Human disease category, see file S5, ‘KEGG pathways’ sheet for details) compared to dry period (from 15 to 120 vs. −30d). For the Dynamic Impact Approach (DIA) the horizontal bars denote the impact and the colored squares on the right denote the direction of the impact (red = increase; green = decrease) (see Figure 1 for detailed explanation). For the overrepresented approach (ORA) reported are the results from analysis with DAVID of up-regulated and down-regulated DEG per each comparison. Highlighted in blue are the KEGG pathways significantly enriched with P≤0.05 for the comparison (darker lower the P-value). Red squares denote a false discovery rate (FDR)≤0.05.

The detail of each KEGG pathway is reported in file S5 (sheet “KEGG pathway”). To uncover the most impacted pathways during lactation we calculated and sorted the impact from 15 to 120 vs. –30d. Shown in Figure 4 are the 20 most impacted pathways during lactation (we have excluded from this figure the ‘Human Diseases’ category-related pathways due to the low biological significance of those in bovine mammary, but the results for this category is reported in file S5). The top 4 pathways were ‘Caffeine metabolism’, ‘Galactose metabolism’ (i.e., lactose synthesis), ‘Glycosylphosphatidylinositol(GPI)-anchor biosynthesis’, and ‘PPAR signaling’.

The ‘Caffeine metabolism’ pathway was highly impacted as a result of the large up-regulation in expression of xanthine dehydrogenase gene (*XDH*) (file S3). This protein is highly concentrated in the milk fat globule (MFG) [25], [26] but besides an essential role in milk fat secretion [25] additional functions of *XDH* remain poorly defined [26]. The *XDH* might participate in caffeine metabolism for the production of uric acid (dimethyluric acid) from xanthine in bovine mammary tissue; however, the amount of uric acid in bovine milk is relatively low and decreases as milk production increases [27]. Thus, the high impact of a pathway involving *XDH* can be considered biologically relevant in view of the importance of the gene's product for milk fat production.

**Figure 5:**
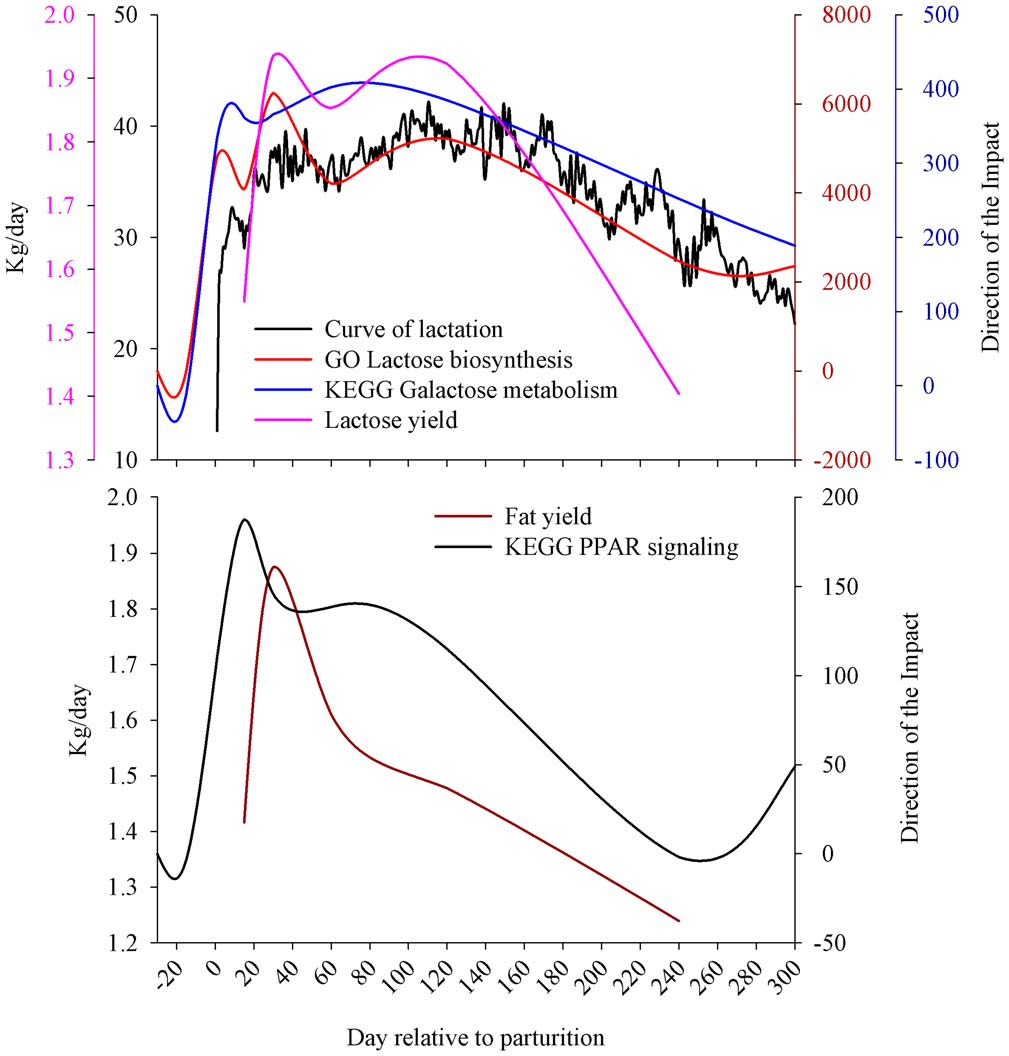
Upper panel: pattern of curve of lactation, milk lactose yield, and the direction of impact as calculated by the Dynamic Impact Approach of KEGG ‘Galactose metabolism’ pathway and GO Biological process ‘Lactose biosynthetic process’. Lower panel: pattern of milk fat yield and direction of the impact of KEGG ‘PPAR signaling’ pathway. The correlation was positive between the curve of lactation and lactose yield with ‘Galactose metabolism’ (r = 0.87, P = 0.02 and r = 0.81, P = 0.09, respectively) and ‘Lactose biosynthetic process’ (r = 0.82, P = 0.05 and r = 0.88, P = 0.05, respectively). The correlation was positive (r = 0.56) but not significant (P = 0.32) between milk fat yield and ‘PPAR signaling’.

The finding that ‘Galactose metabolism’ was the most impacted and induced pathway during lactation (after ‘Caffeine metabolism’) was striking because lactose synthesis has been demonstrated to be the most important metabolic event determining milk yield [28]. The production of transgenic sows with bovine alpha-lactalbumin and over-expression of alpha-lactalbumin in mice showed a significant increase in milk synthesis without changing milk composition [29], [30], [31], [32]. Increased expression of milk proteins or other enzymes involved in milk synthesis has not been reported to increase milk yield (e.g., [33]). To our knowledge, the only transgene that resulted in an increase in milk yield besides alpha-lactalbumin is the overexpression of IGF1 in mouse [34]. However, overexpression of IGF1 in sows did not increase milk yield [35]. The relationship between direction of the impact of ‘Galactose metabolism’ and curve of lactation is reported in Figure 5. The figure reveals a high similarity in the pattern between the direction of the impact of the ‘Galactose metabolism’ KEGG pathway and milk production. A simple Pearson correlation analysis between the direction of the impact of the ‘Galactose metabolism’ and the mean curve of lactation was significant (r = 0.87; P = 0.02), while with the mean lactose yield (data previously reported, see [36]) it tended to be positively correlated (r = 0.81; P = 0.09). Those data indicated that the DIA was able to capture as the most relevant pathway the one known to be essential for milk yield.

The importance of peroxisome proliferator-activated receptor (PPAR), particularly PPARγ, in the mammary gland has already been suggested by our group in previous studies [19], [37]. The large impact and induction of ‘PPAR signaling’ during lactation, which was inferred from the DIA analysis, was due to large and significant up-regulation of PPARγ target genes, such as *CD36*, *FABP3*, *FABP4*, and *LPL* (see file S3). This result supports our previous data [19], [37] pointing to PPARγ as an important player in milk fat synthesis regulation in the bovine mammary gland. In support of this conclusion, the time fluctuation of the direction of the impact of ‘PPAR signaling’ closely resembled the one of milk fat yield, with an apparent “anticipation” of the ‘PPAR signaling’ pathway over the milk fat yield (Figure 5; the Pearson correlation was not significant with r = 0.56 and P = 0.32).

The high impact of ‘Glycosylphosphatidylinositol(GPI)-anchor biosynthesis’ uncovered by DIA is a novel finding and is discussed in detail in the companion paper. Other pathways among the most impacted and induced were those related to ‘Lipid metabolism’ (particularly ‘Biosynthesis of unsaturated fatty acids’, ‘Synthesis and degradation of ketone bodies’, and ‘Steroid biosynthesis’), ‘TCA cycle’, ‘ABC transport’, ‘Hedgehog signaling’, and ‘Glutathione metabolism’, and a few were inhibited, including ‘Antigen processing and presentation’ (Figure 4). The importance of those metabolic and signaling pathways is discussed in detail in the companion paper as we attempt to consider an integrative approach. However, pathways such as the ones related to lipid metabolism were expected, mostly considering the substantial amount of milk fat produced by the mammary gland (Figure 5). The large impact of ‘Biosynthesis of unsaturated fatty acids’ is a well-established and important phenomenon in mammary tissue during lactation (e.g., [38]).

With the exception of ‘Steroid biosynthesis” and ‘Antigen processing and presentation’ in few comparisons, when the KEGG pathways were analyzed using the ORA none of the above pathways came up significant (Figure 4). In addition, considering an FDR cut-off of 0.05, only ‘Ribosome’ and ‘Antigen processing and presentation’ were significantly enriched, the former in up-regulated DEG before parturition and the latter in down-regulated DEG at the end of lactation (Figure 4 and file S5). Even applying an EASE score of 0.05 provided few significantly enriched pathways and almost none of the enriched pathways known to be important in lactating mammary gland (Figure 4 and file S5).

In summary the DIA was able to capture as most biologically-relevant some of the KEGG pathways expected to be central for milk synthesis. The importance of lactose synthesis was supported by ‘Galactose metabolism’, the importance of milk fat synthesis by ‘PPAR signaling’, ‘Biosynthesis of unsaturated fatty acids’, and other pathways related to lipid synthesis, the importance of the immune system was supported by the large impact but inhibition of ‘Antigen processing and presentation’. The only biological phenomena mentioned above to be expected but not captured by the DIA was ‘milk protein synthesis’. The regulation of protein synthesis in bovine mammary is complex [36] and a detailed discussion is outside the scope of the present paper. A discussion of protein synthesis using qPCR data was presented previously [36]. The discussion of microarray data related to protein synthesis is provided in the companion paper.

### Most impacted Gene Ontology **(**GO**)** terms

**Figure 6:**
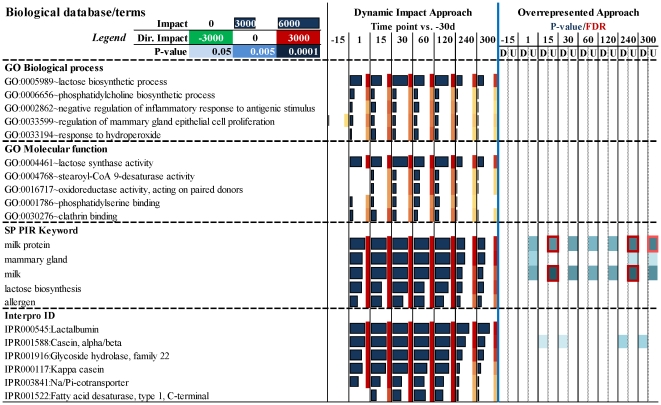
Most impacted GO Biological process terms, Swiss-Prot (SP) and Protein Information Resource (PIR) Keywords (SP_PIR_KEYWORD), InterPro ID, and Protein Information Resource (PIR) superfamily (PIR_SUPERFAMILY) during lactation (from 15 to 120 vs. −30d) as calculated by the Dynamic Impact Approach and the results from the overrepresented approach using DAVID of the same terms (see file S5 for results of all terms and results from additional annotation databases).

The rank of the 10 most impacted GO Biological process, GO Molecular function, Swiss-Prot (SP) and Protein Information Resource (PIR) Keywords (SP_PIR_KEYWORD), and InterPro ID is reported in Figure 6. Results of additional database annotations are reported in file S5. The DIA was able to capture GO Biological processes which can be easily expected in lactating mammary gland, for instance ‘Lactose biosynthesis’ was the most impacted and activated term during lactation. The direction of the impact of this term and the milk and lactose yield (data available in [36]) were positively correlated (r>0.81; P<0.05; Figure 5). Other top GO Biological process terms uncovered by the DIA also confirmed previous data. The production of phospholipids in bovine mammary during lactation is known to be high and indispensable for the synthesis of the fat globule membrane [39]. This finding supports the high impact of GO ‘Phosphatidylcholine biosynthesis process’ term (Figure 6). The significance of other top GO Biological process terms is described in the companion paper where a more integrative approach is undertaken.

Among GO Molecular function the DIA ranked as the most impacted ’Lactose synthase activity’ and ‘Steroyl-CoA 9-desaturase activity’. The importance of lactose synthesis was discussed above. The role of the Steroyl-CoA 9-desaturase activity in mammary has been previously discussed [19]. The inhibition of this enzyme using sterculic acid had a significant negative effect on milk fat synthesis [40], supporting a crucial role of this enzyme for milk synthesis. The importance of the ‘Clathrin binding’ together with the ‘Phosphatidylserine binding’ for the lactating mammary gland appears evident considering that mammary gland during lactation secretes milk components through vesicles [41], [42], [43], [44].

Obvious terms such as ‘Milk protein’, ‘Milk’, and ‘Lactose biosynthesis’, but also others such as ‘Fatty acid desaturase’ were uncovered by DIA as top SP-PIR-Keyword and InterPro terms [19], [40] (Figure 6). Among SP-PIR-Keyword, use of ORA uncovered as significantly enriched ‘Milk protein’ and ‘Milk’; also with an EASE score ≤0.05 the term ‘Mammary gland’ was overrepresented when applying ORA. For the latter the significance was only at 1 vs. –30d and at the end of lactation (Figure 6). None of the other terms uncovered by DIA as the top terms were significantly enriched using ORA (Figure 6). In addition, as seen with the IPA analysis, very few terms overall were significantly-enriched at a Benjamini-Hochberg FDR correction ≤0.05 using the ORA. Most of those were almost exclusively found at –15, 240 and 300 vs. –30d when the number of DEG was the lowest (Table 1). There was also significant enrichment of terms related to translation at –15 vs. –30d and terms related to antigen processing and presentation at the end of lactation (file S5).

### Conclusions

Overall, our analysis indicated that the DIA is a reliable method to uncover known biological functions affected during lactation in bovine mammary (e.g., BTA6 and 14 among chromosomes, all mammary related UP_terms during lactation, and lactose synthesis in other databases) and appears to have outperformed the ORA. Overall, this tool represents an alternative to ORA for functional analysis of time-course experiments or those involving multiple treatments.

## Materials and Methods

The materials and methods concerning the ethic statement, the animal and sampling, the RNA extraction and microarray data, and milk yield and composition are described in details in the companion paper.

### Enrichment or overrepresentation approach **(**ORA**)**

The ORA analysis was run using Ingenuity Pathway Analysis (IPA) (Ingenuity Systems, http://www.ingenuity.com, Redwood City, CA) and the free Database for Annotation, Visualization and Integrated Discovery (DAVID) v6.7 (http://david.abcc.ncifcrf.gov/). Details of the methods and criteria used are reported in file S2.

### Dynamic Impact Approach for data mining

The Dynamic Impact Approach or DIA is based on a calculated impact and the direction of the impact (i.e., induced/increased or inhibited/decreased) of DEG on the biological terms (e.g., pathways, functions, and other terms; see file S2 for details). The rationale of the method lies on the fact that in a cell the change in transcriptome expression is non-random. From this assumption the change in flux of a metabolic or a signaling pathway is determined by the significant change and magnitude of the proteins involved in the pathway (see details of assumptions and rationale for the DIA in file S2).

Based on the above rationale, we propose that the transcriptome allows us to infer that a specific condition impacts a pathway (or any biological term) by examining 1) the proportion of transcripts coding for proteins involved in such pathway that are deemed to be significantly affected by the treatment, 2) the average magnitude of the effect (i.e., fold change) on transcript expression, and 3) the average significance of the effect on the transcripts coding for proteins composing the pathway. Based on the above rationale the **impact** and the **direction of the** **impact** are calculated as:

***Impact =*** *[Proportion of DEG in the pathway (corrected by the number of genes in the pathway present in the array or background)]×[average log2 fold change of the DEG]×[average of –log P-value of the DEG]*

***Direction of the Impact*** *= Impact of up-regulated DEG – Impact of down-regulated DEG*

The DIA was implemented using MS Excel and calculations run automatically (for Gene Ontology or very large annotated biological terms the use of 64 bit version is required or a “manual” protocol can be run). Complete description of the methods is reported in file S2. Few criteria need to be selected for data analysis by DIA. One of the most crucial, considering the calculations performed in the method, is the proportion of genes assigned to a biological term (i.e., pathway or Gene Ontology term) that are present in the microarray used relative to the total number of genes assigned to the term. This is important in order to increase biological relevance of the results and to avoid discussing terms that are not appropriately covered by the platform used. For all databases analyzed, except KEGG pathways, we have used a cut-off of ≥30% genes present on our microarray platform vs. genome. For the analysis with KEGG pathways the cut-off was ≥40%. This more stringent criterion was deemed necessary due to the lower number of genes considered in KEGG pathways relative to other databases such as Gene Ontology or Chromosomes.

## Supporting Information

Figure S1 **Overall view of the 6,382 microarray targets with FDR≤0.001 and P-value<0.001 in at least one comparison during lactation in bovine mammary tissue**. Image generated with GeneSpring GX7.
(TIFF)

Figure S2 **Location and relative impact** (**average log2 fold change relative to −30d from 1 to 120 day in milk × −log10 post-hoc P-value**) **of genes in BTA6 with an overall significant change at FDR<0.001 and a P-value<0.001 in at least one time point comparison**. In the box are shown the details about the region from 88,291,573 to 88,534,470 where the genes for caseins and *STATH* are located. Arrows highlight genes with the largest impact. Among 106 annotated genes differentially expressed due to lactation in this chromosome, 64 were down-regulated (average -1.6 calculated impact) and 42 up-regulated (average 13.1 calculated impact). For simplicity, the strand direction is not reported. The chromosome location was double-checked using Bovine Genome Browser (Baylor 4.0/bosTau4). Additional details are available in file S4.
(TIF)

Figure S3 **Location and relative impact** (**average log2 fold change relative to −30d from 1 to 120 day in milk × −log10 post-hoc P-value**) **of genes in BTA14 with an overall significant change at FDR<0.001 and a P-value<0.001 in at the least one time point comparison**. Arrows highlight genes with the largest impact. Among 93 annotated genes differentially expressed due to lactation, 46 were down-regulated (average –1.3 calculated impact) and 47 up-regulated (average 3.9 calculated impact). For simplicity, strand direction is not reported. The chromosome location was double-checked using Bovine Genome Browser (Baylor 4.0/bosTau4). Additional details are available in file S4.
(TIF)

Figure S4 **Location and relative impact** (**average log2 fold change relative to −30d from 1 to 120 day in milk × −log10 post-hoc P-value**) **of genes in BTA5 with an overall significant change at FDR<0.001 and a P-value <0.001 in at the least one time point comparison**. Arrows highlight genes with the largest impact. Among 215 annotated genes differentially expressed due to lactation 110 were down-regulated (average –2.0 calculated impact) and 115 up-regulated (average 5.2 calculated impact). For simplicity, strand direction is not reported. The chromosome location was double-checked using Bovine Genome Browser (Baylor 4.0/bosTau4). Additional details are available in file S4.
(TIF)

Figure S5 **Location and relative impact** (**average log2 fold change relative to −30d from 1 to 120 day in milk × −log10 post-hoc P-value**) **of genes in BTA23 with an overall significant change at FDR<0.001 and a P-value <0.001 in at least one time point comparison**. Arrows highlight genes with the largest impact. Among 141 annotated genes differentially expressed due to lactation 94 were down-regulated (average –2.3 calculated impact) and 51 up-regulated (average 3.7 calculated impact). For simplicity, strand direction is not reported. The chromosome location was double-checked using Bovine Genome Browser (Baylor 4.0/bosTau4). Additional details are available in file S4. *denote genes not confirmed by qPCR.
(TIF)

Figure S6 **Location and relative impact** (**average log2 fold change relative to −30d from 1 to 120 day in milk × −log10 post-hoc P-value**) **of genes in BTA12 with an overall significant change at FDR<0.001 and a P-value<0.001 in at the least one time point comparison**. Arrows highlight genes with the largest impact. Among 78 annotated genes differentially expressed due to lactation 41 were down-regulated (average –2.6 calculated impact) and 37 up-regulated (average 4.0 calculated impact). For simplicity, strand direction is not reported. The chromosome location was double-checked using Bovine Genome Browser (Baylor 4.0/bosTau4). Additional details are available in file S4.
(TIF)

File S1 **Results and discussion from the enrichment analysis using Ingenuity Pathway Analysis** (**Ingenuity Systems at** http://www.ingenuity.com/index.html) **with a simple Fisher exact test P-value≤0.05.**
(DOC)

File S2 **Additional Materials and Methods**.
(DOC)

File S3 **Complete dataset and differentially expressed genes** (**DEG**) **with annotation and statistical results**.
(XLSX)

File S4 **Number of DEG, calculated impact per gene** (**average log2 fold change relative to −30d from 1 to 120 day in milk × −log10 post-hoc P-value**) **for BTA6, 14, 5, 23, and 12.** Reported are also the results from the enrichment analysis for each chromosome using DAVID.
(XLSX)

File S5 **Complete results from the time course experiment using Dynamic Impact Approach** (**DIA**) **and DAVID for DEG between each time point relative to −30d**. Reported are the results from the Chromosome, UP_tissue, KEGG pathways, Gene Ontology (Biological process, Cellular component, and Molecular function), SP PIR Keyword, Interpro, COGontology, PIRsuperfamily, SMART, SSF, and UP_Seq_Feature. For each database analyzed reported are the DIA results in descending order from the most to the least overall impacted (as average between ‘impact’ of all time point during lactation, i.e., from 15 to 120 vs. –30d) and the respective results of the enrichment analysis using DAVID both as simple EASE score and Benjamini-Hochberg false discovery rate. The file allows for a quick comparison between results using the two approaches.
(XLSX)
