# Supplementary figures and images for: A Novel Dynamic Impact Approach (DIA) for Functional Analysis of Time-Course Omics Studies: Validation Using the Bovine Mammary Transcriptome
Source: PLoS One. 2012 Mar 16;7(3):e32455. doi: 10.1371/journal.pone.0032455 (PMC3306320; doi:10.1371/journal.pone.0032455)

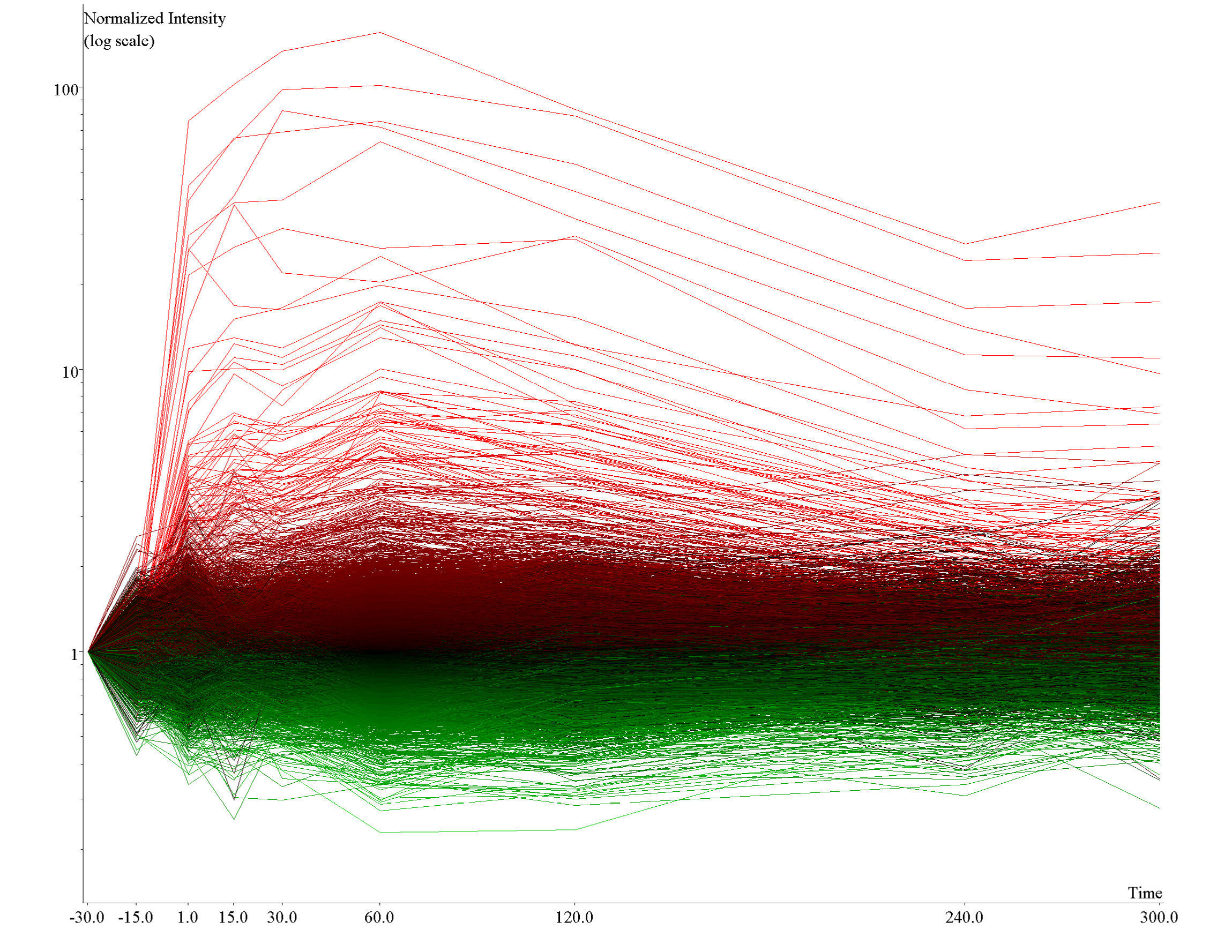

Supplement: Figure S1 — Overall view of the 6,382 microarray targets with FDR≤0.001 and P-value<0.001 in at least one comparison during lactation in bovine mammary tissue. Image generated with GeneSpring GX7. (TIFF) [file pone.0032455.s001.tif]

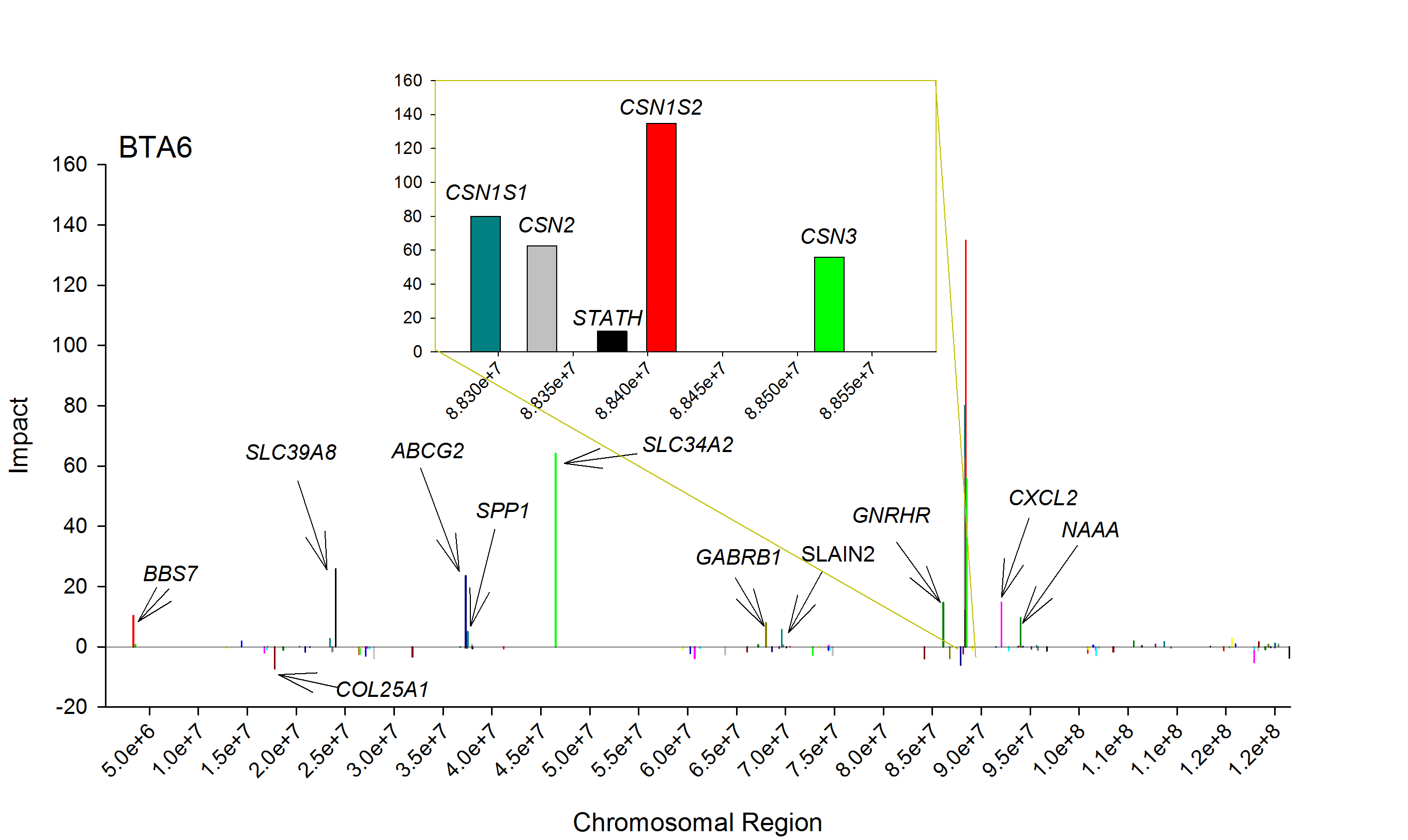

Supplement: Figure S2 — Location and relative impact (average log2 fold change relative to −30d from 1 to 120 day in milk × −log10 post-hoc P-value) of genes in BTA6 with an overall significant change at FDR<0.001 and a P-value<0.001 in at least one time point comparison. In the box are shown the details about the region from 88,291,573 to 88,534,470 where the genes for caseins and STATH are located. Arrows highlight genes with the largest impact. Among 106 annotated genes differentially expressed due to lactation in this chromosome, 64 were down-regulated (average -1.6 calculated impact) and 42 up-regulated (average 13.1 calculated impact). For simplicity, the strand direction is not reported. The chromosome location was double-checked using Bovine Genome Browser (Baylor 4.0/bosTau4). Additional details are available in file S4. (TIF) [file pone.0032455.s002.tif]

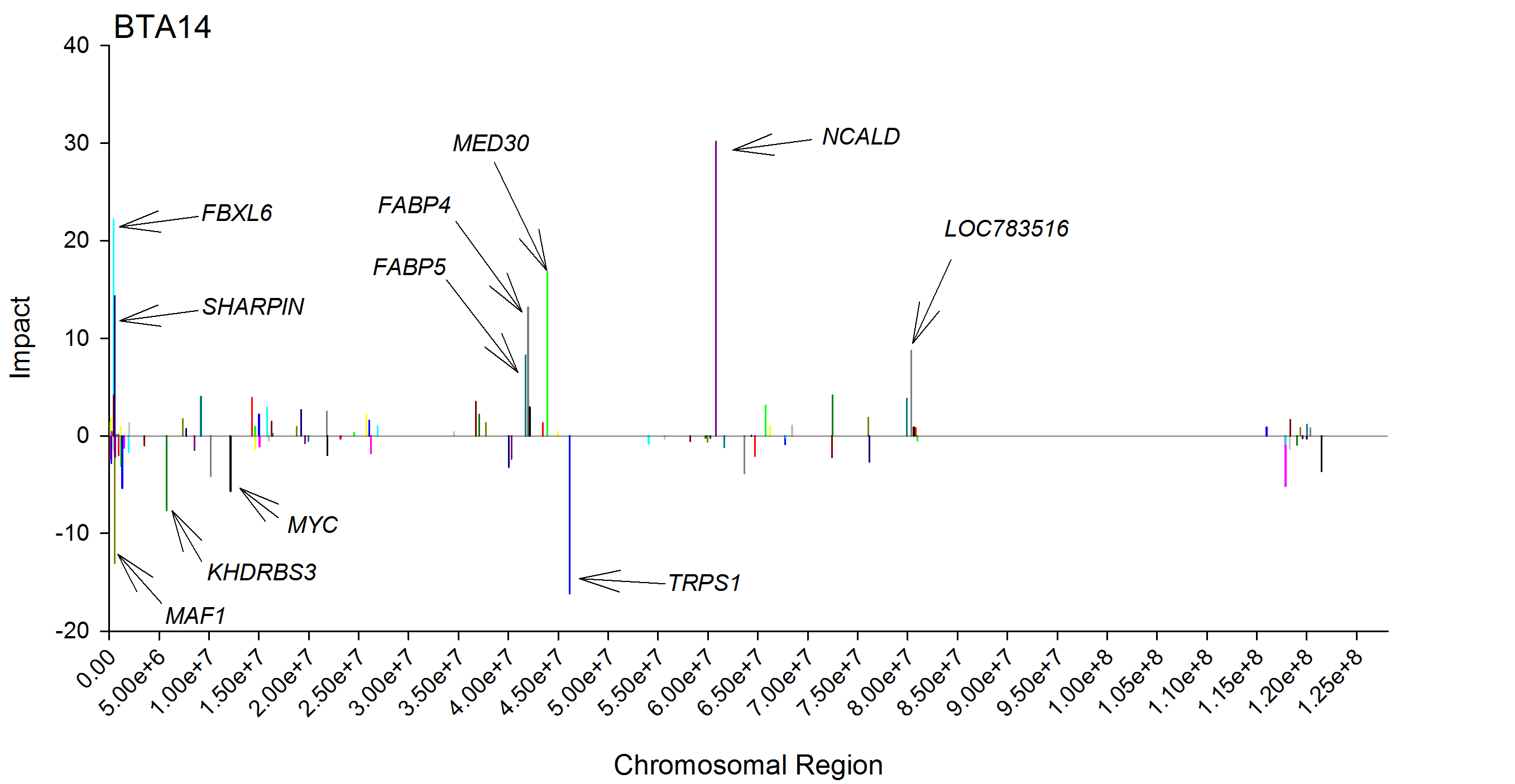

Supplement: Figure S3 — Location and relative impact (average log2 fold change relative to −30d from 1 to 120 day in milk × −log10 post-hoc P-value) of genes in BTA14 with an overall significant change at FDR<0.001 and a P-value<0.001 in at the least one time point comparison. Arrows highlight genes with the largest impact. Among 93 annotated genes differentially expressed due to lactation, 46 were down-regulated (average –1.3 calculated impact) and 47 up-regulated (average 3.9 calculated impact). For simplicity, strand direction is not reported. The chromosome location was double-checked using Bovine Genome Browser (Baylor 4.0/bosTau4). Additional details are available in file S4. (TIF) [file pone.0032455.s003.tif]

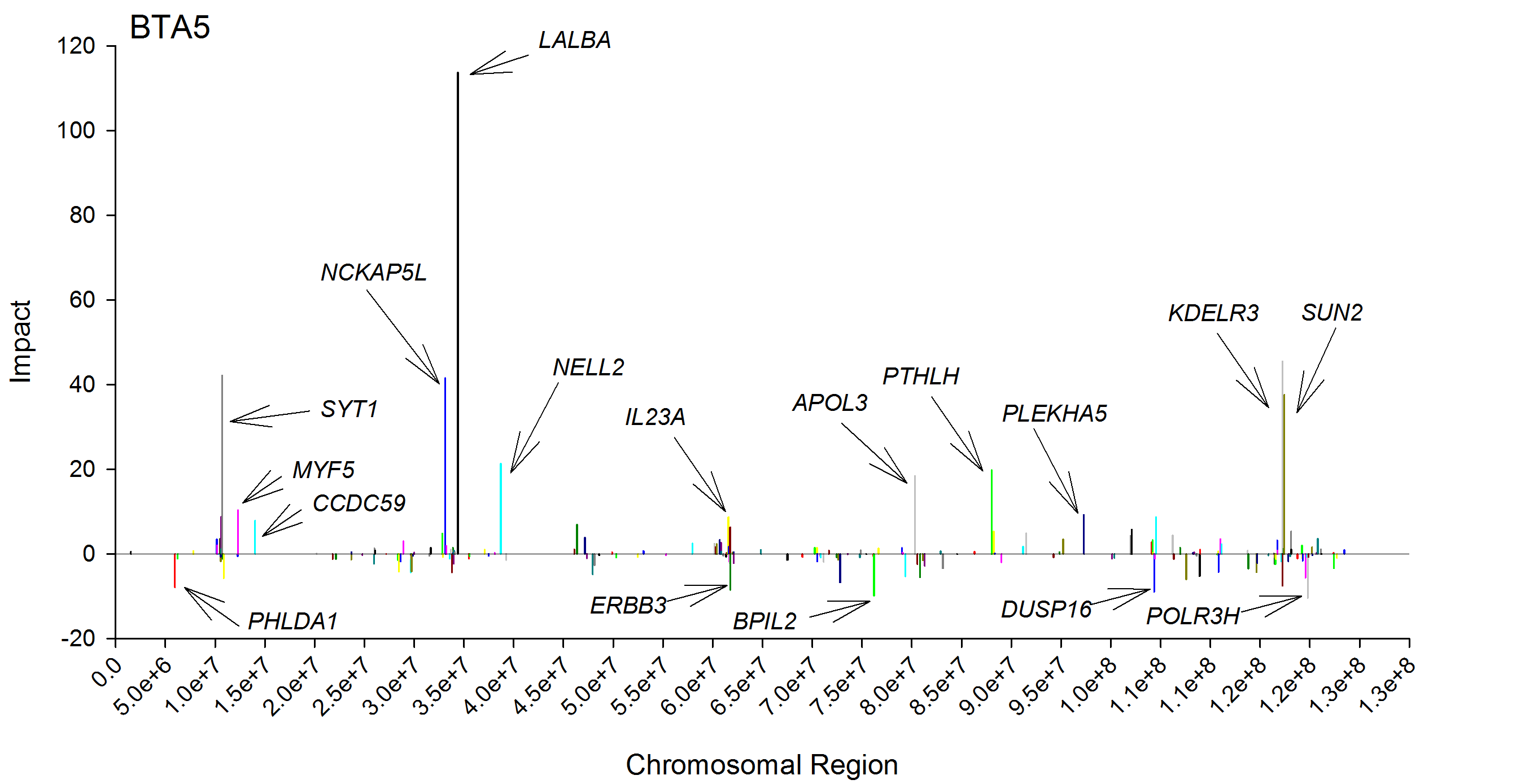

Supplement: Figure S4 — Location and relative impact (average log2 fold change relative to −30d from 1 to 120 day in milk × −log10 post-hoc P-value) of genes in BTA5 with an overall significant change at FDR<0.001 and a P-value <0.001 in at the least one time point comparison. Arrows highlight genes with the largest impact. Among 215 annotated genes differentially expressed due to lactation 110 were down-regulated (average –2.0 calculated impact) and 115 up-regulated (average 5.2 calculated impact). For simplicity, strand direction is not reported. The chromosome location was double-checked using Bovine Genome Browser (Baylor 4.0/bosTau4). Additional details are available in file S4. (TIF) [file pone.0032455.s004.tif]

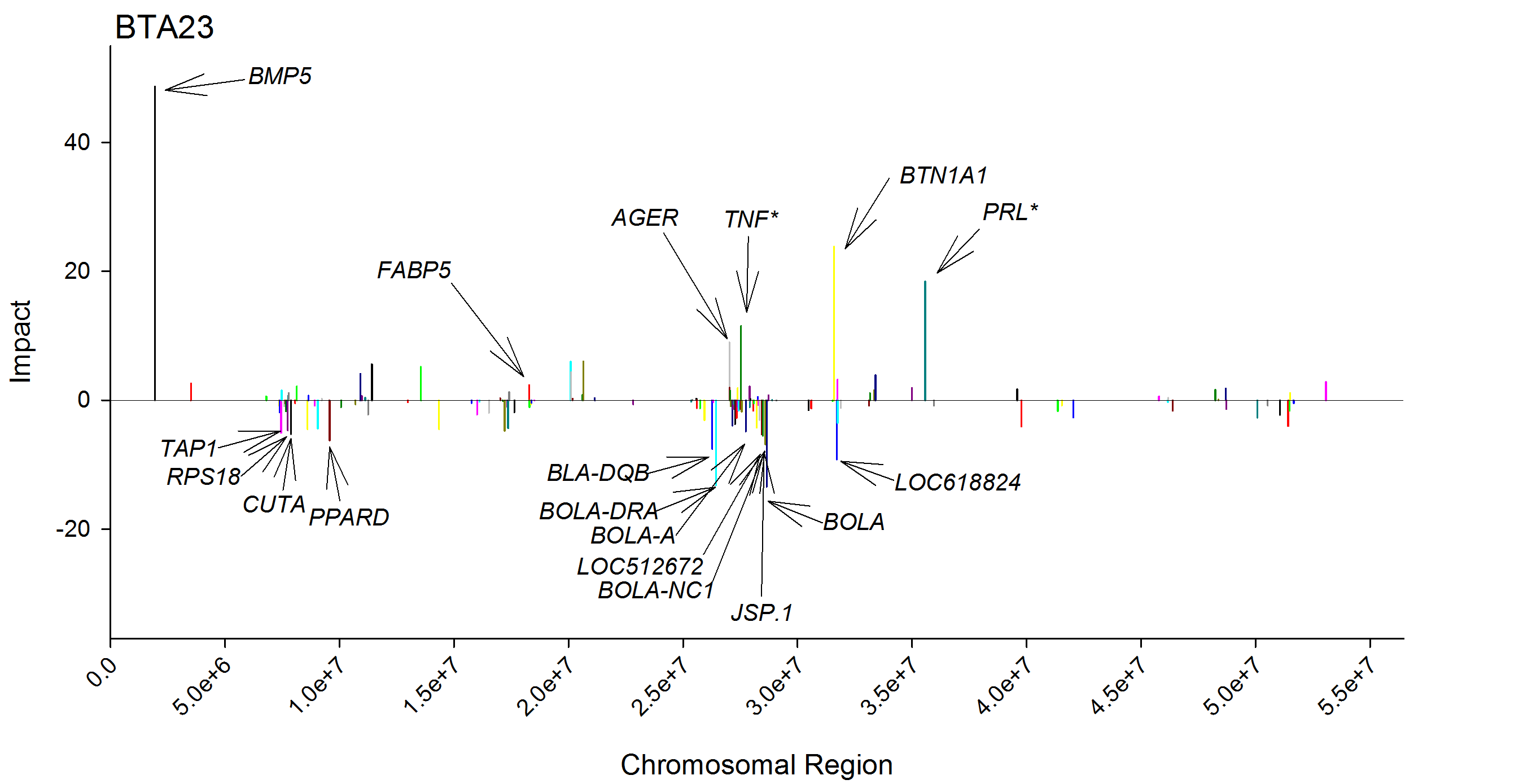

Supplement: Figure S5 — Location and relative impact (average log2 fold change relative to −30d from 1 to 120 day in milk × −log10 post-hoc P-value) of genes in BTA23 with an overall significant change at FDR<0.001 and a P-value <0.001 in at least one time point comparison. Arrows highlight genes with the largest impact. Among 141 annotated genes differentially expressed due to lactation 94 were down-regulated (average –2.3 calculated impact) and 51 up-regulated (average 3.7 calculated impact). For simplicity, strand direction is not reported. The chromosome location was double-checked using Bovine Genome Browser (Baylor 4.0/bosTau4). Additional details are available in file S4. *denote genes not confirmed by qPCR. (TIF) [file pone.0032455.s005.tif]

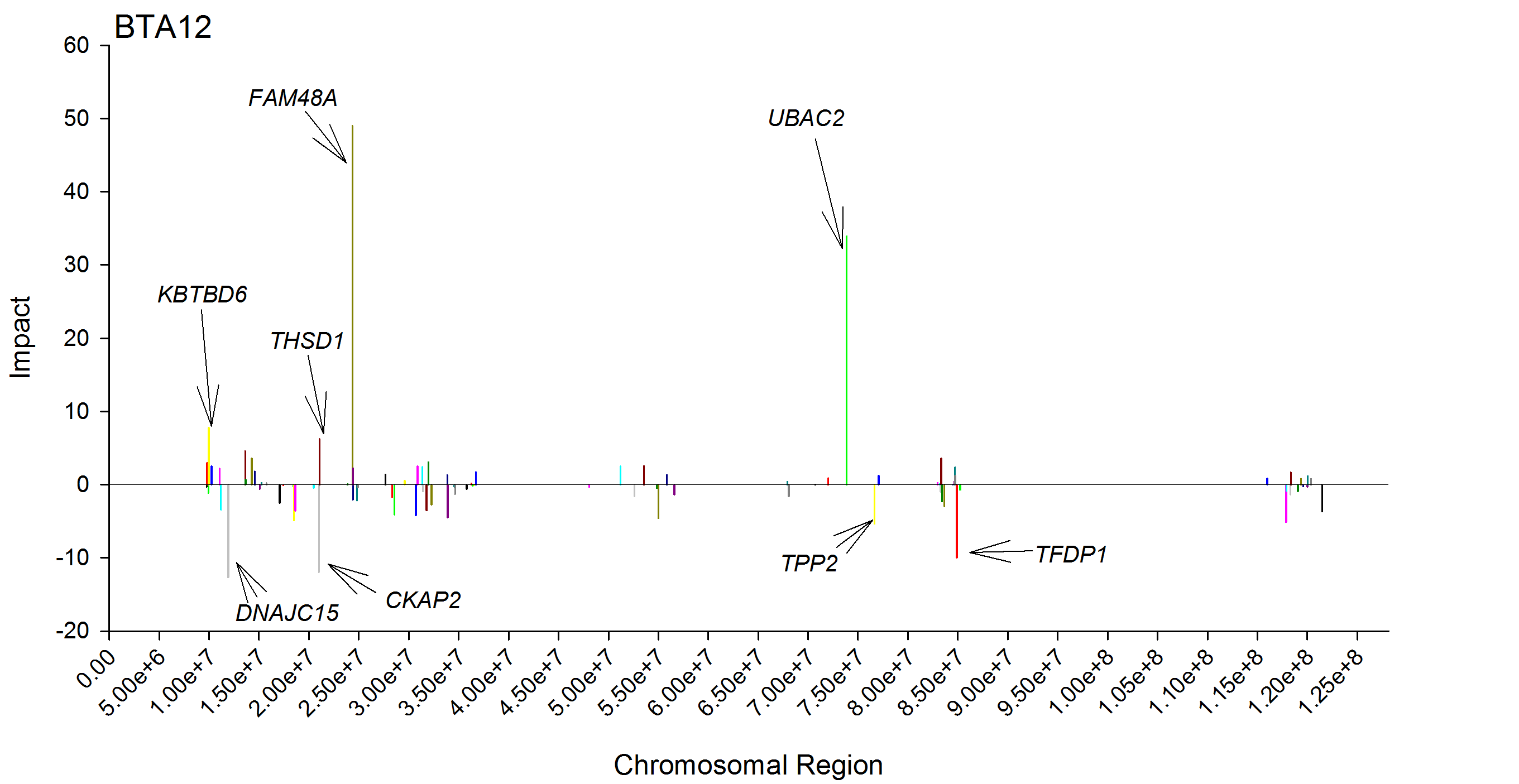

Supplement: Figure S6 — Location and relative impact (average log2 fold change relative to −30d from 1 to 120 day in milk × −log10 post-hoc P-value) of genes in BTA12 with an overall significant change at FDR<0.001 and a P-value<0.001 in at the least one time point comparison. Arrows highlight genes with the largest impact. Among 78 annotated genes differentially expressed due to lactation 41 were down-regulated (average –2.6 calculated impact) and 37 up-regulated (average 4.0 calculated impact). For simplicity, strand direction is not reported. The chromosome location was double-checked using Bovine Genome Browser (Baylor 4.0/bosTau4). Additional details are available in file S4. (TIF) [file pone.0032455.s006.tif]
