# Supplementary material for: A Novel Dynamic Impact Approach (DIA) for Functional Analysis of Time-Course Omics Studies: Validation Using the Bovine Mammary Transcriptome
Source: PLoS One. 2012 Mar 16;7(3):e32455. doi: 10.1371/journal.pone.0032455 (PMC3306320; doi:10.1371/journal.pone.0032455)
Supplement: File S1 — Results and discussion from the enrichment analysis using Ingenuity Pathway Analysis (Ingenuity Systems at http://www.ingenuity.com/index.html) with a simple Fisher exact test P-value≤0.05. (DOC) [file pone.0032455.s007.doc]

**INGENUITY PATHWAY ANALYSIS RESULTS**

Table of Contents

[1-Functional analysis of DEG using a FDR-corrected P-value = 0.10 3](#__RefHeading___Toc300669405)

[1.1-Unexpected observations and possible explanations 3](#__RefHeading___Toc300669406)

[2.1-Biological significance of enriched functions/pathways at an FDR = 0.10 5](#__RefHeading___Toc300669407)

[2-Ingenuity Pathway Analysis (IPA) functions, Gene Ontology (GO) categories, and canonical pathways of overall DEG 6](#__RefHeading___Toc300669408)

[3-Functions, canonical pathways, and GO categories of DEG at each time point relative to -30 d and in consecutive time points: dynamic adaptation of the transcriptome 6](#__RefHeading___Toc300669409)

[3.1. Methods in data mining 6](#__RefHeading___Toc300669410)

[Ingenuity Pathway Analysis (IPA) 6](#__RefHeading___Toc300669411)

[Gene Ontology (GO) 7](#__RefHeading___Toc300669412)

[Comparison analysis 7](#__RefHeading___Toc300669413)

[Technical limitations in data mining 7](#__RefHeading___Toc300669414)

[3.2- Main considerations 7](#__RefHeading___Toc300669415)

[3.3. Metabolic adaptations of mammary tissue inferred by combinatory analysis of IPA and GO 15](#__RefHeading___Toc300669416)

[Lipid synthesis 15](#__RefHeading___Toc300669417)

[Protein synthesis 15](#__RefHeading___Toc300669418)

[Other relevant metabolism. 16](#__RefHeading___Toc300669419)

[Transport 17](#__RefHeading___Toc300669420)

[Drug metabolism 17](#__RefHeading___Toc300669421)

[3.4. Mammary cell development during end of pregnancy and lactation 18](#__RefHeading___Toc300669422)

[Cell cycle and cell death 18](#__RefHeading___Toc300669423)

[Cell growth and proliferation 19](#__RefHeading___Toc300669424)

[DNA replication, recombination, and repair 19](#__RefHeading___Toc300669425)

[Gene expression 20](#__RefHeading___Toc300669426)

[Cellular assembly and organization 21](#__RefHeading___Toc300669427)

[Cell-to-cell signaling and interaction 22](#__RefHeading___Toc300669428)

[3.5. General physiological system development and functional adaptation of mammary gland from pregnancy to lactation 23](#__RefHeading___Toc300669429)

[Cell/tissue morphology and development. 23](#__RefHeading___Toc300669430)

[The immune system 23](#__RefHeading___Toc300669431)

[Cardiovascular system development and function 25](#__RefHeading___Toc300669432)

[Connective tissue development and function 25](#__RefHeading___Toc300669433)

[Nervous system development and function 26](#__RefHeading___Toc300669434)

[Endocrine system development and function 26](#__RefHeading___Toc300669435)

[REFERENCES 59](#__RefHeading___Toc300669436)

# 1-Functional analysis of DEG using a FDR-corrected P-value = 0.10

## 1.1-Unexpected observations and possible explanations

The correction of P-values of functions/pathways in Ingenuity Pathways Analysis (IPA) and gene ontology (GO; using GeneSpring GX10) by a False Discovery Rate (FDR) = 0.05 provided only 2 enriched functions and 3 pathways in IPA among all comparisons tested. Significantly enriched functions in IPA were protein synthesis in the comparison -15 vs. -30d and Cell Growth and Proliferation in the comparison 240 vs. -30d. When in IPA the FDR was set at 0.10, only 1 function in the comparison -15 vs. -30d and 6 functions (besides Cell Growth and Proliferation also enriched were Cellular Movement, Cell-to-cell Signaling and Interaction, Tissue Development, Cardiovascular System Development and Function, and Tissue Morphology; all those functions appeared to be induced) in the comparison 240 vs. -30d were significant (Table R1).

The pathway analysis from IPA provided only 7 pathways being significant with an FDR = 0.10 (Keratan Sulfate Biosynthesis at 60 vs. -30d; Tight Junction Signaling at 240 vs. -30d; Antigen Presentation Pathway at 300 vs. -30d; Cyanoamino Acid Metabolism and Glutathione Metabolism at 60 vs. 30d; Mitochondrial Dysfunction at 300 vs. 240d). Interestingly, the comparisons vs. -30d with larger number of DEG eligible for function/pathway analysis in IPA (e.g., 1 vs. -30d, 60 vs. -30d, and 120 vs. -30d) tended to have a lower number or no functions and/or pathways significant when an FDR was applied (Table R1).

A similar low number of significantly-enriched functions/pathways were observed with GO analysis (Table 2).

Those results were unexpected even tough in a previous study dealing with data from mouse mammary gland encompassing pregnancy, lactation, and involution IPA analysis of a list of genes resulted from a principal component analysis did not find any significant pathways enriched at an FDR = 0.05 or P < 0.05 .

**TABLE R1.** Number of differentially-enriched functions and pathways in Ingenuity Pathway Analysis (IPA) using a threshold of FDR correction for the P-value. In gradient of blue, purple, and orange are highlighted from lower (light color) to greater (dark color) number of functions, pathways and DEG enriched at the specified FDR cut-off.

DIM = day in milk or relative to parturition; FDR = false discovery rate applied in the function/pathway analysis; DEG = differentially expressed genes, with “Overall” denoting the total number of DEG and “Eligible in IPA” the number of DEG eligible for function/pathway analysis in IPA.

|  |  | **FDR cut off for Functions** | | | | | | **FDR cut off for Pathways** | | | | | | **DEG** | |
| --- | --- | --- | --- | --- | --- | --- | --- | --- | --- | --- | --- | --- | --- | --- | --- |
| **vs.** | **DIM** | **0.05** | **0.10** | **0.30** | **0.50** | **0.70** | **1.00** | **0.05** | **0.10** | **0.30** | **0.50** | **0.70** | **1.00** | **Overall** | **Eligible in IPA** |
| **-30** | **-15** | 1 | 1 | 1 | 1 | 39 | 39 | 0 | 0 | 0 | 3 | 3 | 3 | 1316 | 696 |
|  | **1** | 0 | 0 | 0 | 0 | 0 | 42 | 0 | 0 | 8 | 12 | 29 | 38 | 3677 | 2027 |
|  | **15** | 0 | 0 | 0 | 1 | 39 | 39 | 0 | 0 | 0 | 0 | 0 | 10 | 2691 | 1507 |
|  | **30** | 0 | 0 | 0 | 0 | 40 | 40 | 0 | 0 | 0 | 2 | 2 | 2 | 2023 | 1077 |
|  | **60** | 0 | 0 | 0 | 0 | 0 | 39 | 0 | 1 | 1 | 2 | 10 | 32 | 3969 | 2175 |
|  | **120** | 0 | 0 | 0 | 0 | 0 | 37 | 0 | 0 | 0 | 0 | 8 | 9 | 3808 | 2092 |
|  | **240** | 1 | 6 | 21 | 36 | 36 | 36 | 0 | 1 | 2 | 13 | 21 | 91 | 1492 | 874 |
|  | **300** | 0 | 0 | 0 | 0 | 40 | 40 | 0 | 1 | 1 | 3 | 3 | 29 | 1515 | 843 |
| **-15** | **1** | 0 | 0 | 7 | 10 | 40 | 40 | 0 | 0 | 0 | 9 | 18 | 38 | 2031 | 1198 |
| **1** | **15** | 0 | 0 | 3 | 42 | 42 | 42 | 0 | 0 | 0 | 0 | 0 | 0 | 1322 | 753 |
| **15** | **30** | 0 | 0 | 41 | 41 | 41 | 41 | 0 | 0 | 0 | 69 | 110 | 112 | 229 | 146 |
| **30** | **60** | 0 | 0 | 0 | 44 | 44 | 44 | 2 | 2 | 17 | 38 | 81 | 97 | 377 | 211 |
| **60** | **120** | 0 | 0 | 0 | 43 | 43 | 43 | 0 | 0 | 0 | 1 | 1 | 5 | 324 | 184 |
| **120** | **240** | 0 | 0 | 0 | 0 | 0 | 45 | 0 | 0 | 0 | 0 | 0 | 0 | 2020 | 1107 |
| **240** | **300** | 0 | 0 | 0 | 41 | 41 | 41 | 1 | 2 | 5 | 5 | 52 | 83 | 353 | 215 |

**TABLE R2.** Gene Ontology analysis results with an FDR = 0.10. The analysis was run using GeneSpring GX10 and shown are the number of genes only in comparisons with at least 1 significant GO term. In pink shade are Biological Functions and in light-blue shade are Cellular Components.

“All” denotes GO analysis using all DEG, “up” denotes GO analysis of up-regulated DEG, and “down” denotes GO analysis of down-regulated DEG.

|  |  | **Relative to -30d** | | | | | | | **Vs. previous time point** | | |
| --- | --- | --- | --- | --- | --- | --- | --- | --- | --- | --- | --- |
|  | **GO categories** | **-15** | | **1** | **120** | | **240** | **300** | **1vs-15** | | **240vs120** |
|  |  | ***all*** | ***up*** | ***down*** | ***all*** | ***down*** | ***up*** | ***up*** | ***up*** | ***down*** | ***up*** |
| **Biological Process** | translation | 50 | 48 |  |  |  |  |  |  |  |  |
|  | cellular macromolecule biosynthetic process | 50 | 48 |  |  |  |  |  |  |  |  |
|  | cellular protein metabolic process | 50 | 48 |  |  |  |  |  |  |  |  |
|  | macromolecule biosynthetic process |  | 48 |  |  |  |  |  |  |  |  |
|  | RNA splicing |  |  |  |  |  |  |  |  |  | 40 |
|  | RNA binding | 65 | 51 |  |  | 102 |  |  |  | 72 | 64 |
|  | nucleic acid binding |  |  |  |  | 160 |  |  |  | 112 | 106 |
| **Cellular Component** | extracellular region |  |  |  | 72 |  | 68 | 45 |  |  |  |
|  | membrane |  |  |  |  |  |  |  | 231 |  |  |
|  | integral to membrane |  |  |  |  |  |  |  | 158 |  |  |
|  | organelle |  |  |  |  |  |  |  |  | 345 |  |
|  | intracellular membrane-bounded organelle |  |  |  |  |  |  |  |  | 326 |  |
|  | intracellular non-membrane-bounded organelle |  | 43 |  |  |  |  |  |  | 23 |  |
|  | ribonucleoprotein complex | 54 | 51 |  |  |  |  |  |  | 31 |  |
|  | cytosolic ribosome | 31 | 30 |  |  |  |  |  |  | 1 |  |
|  | ribosomal subunit | 32 | 31 |  |  |  |  |  |  |  |  |
|  | large ribosomal subunit | 17 | 17 |  |  |  |  |  |  |  |  |
|  | cytosolic small ribosomal subunit | 15 | 14 |  |  |  |  |  |  |  |  |
|  | macromolecular complex |  | 48 |  |  |  |  |  |  | 31 |  |
|  | proteinaceous extracellular matrix |  |  |  |  |  | 23 |  |  |  |  |
|  | nucleus |  |  | 461 |  |  |  |  |  | 326 | 284 |

The lack of enrichment of functions/pathways/GO during maximal perturbations in mammary tissue transcriptome is biologically a non-sense. The use a P-value and/or FDR-corrected P-value as the only criteria to uncover significant affected functions have been criticized . The overrepresented approach (ORA) or enrichment appears a reliable method to identify the most enriched functions if the analysis is performed in a relatively short single list of genes; however, the results not always make biological sense and need to be discarded. Given a set of differentially expressed genes, the ORA compares the number of differentially expressed genes found in each category of interest with the number of genes expected to be found in the given category just by chance (i.e., if the gene were chosen randomly among the available genes in the background) . If the observed number is substantially higher than expected by chance alone, the category is reported as significant or overrepresented or enriched. The ORA approach have several limitations, some discussed elsewhere , but the most crucial for us are the decrease likelihood of finding significant functions/pathways larger is the list of DEG and the strong dependence of the results on the gene list which in turn do not allow for a direct comparisons between gene list ; the latter is a extreme limitation in time course experiments. In previous work from our lab we found large number of significant affected functions/pathways with large gene list , which suggest that the limitation report above is only evident if the gene list is not over-enriched by any specific function/pathway. The reasons for a lack of significant enriched functions/pathways in the time course of the bovine mammary can be two-fold. First, as previously reported , results can be due to the lack of mammary gland annotation or functional studies. The field of breast cancer is without a doubt the most-studied in regarding to the mammary gland, and very few studies exist to date which takes into consideration the mammary gland in its normal physiological state during lactation, especially in ruminant species. The IPA knowledge base is built exclusively using non-ruminant (and particularly rodent and human) scientific information. Similar reasons can be put forward for Gene Ontology. Therefore, it is likely that the specific functions/pathways turned-on/off in bovine mammary gland during lactation are not significantly-enriched because of a limitation of specific studies and/or of the limitations of bioinformatics tools. A second explanation, which appear to be more likely, is inferred by the fact that we observed a large number of genes turned-on and -off during lactation, particularly at the onset and peak-plateau of copious milk synthesis (Table R1 above and Figure 1 in the main body of the manuscript), and we know that the mammary gland has an abrupt and extremely large change in biology from pregnancy to lactation; i.e., large functional adaptations occur during this transition period. Thus, it was odd and biologically nonsensical that there would be a lack of significantly-enriched functions/pathways in IPA and categories in GO during these large transcriptomics adaptations. The apparent “nonsensical” observation can be explained by the fact that many/most of the known biological functions/pathways are perturbed simultaneously during the transition from pregnancy to lactation. At the genomics level this phenomena will translate in changes in expression of many genes encompassing all functions. If this is the case, the false discovery rate analysis would be unable to find specific enriched functions among all functions with a uncorrected-P ≤ 0.05.

In front of a lack of significant enriched functions/pathways three alternative approaches can be attempted: 1) to use the reference set of the Ingenuity Knowledge Base (IKB) for the functions/pathways analysis instead of our platform (see Materials and Methods for details); 2) use a more stringent FDR for the microarray data and/or use a fold-change cut off in order to decrease the number of eligible genes for analysis (i.e., reduce the gene list will probably increase the likelihood to find significant enriched functions/pathways); All the above attempt would have introduced a bias into the dataset or would not have been adequate because they would defeat our original goal, i.e., use an unsupervised approach and provide a large view of the mammary biology. The first suggestion, even though it would result in many affected functions at an FDR ≤ 0.05, was not acceptable because our microarray has a bias towards some specific functions/pathways (e.g., Cellular Growth and Proliferation, Gene Expression). Further, those functions/pathways tend to be significantly-enriched in all IPA comparisons, especially the ones with the higher number of DEG, using the IKB as reference dataset. Those results with the IKB differed substantially from the IPA analysis using our bovine microarray platform as reference, thus, providing the reasoning for not using the IKB as reference dataset. However, the use of the correct background (i.e., all the possible genes available in the platform or chip) is essential for a correct use of ORA . The second suggestion (i.e. fold-change cut off or more stringent FDR for the gene lists) would have introduced a bias for several reasons, for instance the fold-change cut off is arbitrary. Furthermore, the fold-change does not account for functionality of the genes (e.g., a slight but significant change in expression of a transcription factor, which affect a plethora of downstream genes, would probably have a greater functional impact than a large change in expression of an enzyme composing a very large metabolic pathway) or the original mRNA abundance (i.e., intensity signal in the microarray; e.g., a very low expressed transcript can increase 10-fold, but the amount of mRNA undergoing translation would certainly be lower than that of a transcript with very high expression and a 1.2-fold change).

## 2.1-Biological significance of enriched functions/pathways at an FDR = 0.10

The few functions significantly enriched at an FDR = 0.10 suggest that the induction of protein synthesis before the onset of lactation and the remodeling of the mammary tissue during the decline of milk production were the two most important biological events in bovine mammary gland during the lactation cycle, at least at the transcriptomics level. The GO analysis supports IPA findings of the important increase in protein synthesis before the onset of lactation (i.e., -15 vs. -30d) but does not support the mammary tissue remodeling at 240 vs. -30d.

The GO analysis suggests that RNA metabolism was induced before onset of lactation (-15 vs. -30d) and at the transition from plateau to decline of milk production (240 vs. 120d), while it was inhibited at 120 vs. -30d and at the onset of lactation (1 vs. -15d). The GO cellular component analysis indicated that ribosomal complex for protein synthesis was induced at -15 vs. -30d, nucleus components were inhibited at 1 vs. -30d and onset of lactation (1 vs. -15d) and were induced at 240 vs. 120d, extracellular region components were important and induced at 120, 240, and 300 vs. -30d, and at the onset of lactation membrane components were increased while organelles and ribosomal components were decreased.

The pathway analysis of time points in lactation vs. -30d highlighted a reduction of keratan sulfate synthesis at 60d, a likely increase in tight junction signaling at 240d, and a strong inhibition of antigen presentation at 300d. During subsequent time points, analysis of pathways indicated a strong induction of cyanoaminoacid and glutathione metabolism at 60 vs. 30d and mitochondrial dysfunction (i.e., mitochondrial oxidation) at 300 vs. 240d.

Overall, the analysis using ORA and an FDR=0.10 suggested that protein synthesis with all its components is essential for preparing the mammary gland for lactation, at least from the beginning of the last month of pregnancy until 2 wks before parturition. Afterwards, when lactation had been established, this function and its components became less important, with an apparent decrease in the protein synthesis machinery (i.e., ribosomes) at the onset of lactation (1 vs. -15d). A second suggestion from the data is the importance of tissue restructuring for controlling milk production, particularly during decline of milk synthesis and secretion. A third suggestion is the decline of antigen presentation as lactation ends, which might shed light on the decrease of mastitis problems after the first month and at the end compared with the first month of lactation. A fourth suggestion is the increase of importance of membrane components for onset of milk synthesis and secretion, which is plausible considering the large membrane turnover of the mammary gland during lactation. In the same time-frame, the data suggest a decreased emphasis of the mammary tissue on the major nuclear components (e.g., chromatin), which might support a scenario where the chromatin remodeling is shut-down to allow for expression of lactation genes to be stable and controlled by hormonal/physiological conditions (e.g., nuclear receptor or transcriptional regulators). A fifth observation is the importance of extracellular components, particularly tight junctions, during late and at the end of lactation.

In the following sections are reported the results and discussion of the analysis using the uncorrected P-value ≤ 0.05 as the cut-off to identify significant enriched functions/pathways in IPA and GO.

# 2-Ingenuity Pathway Analysis (IPA) functions, Gene Ontology (GO) categories, and canonical pathways of overall DEG

The main functions and pathways of the 6,579 DEG from IPA analysis highlighted the large number of metabolic functions associated with the DEG, with cell death and protein synthesis as the most significantly enriched. Beside synthesis, also the post-translational modification and transport appeared highly effected. Genes involved in assembly and organization, development, and cell-to-cell signaling and interaction were highly enriched, as well genes involved in metabolism, particularly lipid and carbohydrates, but also amino acids and nucleotides such as RNA and DNA. Functions associated with the latter, such as gene expression and cell cycle, were also well enriched. Among physiological functions was striking the large enrichment of genes involved in development and function of connective, nervous, muscle, and endothelial tissues. Interesting was also the large abundance of genes involved in immune system, where in one side there was a large number of genes involved in its development, in the other side many genes were also involved in immune response and cell response to inflammatory challenge (e.g., cellular compromise and free radical scavenging).

In GO biological process analysis protein synthesis was the most significantly enriched, followed by regulation of actin polymerization, anti-apoptosis, and phosphate transport. Detailed analysis revealed that DEG involved in protein synthesis were mostly part of the synthetic machinery (i.e., ribosomal components) and regulation of translation (e.g., elongation factors). In addition also lipoprotein metabolism appeared to be enriched, together with post-translational modification and transport of protein. Development and morphogenesis were enriched, such as blood vessels, muscle, epithelial, and neural (mostly dendrite) tissue. Cell adhesion and cell-to-cell communication were significant in DEG (e.g., integrin-mediated signaling pathway and Ca-dependent cell adhesion). The most enriched organelle by DEG appeared to be mitochondria and lysosomes, particularly their organization and biogenesis, however, the same were not present among the significantly enriched cellular components. In fact, the most enriched cellular components were ribosomes, followed by MHC protein complex, Golgi, basement membrane, actin cytoskeleton, ER, extracellular matrix, and nucleus (e.g., chromatin). The analysis of GO molecular functions highlighted the significant enrichment of constituent of ribosome, followed by actin binding, Ser/Thr phosphatase activity, RNA binding, extracellular matrix structure constituent, syntaxin binding, chromatin and RNA binding, UDP-glucosyl- and -galactosyltransferase activity, and Mn and Ca binding among others. Several metabolic and signaling pathways were significantly (P ≤ 0.05) enriched in IPA: PIK3/AKT signaling, keratan sulphate, ERK/MAPK signaling, glutathione metabolism, N-glycan biosynthesis, aryl-hydrocarbon receptor signaling, actin cytoskeleton signaling, aminoacyl-tRNA biosynthesis, chondroitin sulphate biosynthesis, and axonal guidance signaling.

Those overall analyses allow highlighting the predominant functions affected by DEG, but, because of the impossibility of having a clear direction of the DEG, do not permit a clear biological interpretation of the results. For this reason are deemed necessary analyses which account for the temporal transcriptomics adaptation by direct comparison among time points in order to study particular adaptation but also the dynamics of functions through time.

# 3-Functions, canonical pathways, and GO categories of DEG at each time point relative to -30 d and in consecutive time points: dynamic adaptation of the transcriptome

## 3.1. Methods in data mining

Ingenuity Pathway Analysis (IPA). For IPA analysis (Ingenuity® Systems, www.ingenuity.com), all oligos on the array platform were uploaded into the software with associated annotation (when present), LSmean from SAS (after back-transformation), FDR, and post-hoc P-values for each comparison. A value of 1 was applied in the FDR column (to avoid being considered a “good” gene in IPA) to those oligos that did not pass the aforementioned criteria. This approach allowed us to use our platform as the reference set for statistical analysis of enriched function/pathways, thus, preventing biases toward over-or under-represented functions in the platform. Each annotated gene was mapped to its corresponding gene object in the IPA Knowledge Base. Several analyses were run with IPA (details for IPA analysis and interpretation of the functions significantly enriched are reported in Additional file 2):

- ***Function Analysis***. This analysis identified the biological functions that were most significant within the data set. Genes from the dataset that met the FDR ≤ 0.001 and post-hoc P-value < 0.001 cut-off and were associated with a biological function/s in the IPA Knowledge Base were considered for the analysis. One tail Fischer’s exact test was used to calculate a p-value to determine the probability that each biological function assigned to the specific data set was due to chance alone;
- ***Canonical Pathway Analysis***: Canonical pathway analysis identified those pathways from the IPA Knowledge Base that were most significant to the data set. Genes from the data set that met the FDR ≤ 0.001 and post-hoc P-value < 0.001 cut-off and were associated with a canonical pathway in the IPA Knowledge Base were considered for the analysis. The significance of the association between the data set and the canonical pathway was measured in two ways: 1) a Fischer’s exact test was used to calculate a p-value determining the probability that the association between the DEG and the canonical pathway is explained by chance alone; 2) a ratio of the DEG that maps to the pathway divided by the total number of genes that map to the canonical pathway.

Gene Ontology (GO). The GO analysis was performed by means of GeneSpring GX7 with the annotation updated on March 3, 2009 by the automatic annotation feature in GeneSpring GX7 using GeneBank accession numbers. Out of 13,257 total oligos, the updated GO had 7,710 annotated oligos with a Biological process, 7,765 with a Cellular component, and 8,327 with a Molecular function. The individuation of significantly (P < 0.05) enriched GO categories was obtained by the calculation of a hypergeometric p-value without multiple testing correction, which is a measure of the statistical significance of the overlap between the number of genes in the selected list and all the genes in the array assigned to that category (i.e., the likelihood that it is a coincidence that genes in the list were in both the gene list and the category).

Comparison analysis. This feature in IPA allowed for a quick view of the main functions/pathways affected in the comparisons between time points. It allowed graphical depiction of data using SigmaPlot 10.0.1. In order to facilitate interpretation of results, the IPA figures reported include only functions enriched in at the least one comparison with a P-value of 0.001 [-log P-value = 3] with few exceptions (see captions in the figures). For the comparative pathways, the figures reported in the main body of the paper include pathways of all comparison relative to -30d with at the least 2 comparisons having P-value value ≤ 0.05 [-log P-value = 1.3] and pathways with at least one comparison with a –log P-value ≥ 1.89; for comparison across subsequent time points reported in the main body are those pathways with at the least one comparison having –log P-value ≥ 1.89 (P ≤ 0.013). A similar comparison also was developed using GO results, where functions enriched with a P-value ≤ 0.01 in at the least 1 comparison are reported for GO Biological process.

Through the discussion we use **↑** when the overall function was **induced** and **↓** when the overall function was **inhibited** or **reduced.** The overall direction of the response was inferred using IPA through detailed analysis of functional annotations as reported in Table R4-R19. The **increase** or **decrease** in **gene expression** is denoted by **⇑** or **⇓**, respectively. For interpretation of the GO category analysis we relied on the combination of GO analysis of overall ⇑ and ⇓ DEG.

Technical limitations in data mining. Several limitations exist for data mining. The most striking are the one discussed in the previous section; however, additional limitations are present such as the lack of mammary specific annotations as recognized previously, as well as lack of a complete bovine annotation. Additional limitations were present within IPA, which is based on human and rodent literature, and GO, which is neither bovine- nor mammary-specific. Besides bioinformatics-related limitations, our approach for interpretation of IPA and GO data can present limitations, because the thresholds used to judge the direction of a function (⇑ or ⇓) were selected by the authors on grounds that can be arguable. Completion of the bovine annotation in concert with refinement of bioinformatics tools could in the future provide new insights using the same data generated in the present experiment.

## 3.2- Main considerations

In Table R3 are reported the number of DEG eligible for IPA analysis for each comparison considered. In Figures R1 and R2 are reported the functions enriched with an exact Fisher test P-value ≤ 0.0015 in at the least one comparison by IPA analysis.

**TABLE R3**. Number of total differentially expressed genes (DEG; FDR ≤ 0.001 and post-hoc P < 0.001) in each comparison considered for the unsupervised analysis. Reported are the total numbers of DEG, up-regulated DEG, down-regulated DEG, % up-regulated in the total DEG, the number of DEG eligible for network and function/pathways analysis in Ingenuity Pathways Analysis® and the % relative to the total DEG.

| **Comparison** | | Total DEG | UP/DOWN | % UP on total | Network eligible | |  | Function/pathways eligible | |
| --- | --- | --- | --- | --- | --- | --- | --- | --- | --- |
|  |  |  |  |  | DEG | % total DEG |  | DEG | % total DEG |
| **Relative to -30d** | **-15** | 1316 | 753/563 | 57.2 | 832 | 63.2 |  | 696 | 52.9 |
|  | **1** | 3677 | 1752/1925 | 47.6 | 2411 | 65.6 |  | 2027 | 55.1 |
|  | **15** | 2691 | 1352/1339 | 50.2 | 1776 | 66.0 |  | 1507 | 56.0 |
|  | **30** | 2023 | 935/1088 | 46.2 | 1276 | 63.1 |  | 1077 | 53.2 |
|  | **60** | 3969 | 1863/2106 | 46.9 | 2579 | 65.0 |  | 2175 | 54.8 |
|  | **120** | 3808 | 1748/2060 | 45.9 | 2456 | 64.5 |  | 2092 | 54.9 |
|  | **240** | 1492 | 764/728 | 51.2 | 1021 | 68.4 |  | 874 | 58.6 |
|  | **300** | 1515 | 790/725 | 52.1 | 998 | 65.9 |  | 843 | 55.6 |
| **Relative to previous time point** | **1** | 2031 | 858/1173 | 42.2 | 1426 | 70.2 |  | 1198 | 59.0 |
|  | **15** | 1322 | 573/749 | 43.3 | 869 | 65.7 |  | 753 | 57.0 |
|  | **30** | 229 | 113/116 | 49.3 | 162 | 70.7 |  | 146 | 63.8 |
|  | **60** | 377 | 236/141 | 62.6 | 257 | 68.2 |  | 211 | 56.0 |
|  | **120** | 324 | 139/185 | 42.9 | 223 | 68.8 |  | 184 | 56.8 |
|  | **240** | 2020 | 1093/927 | 54.1 | 1285 | 63.6 |  | 1107 | 54.8 |
|  | **300** | 353 | 187/166 | 53.0 | 246 | 69.7 |  | 215 | 60.9 |
| **In lactation** | **UP** | 1028 |  | 49.5 | 673 | 65.5 |  | 570 | 55.5 |
|  | **DOWN** | 1050 |  |  | 693 | 66.0 |  | 591 | 56.3 |
|  | **BOTH** | 2078 |  |  | 1363 | 65.6 |  | 1158 | 55.8 |
|  | ***Average*** | ***1739*** |  | ***49.7*** | ***1141*** | ***66*** |  | ***968*** | ***56*** |
|  | ***SD*** | ***1183*** |  | ***5.6*** | ***767*** | ***2*** |  | ***648*** | ***3*** |

**FIGURE R1**. Functions significantly enriched in Ingenuity Pathway Analysis® (IPA) with an exact fisher test P-value ≤ 0.001 [-log(P-value) ≥ 3.0 – blue threshold in each graph] in at least one time point relative to -30d (except Immune Response and DNA Replication, Recombination, and repair, which had a max –log P-value <3.0). The Y-axis reports the -log P-value. Functions are listed in alphabetical order (except DNA Replication, Recombination, and Repair). Arrows denote a tendency to induce (**🡩**), induction (**🡹**), large induction (**🡹🡹**), tendency to inhibit (**🡫**), inhibition (**🡻**), large inhibition (**🡻🡻**), or equilibrium/absence of a net effect (**🡘**) of the function upon interpretation of IPA results as reported in detail in Additional Tables R4-R18.

**FIGURE R2**. Functions significantly enriched in Ingenuity Pathway Analysis® with an exact Fisher test P-value ≤ 0.001 [-log(P-value) ≥ 3.0– black threshold in each graph] in at least one time point relative to the previous time point (except Organ morphology and Reproductive System Development and Function). The Y-axis reports the -log P-value. Functions are reported in alphabetical order. Arrows denote a tendency to induce (**🡩**), induction (**🡹**), large induction (**🡹🡹**), tendency to inhibit (**🡫**), inhibition (**🡻**), large inhibition (**🡻🡻**), or equilibrium/absence of a net effect (**🡘**) of the function upon interpretation of IPA results.

**FIGURE R3**. Canonical pathways enriched in Ingenuity Pathway Analysis® (IPA) with an exact Fisher test P-value ≤ 0.05 [-log P-value ≥ 1.3] in at the least 2 comparisons vs. -30d or with a –log P-value ≥ 1.89 in at the least one comparison vs. -30d. The Y-axis reports the -log P-value (e.g., P < 0.05 = -log P > 1.3). Canonical pathways are reported in alphabetical order. The fill of the symbol denotes strong activation (dark red), activation (light red), tendency for activation (light red edge and white fill), strong inhibition (dark green), inhibition (light green), tendency to be inhibited (light green edge and white fill), and no apparent activation/inhibition of the pathway (black edge and white fill). 🞭 symbol denotes the ratio DEG/genes composing the pathways in the IPA database.

**FIGURE R4**. Canonical pathways significantly enriched in Ingenuity Pathway Analysis® (IPA) with an exact Fisher test P-value ≤ 0.013 [-log(P-value) ≥ 1.89] in at the least one time point relative to the previous time point. The Y-axis reports the -log P-value (e.g., P < 0.05 = -log P > 1.3). Canonical pathways are reported in alphabetical order. The fill of the symbols denote strong activation (dark red), activation (light red), tendency for activation (light red edge and white fill), strong inhibition (dark green), inhibition (light green), tendency to inhibit (light green edge and while fill), and no apparent activation/inhibition of the pathway (black edge and while fill). 🞭 symbols denote the ratio DEG/genes composing the pathways in the IPA database.

**FIGURE R5**. Most representative Gene Ontology (GO) Biological processes (68 out of 424) among those significantly (unadjusted P-value ≤ 0.05) enriched in the comparison of time points vs. -30d. For each comparison, the left column denotes the overall behavior of genes in the specific biological process (⇑ red, ⇓ green, and ⇔ [i.e. balance] if no color is shown; see Additional file 2 for detailed explanation) and the right column reports the number of DEG enriched in the process. The GO Biological processes are clustered in pre-selected categories by the authors to simplify interpretation of the data.

**FIGURE R6**. Most representative Gene Ontology (GO) Biological processes (63 out of 568) among those significantly (unadjusted P-value ≤ 0.05) enriched in the comparison between subsequent time points. For each comparison, the left column denotes the overall behavior of genes in the specific biological process (⇑ red, ⇓ green, and ⇔ [i.e. balance] if no color is shown; see Additional file 2 for detailed explanation) and the right column reports the number of DEG enriched in the process. The GO Biological processes are clustered in pre-selected categories by the authors to simplify interpretation of the data.

The overall comparison between functions uncovered by IPA with a significant (P ≤ 0.0015) enrichment in all time points relative to -30d (Figure R1) clearly highlighted the very large number of significant functions (16 out of 24) at 240d relative to all the other time points vs. -30d, followed by 15d (10), 30d (9), and 60d (9). In this regards it is interesting the fact that at 1 vs. -30d, despite the very large amount of DEG and number of functions enriched with a P-value ≤ 0.05, had cell death as the only function enriched with a P ≤ 0.0015.

In figure R3 and R4 are reported the most enriched canonical pathways from IPA. Beside IPA also most enriched (unadjusted P-value ≤ 0.05) biological process in GO analysis are reported in figures R5 and R6.

## 3.3. Metabolic adaptations of mammary tissue inferred by combinatory analysis of IPA and GO

The overall analysis of bovine mammary tissue uncovered a significant enrichment of functions related to metabolism including lipid and protein, carbohydrate, and nucleic acid, but also additional metabolic functions (denoted by “small molecular biochemistry” in IPA) and molecular transport.

Lipid synthesis**.** The mammary gland of rodents has been defined as a “lipid synthesizing machine” . Our data also support such a view for the cow because *lipid metabolism* was markedly ↑ between -15 to 300d, with a large ↑ during the first 30 days of lactation. Within IPA analysis, the most affected functions related to lipid metabolism were glycerols (particularly triacylglycerol or TAG), phospholipids, ceramide, and glycolipids (Table R4-R18). The GO analysis suggested a high ↑ of membrane lipid biosynthesis.

An in-depth analysis of TAG and ceramide synthesis by bovine mammary gland during lactation was presented previously . The current data revealed a biphasic pattern for lipid metabolism, with higher catabolic utilization of fatty acids (FA) between -30 and -15d, strong anabolic utilization of FA between 1 and 240d, and a return to catabolism of FA at the end of lactation (Table R4-R18). Particularly striking was the large ↑ in biosynthesis of lipids between -15 and 1d. Thus, besides the large increase in FA uptake during lactation (see below and ) the mammary gland channeled most, if not all, the FA toward synthesis of lipids. This finding is not novel . The utilization of FA for phospholipid synthesis is not surprising due to the large turnover/removal of cellular membranes within mammary tissue during lactatogenesis/galactopoiesis . The importance of ceramide synthesis during milk fat synthesis has been previously discussed .

The increase in anabolic activity of bovine mammary gland during the beginning of lactation, and particularly the increase in synthesis of lipids, has been established from more than half a century (e.g., see ). Even though the first large microarray analysis in mammary gland was performed, to our knowledge, by our group in a study of transcriptional changes in liver and mammary during the periparturient period , recently, another microarray analysis of mammary tissue during late prepartum and early postpartum has expanded on our initial analysis and includes gene ontology categories among affected genes .

Analysis with IPA uncovered 2 canonical pathways related to lipid metabolism that were significantly (P ≤ 0.05) affected in at the least one comparison vs. -30d (fatty acid biosynthesis, glycerolipid metabolism). Among those, FA biosynthesis was clearly ↑ between -15 and 1d and to some extent at 60 vs. -30d when several genes in the pathway were ⇓ but not *ACACA, FASN*, and *MCAT* with the formers being key for *de novo* FA synthesis . When consecutive time points were investigated, several pathways related to lipid metabolism appeared significantly affected (arachidonic acid metabolism and eicosanoids signaling, ceramide signaling, fatty acid biosynthesis, and glycerolipid and sphingolipid metabolism). The ↓ of arachidonic acid metabolism and effect on eicosanoids signaling were both significant between 30 and 60d, which together indicated utilization of phospholipids from cellular membranes to produce arachidonic acid. The latter seems to be utilized to synthesize leukotriene E4 which increases vascular permeability . Synthesis of other active lipids from arachidonate appeared to be inhibited. Interesting in this regard was the ↑ of DEG associated with histamine release during the same time frame, which has a similar effect on permeability of the vascular tissue .

The significant ↑ of mitochondrial dysfunction and oxidative phosphorylation between 240 and 300d are both indicative of ↑ utilization of lipid and glucose by mitochondria to produce energy, including FA oxidation. Glycerolipid metabolism was significant at 300 vs. -30d and between 15 and 30d. This pathway describes the formation of TAG and, even though it was not always detected as significant, it was present in all comparisons (except at 15 vs. -30d). The details of the pathway clearly suggested an ↑ in utilization of glycerides for formation of mono- and di-glycerides except at 300 vs. -30d. These indicated that the steps prior to the formation of TAG (i.e., mono- and di-glyceride) are more strictly under transcriptional regulation.

Protein synthesis**.** It was the most enriched function in both IPA and GO when all DEG with an FDR ≤ 0.001 were considered. This indicated a pivotal role of this function in the overall scheme of mammary tissue adaptations from pregnancy into lactation and throughout lactation. However, the temporal analysis in IPA showed that it was not directly affected as such, i.e., there was no evident effect on the protein synthesis machinery, but rather the activities related to protein synthesis such as protein modification and stabilization were highly affected and generally were ↓. In contrast, the GO analysis revealed that protein biosynthesis was highly induced from 1 mo to two weeks before parturition. Subsequently, there was a decrease from -15 to 15d, an induction at 1 relative to -30d, a slight increase again at 30 vs. -30d, and a decrease with a nadir at 120 vs. -30d. Interestingly, the Cellular components in GO analysis revealed a very large enrichment of ribosomal components at 2 weeks prior parturition, particularly cytosolic, which then decreased between -15 and 15d. There also was enrichment of organellar ribosomes, particularly mitochondrial, during lactation and especially at 30 vs. -30d. The enrichment of genes coding for the protein synthesis machinery (ribosomal protein and elongation factors) components also was evident from network analysis particularly at 2 wk before parturition.

The combination of GO and IPA analyses suggested a scenario where the protein synthesis machinery and its activity were largely ↑ at 2 weeks from parturition and gradually decreased during lactation through 120d. The ↓ in protein synthesis during lactation was strikingly similar to the one observed in mouse mammary gland during lactation and does not seem to support the apparent increase in protein synthesis suggested by Finucane et al. . In addition, data suggested an increase of mitochondrial protein synthesis during lactation.

Among pathways related with protein synthesis, Lys biosynthesis and Met metabolism were significantly enriched. These two amino acids (AA) are the most limiting for milk protein synthesis . Our data suggested that the catabolism of Met was clearly ↓ during the first 120 days of lactation; whereas, the biosynthesis of Lys was ↑ 2 weeks before lactation and at 240 vs. -30d when milk yield had dropped. Lys biosynthesis had an evident ↓ at the onset of lactation. Data seemed to indicate that mammary gland spared Met for protein synthesis, which is supported by it being one of the AA with lowest concentrations in blood but one with the highest extraction ratios by the mammary gland . Regarding Lys, our data suggest that mammary tissue synthesizes this AA from oxoglutarate when the rate of milk protein synthesis is low (e.g., in late lactation) but preferentially utilizes preformed Lys when the rate of milk protein synthesis is high (e.g., peak lactation).

Metabolism of other AA also was affected, particularly for seleno-amino acids at 120 vs. -30d, as well as Arg and Pro metabolism and Tyr metabolism at 240 vs. -30d. Furthermore, data revealed a significant ↑ of glutathione metabolism within the Small Molecular Biochemistry category in IPA functions during the first 30 days of lactation, and between 30 and 60d as shown by IPA canonical pathways and GO analysis when analyzed across consecutive time points. Our data seem to support a role for glutathione in mammary gland probably to provide AA as proposed previously .

When significantly affected, the post-translational modification of proteins tended to be ↓ and followed the general trend for protein synthesis. However, several functions within post-translational modification were affected including glycosylation at 1 (↓) and 60 (↑) vs. -30d, hydroxylation at 1d (↑), and ↑ phosphorylation by kinases at 15, 60, and 120 vs. -30d). Others (i.e., farnesylation, prenylation, ribosylation, and methylation) were ↓ during lactation. The glycosylation of proteins is recognized to be important in mammary tissue , whereas the ↑ in phosphorylation by kinases would likely be important for caseins . However, very few casein kinases were affected and most were ⇓.

Regulatory points of milk protein synthesis could not be clearly discerned from the current analysis, probably due to the lack of mammary-specific annotation as pointed out previously . The ↑ of insulin receptor signaling during the entire lactation, although statistically significant only at 30 vs. -30d, was associated with the induction of protein synthesis through mTOR. The importance of insulin for milk protein synthesis had been proposed previously and was recently confirmed in bovine mammary tissue explants . The mTOR signaling pathway appeared to be important also for other pathways such as PI3K/AKT signaling. Our data indicated that this pathway was ↓ during lactation except for the induction of mTOR signaling.

Other relevant metabolism. Additional metabolic functions are denoted in IPA analysis as *small molecular biochemistry*. Briefly, data from both IPA and GO analyses indicated ↑ of inositol phosphate (a second messenger molecule) metabolism before parturition, ↓ of nitrite accumulation and synthesis of prostaglandin during transition and at the end of lactation. Data also provided evidence of synthesis of hormones (particularly progesterone and testosterone) at 1 vs. -30d, between 60 and 120d and between 240 and 300d.

The importance of extracellular matrix-related synthesis was highlighted by canonical pathway analysis. This analysis uncovered a significant enrichment of glycosphingolipid biosynthesis, particularly of ganglioseries and globoseries, which were highly affected and ↓ at 60 and 120 vs. -30d and keratan sulfate biosynthesis which was highly affected between 1 to 120 vs. -30d. Less significant was chondroitin sulfate biosynthesis at 15, 60, and 120 vs. -30d. Detailed analysis of those pathways indicated an ↑ of synthesis of keratan and chondroitin sulphate (CS) during the first month of lactation and a ↓ between 60 and 120 vs. -30d. This was particularly evident for CS-E and CS-D for chondroitin and of sulfotransferase for keratan sulphate. All these components are essential for the structure of the extracellular matrix and the complex interactions which regulate cell-to-cell signaling, coordination of stromal-epithelial development, and the interaction with extracellular signaling molecules . Although their quantity in milk is low, the process of glycosphingolipid synthesis is of particular interest because of the current commercial focus in the production of these bioactive molecules due to their probiotic role in the intestine . In addition, gangliosides have an important role in membrane function such as modulating enzyme properties, cell signaling, cell adhesion, protein sorting, and formation of caveolae . The globosides are present in bovine milk but at low concentrations . A recent characterization of bovine milk oligosaccharides in colostrum and milk revealed a large decrease in concentration during the transition from synthesis of colostrum to mature milk as well as during the progression of lactation . Our data agree with those findings and suggest that the reduction of bovine milk oligosaccharides is driven by transcriptional adaptations, particularly of the biosynthesis of glycosphingolipids.

Transport**.** The *molecular transport* category was largely ↑ between -15 to 240 vs. -30d, with a nearly continuous ↑ during the first month of lactation. This finding is in line with previous results in peripartum mammary gland . This result is not surprising given that mammary is an active exocrine gland during lactation. Detailed analysis revealed that lactating mammary tissue transports a wide array of constituents with protein localization, uptake and intracellular transport of lipid and ions being the most important. Export of nucleotides from the nucleus (i.e., RNA) was ↓ during the whole lactation except between 120 and 240d when it was ↑. This trend supports the slight ↓ in transcription during lactation and the overall ↑ between 120 and 240d as shown by number of transcripts and reported in more details below (gene expression section).

Protein localization was ↑ at 1 and ↓ at 120 vs. -30d and transport of proteins was not significant during the first month of lactation. However, transport of proteins was significantly affected and primarily ↓ at 60 and 300 vs. -30d, between -15 and 15d, and between 120 and 240d. The fact that secretion of protein was not among the highly-significant functions and mostly ↓ was unexpected considering the large amount of proteins being secreted by this organ during lactation (>1 kg/day). However, it is important to note that protein in mammary tissue also can be secreted via vesicle-mediated transport (see below). Another puzzling observation was the ↑ in protein exocytosis and the high enrichment of protein trafficking at 2 weeks before parturition.

The transport of ions, particularly organic cation (↑ during the whole lactation), Ca^2+^ (highly-enriched during lactation), and Cu^2+^ (↑ at the end of lactation) were highly- enriched in IPA but not in GO analysis. Transport in GO appeared as highly-enriched at several consecutive time point comparisons. This category included inorganic ion transport (phosphate) which was ↓ between 1 and 15d and, among the ones enriched in separate analysis of ⇑ and ⇓, ↑ inorganic ion transport between 60 and 120d, ↑ ATP synthesis-coupled electron transport at -15 and 300 vs. -30d, ↑ phosphate transport at 1 and 240 and ↓ at 15 vs. -30d, and ↑ sulfate transport at 1 and 30 vs. -30d. The canonical pathway analysis revealed a significant enrichment of calcium signaling at 15 vs. -30d, between 15 and 60d and between 240 and 300d. The importance of calcium homeostasis for milk synthesis is well-established . The importance of phosphate transport for the formation, mainly associated with calcium, of casein micelles also is well-established . Interestingly, even though ion transport was apparently highly-enriched in GO analysis of ⇑ DEG, none of the specific ion transport systems appeared significantly enriched.

Among the highly-enriched functions were vesicle-mediated transport, which was ↑ during the whole lactation and also endocytosis, which was ↑ between 1 and 15d and 120 and 240d. Endocytosis was ↓ at 300 vs. -30d. The importance of endocytosis was further highlighted by canonical pathway analysis where we observed a significant enrichment of caveolar-mediated endocytosis between 30 and 60d, clathrin-mediated endocytosis between -15 and 1d, and macropinocytosis between 15 and 30d. The importance of vesicle-mediated transport is likely related to the secretion of milk constituents which uses the Golgi route, milk fat route, and transcytosis . These include the main milk constituents (protein, fat, lactose, calcium, and citrate) and other important components of milk (e.g., transferrin) or colostrum (immunoglobulins).

Drug metabolism**.** The pathways of active and passive transport of drugs by mammary gland have been known for some time . The importance of xenobiotic transport in mammary tissue does not appear secondary as suggested by the expression of several drug transporter proteins in this organ . In our data, drug metabolism was significant in most of the comparisons, but in particular the transport of drugs was important and ↑ during the first 120 days of lactation relative to pregnancy. This likely ↑ in transport of drugs during lactation is not surprising. Most of the effects on drug transport by mammary tissue are due to the action of ATP-binding cassette (ABC) superfamily of transport proteins . The binding and production of hormones, another highly-enriched category in drug metabolism, particularly progesterone, was highly relevant in the results from the data mining analysis. The data indicated a consistent ↓ of progesterone binding during the whole lactation, consistent with previous findings in mice, which is the likely reason for the lack of inhibitory effects on milk synthesis by this hormone when lactation is established (reviewed in ). In contrast, the binding of estrogen was increased during lactation with a peak between 60 and 120 vs. -30d mostly driven by estrogen receptor α (*ESR1*) which had an increase with a peak at 240d (also measured by qPCR ). The large increase in estrogen receptor was coupled with a marked decrease in progesterone receptor activity particularly at 1 vs. -30d and at 300 vs. -30d. The increase in estrogen receptor and its binding capacity in late lactation could indicate an inhibitory effect of estrogen on milk synthesis as well as a likely effect on inducing progressive involution , which might have overridden the inhibitory effect of progesterone. However, estrogen also might have a positive effect on protein synthesis potentially via the Akt/Protein Kinase B Pathway .

Estrogen appears to be essential for pregnancy-induced tertiary branching and proliferation and maintenance of differentiating alveolar cells in mice . The importance of estrogen in mammary gland development has been recently studied using microarrays in heifers, where it appeared to stimulate epithelial cell proliferation . Those data together with the ↑ in epithelial differentiation and exocrine gland development at 240 vs. -30d, which coincided with peak of *ESR1* expression, allowed us to speculate a possible role of estrogen in mammary development during late lactation. In addition, data indicated that mammary tissue might have produced (and eventually released) progesterone at the end of lactation, which appears to support previous findings regarding a possible endocrinological role of the mammary gland .

## 3.4. Mammary cell development during end of pregnancy and lactation

Besides regulation of metabolic pathways, many other cellular functions were highly affected both at the end of pregnancy and during lactation. The IPA and, partly, GO analyses associated particular functions or biological processes to specific cell types or tissues. It is becoming increasingly evident that the same proteins can play different roles in diverse organisms, cell types, or tissues in the same organism. This is the case, for example, of gene/protein isoforms . Therefore, to undertake a completely unsupervised analysis and to account for this observation, we detail our findings based on results from both bioinformatics tools recognizing that genes important in one tissue also can be important for the same function in other tissues.

Cell cycle and cell death**.** These were among the most significantly-affected functions in IPA, with a greater enrichment during the first month of lactation for both functions but with cell death being the most affected overall. Measurements of bovine mammary parenchymal tissue apoptosis and proliferation at both the end of pregnancy and lactation have been performed . However, previous work did not separate apoptosis/proliferation among cell types composing the parenchymal tissue. Our functional analyses, as previously mentioned, allowed us to separate between tissue/cell types but the overall functional analysis should be comparable to previous work because it considered the overall effect of multiple cell types on the function. Data from previous work indicated that apoptosis was higher at 48 days prior to parturition, ↓ at 2 weeks prior to parturition, ↑ 2 weeks after parturition where it reached a peak, and decreased thereafter with a numerical ↑ at mid-to-late lactation (90-120d) . Overall, IPA analysis did not completely support previous results. Cell death, appeared to be ↓ from -30 to 1d and tended to be ↑ at 30 vs. -30d. As previously suggested, the IPA analysis indicated an evident ↑ in cell death of leukocytes at 15 vs. -30d coupled with a possible ↑ of fibroblast cell death. In addition, data indicated an ↓ of cell death at 60 vs. -30d. The slight increase in apoptosis between 60 and 120d agreed with a previous report . However, our data indicated a likely ↑ of apoptosis at 240d and an evident ↑ at 300 vs. -30d. Previous analyses of cell proliferation and apoptosis did not include a time point beyond 120d.

The GO analysis results indicated a pivotal role of anti-apoptosis genes at the end of pregnancy and the beginning of lactation and pro-apoptosis genes between 60 and 120 vs. -30d of lactation. Overall GO results indicated ↓ in cell death between -30 and -15d, ↑ between -15 and 1d, ↓ again between 1 and 15d, and ↑ at 60 and 120d relative to -30d and ↓ between 120 and 240d. The overall data from IPA indicated that cell death was probably ↓ during lactation, except for being likely ↑ at 30 vs. -30d and evidently ↓ at 60 vs. -30d, and ↑ at the end of lactation. From this point of view, the GO data do not seem to support findings with IPA. The difference at 60 vs. -30d was striking, but details of GO results further indicated that the induction of apoptosis was highly ↑ at 60 and 120 vs. -30d, whereas induction of apoptosis by extracellular signals was ↑ at 15 and 300 vs. -30d. In addition, the GO analysis also indicated that the response to DNA fragmentation during apoptosis was ↑ during nearly the entire lactation. The disagreement between the two analyses prevents a clear conclusion, but both point out an ↑ in apoptosis at the end of lactation. In addition, the GO data seem more in line with previous reports . In fact, GO indicated an ↑ of apoptosis at 15 vs. -30d. In this regard, GO analysis suggested a larger degree of apoptosis at 1d compared with 15d of lactation. Furthermore, the IPA analysis indicated a probable ↑ in cell death at 30 vs. -30d. The detailed analysis in IPA highlighted a differential response among cell types, e.g., epithelial cells having ↑ apoptosis between 1 and 15d and neurons having ↓ apoptosis during the entire lactation. This last observation might explain the discrepancy between GO and IPA, the latter being more detailed on the type of cell/tissue.

In support of an ↑ of apoptosis during the first month of lactation was the fact that death receptor signaling was significantly enriched during the first 30 days of lactation. This, in fact, was the only significant pathway associated with cell death in IPA. Details of the pathway indicated a general ↑ in apoptosis through the death-inducing signaling complexes (i.e., FAS receptor) particularly at 1 and 30 vs. -30d. In addition, there was a ⇓ of TNF and DR receptors which are part of a second signaling complex in which most of the up-stream (i.e. *TNF*) and down-stream molecules were ⇑. This signaling complex can both regulate cell death and survival through NFκB . Evaluation of DEG within the pathway also indicated a possible ↓ of NFκB activity by means of ⇑ IκB, which may have prevented/reduced the induction of survival genes . For this reason the overall pathway was considered to be ↓.

Results of IPA analysis of the cell cycle, which counteracts cell death to maintain the number of cells composing the mammary gland, reflected almost perfectly the rates of proliferation measured previously in bovine mammary cells during pregnancy and lactation . Interestingly, there also was indication of an ↑ at -15 and a tendency for ↓ at 60 vs. -30d of epithelial cell cycle activity. GO analysis of ⇓ DEG uncovered a ↓ in mitosis at 15 and 300 vs. -30d. The ↓ in mitosis was also suggested by the IPA analysis of ⇓ DEG at 1, 15, 60, and 240 vs. -30d.

Several pathways were associated with cell cycle including aryl hydrocarbon receptor signaling, G1/S and G2/M DNA damage check point regulation, ceramide signaling, 14-3-3-mediated signaling, and the tight junction signaling. Among those highly-enriched were aryl hydrocarbon receptor signaling and tight junction signaling. Interestingly, all those pathways tended or were evidently ↓ across all comparisons. The aryl hydrocarbon receptor signaling highlighted a central role of v-myc myelocytomatosis viral oncogene homolog (*MYC*) in inducing cell cycle at -15d. In addition, the behavior of ceramide signaling, which was significant only between 30 and 60d, was suggestive of an increase in synthesis of ceramide from sphingolipids as previously discussed . Conversely, across all comparisons these results also were indicative of a complete shut-down of ceramide down-stream signaling for induction of apoptosis.

Overall, the analysis clearly pointed out that cell death and cell cycle are high-priority functions that are tightly controlled in bovine mammary tissue from the end of pregnancy through the end of lactation. These conclusions differ from the mouse mammary microarray data, where cell cycle or death functions were not among the most significant functions . In the functional analysis of mouse mammary tissue, only metabolic functions were significantly affected both at the end of pregnancy and during lactation, with the exception of cell and cytoskeleton organization and biogenesis . Although not fully comparable, our data are more in line with a recent mammary transcriptomics study where cell cycle was one of the most enriched functions among genes down-regulated 2-fold .

Cell growth and proliferation**.** This function was likely ↑ from -15 to 15 vs. -30d with a slight ↑ between 1 and 15d and a peak at 240d. This function combines cell cycle, cell growth, and colony formation of cells; thus, it cannot be fully compared to previous cell cycle and proliferation studies on bovine mammary cell proliferation . In the IPA analysis the importance of this function was evident for smooth muscle cells, which in mammary tissue can be part of the endothelial and “basket” compartments, and also for the immune cells. Epithelial cell proliferation and growth also were affected with a ↓ between -15 and 15d but a likely ↑ between 15 and 30d. The GO analysis highlighted a general ↑ of proliferation at 240 and 300 vs. -30 d.

Few pathways related to growth and proliferation were significantly affected. Among those, PI3K/AKT signaling was the only significantly affected at each time point relative to -30d and was significant during the first 120 days of lactation with a peak in enrichment between -15 and 1d and also between 120 and 300d. In the comparisons relative to previous time points, erythropoietin, FGF, and PDGF signaling pathways were significant between 30 and 60d and they were ↓ or likely ↓. PI3K/AKT signaling was the most significantly-enriched function in mouse mammary tissue between pregnancy and involution, with a large ↑ in lactation and a ↓ at involution . In our study, the pathway appeared to be mostly ↓ during lactation with a slight ↑ at the end of lactation (between 120 and 240d). These data highlighted that activation of AKT signaling in bovine mammary tissue is not essential for lactation. In addition, they underscored the large species difference in mammary transcriptomics adaptations for lactation . Our data, characterized by a minor enrichment and likely ↓ of pathways associated with growth and proliferation, highlights a minor importance of those pathways during lactation.

The large ↑ of cell cycle and ↓ of apoptosis uncovered from the combined analyses of IPA and GO results clearly indicated a net increase in the amount of cells at -15d. For other time points there was no full agreement between the two bioinformatics tools, with IPA indicating a balance between gain and loss of cells during the first 120d of lactation and an increase in cell loss at the end of lactation; whereas, GO confirmed the decrease of cell death at -15 vs. -30d and indicated a decrease in number of cells between 60 and 120 vs. -30d but did not confirm the increase in cell loss at the end of lactation. In a recent study, , proliferation was one of the most enriched functions among genes ⇓ 2-fold between end of pregnancy and beginning of lactation, allowing the conclusion that proliferation was significantly decreased in this time frame. Our data, support the same conclusions, especially based on data dealing with DNA replication and chromosome organization (see below).

DNA replication, recombination, and repair**.** This function was strikingly ↓ between 1 to 120 vs. -30d, and particularly DNA replication, which seemed to support the ↓ of cell cycle during lactation. The data indicated a ↓ availability of DNA for transcription (i.e., DNA relaxation) from 1 to 120 vs. -30d coupled with an ↑ of chromatin remodeling between 120 and 240d, both of which suggested switching of cell cycling and transcriptional regulation. this last observation goes along with the large number of DEG between 120 and 240d. The time frame between -15 and 1d was characterized by a large ↓ of DNA replication and relaxation, which might indicate a large ↓ in cell cycle (as suggested above). It also could indicate a ↓ in availability of DNA for transcription, which might partly explain the decrease in number of DEG found between 1 and 30d. The apparent ↓ in transcription at 30 vs. -30d seemed to have been induced by an ↑ in DNA condensation . The ↓ of DNA replication and chromatin remodeling also was reported by Funicane et al. as being one of the most relevant functions enriched among ⇓ DEG by 2-fold between end of pregnancy and early lactation.

The above observations together with data from Funicane et al. , led us to speculate that there is an epigenetic mechanism of transcriptional control characterized by modification of chromatin structure at the onset of lactation. This effect probably occurred before the last month prior to parturition because there was no evidence of DNA structural modification observed during the last month of pregnancy. Such a mechanism probably allowed for the large increase in number of DEG at the onset of lactation (1d), after which the mammary tissue had to maintain chromatin structure very stable until 120d essentially permitting continued physical access to promoter regions of specific genes essential for lactation. In the case of other genes, however, the access to promoter regions was prevented or became more difficult, i.e., genes were silenced. At later stages of lactation, between 120 and 240d, there was a change in DNA structure which could have allowed the tissue to enter a new physiological phase, e.g., gradual decrease in milk yield coupled with the onset of mammary restructuring. Interestingly, a strong relationship between replication and transcription has been suggested previously, both of which might share the chromosome remodeling machinery .

The GO analysis indicated an ↑ of DNA catabolism from -15 to 240 vs. -30d (except 1 and 30d), mostly related to DNA fragmentation during apoptosis. Additionally, GO analysis suggested ↓ of DNA unwinding at 60 and 120 vs. -30d (but also at 1 and 15d in ⇓DEG). The DNA dependent transcription was ↑ between 1 and 30d but strongly ↓ between -15 and 1d and largely ↑ between 120 and 240d (GO analysis of separate ⇑ and ⇓ DEG). Furthermore, GO analysis revealed a ↓ of DNA replication at 300 vs. -30d, a ↓ of DNA packaging and repair at 1, 60, and 120 vs. -30d with an ↑ between 15 and 30d. Overall, GO analysis supports the conclusion drawn from IPA analysis highlighting a general shut-down of DNA modification for replication and, probably, transcription.

Gene expression**.** The process of transcription can be regulated by the physical structure of the chromatin but the activation and deactivation of genes is under strict regulation by transcription factors. IPA analysis indicated a general ↓ of *gene expression* regulation by transcription factors from the onset through the end of lactation, particularly during the first 60 days of lactation relative to pregnancy. However, from the comparison of consecutive time points it was apparent that there was a large ↑ in expression between 60 and 120d and between 240 and 300d.

Detailed analysis indicated that several transcription factor binding sites were turned on and off during the course of lactation. Among the most enriched were the activation of NFAT (Nuclear factor of activated T-cells), likely↑ at -15 and 300 vs. -30d and ↓ during high-rates of milk production. This gene is associated with immune cells and has an apparent pro-apoptotic effect ; the expression of the E2F binding site, which controls expression of several genes involved in both cell cycle and apoptosis , was evidently ↑ at -15 and its activation was likely ↓ at 15, 60, and 120 vs. -30d; the p53 response element, which has a known pro-apoptotic role in mammary gland , was strongly ↓ at 1, 60, and 120 vs. -30d; and the C/EBP binding site (CCAAT/enhancer binding protein), with a crucial role in the differentiation of many cell types , which was likely ↓ at 15d, enriched at 30d, and likely ↑ at 300 vs. -30d.

The repression of Gal4p binding site (largely ↓ from 15 to 120 vs. -30d, except 30d) was highly-enriched in IPA across all time comparisons. This function encompasses 4 genes (*CBX1*, *3*, and *5*, and *DNMT1*) that have been well-studied in yeast, and whose cooperative activation is crucial in unicellular organisms for gene silencing through epigenetic regulation (mostly DNA methylation), which also seems to work in mammals . In the context of the present study, data strongly suggested a reduction of Gal4p repression, i.e., gene expression did not provide evidence of repression of DNA transcription by methylation due to the cooperative interaction of the aforementioned genes.

The overall ↓ in gene expression revealed by IPA was only partly evidenced by the number of differentially expressed transcripts calculated. In fact, we observed only a slightly larger number of ⇓ DEG compared to ⇑ DEG. In addition, we previously demonstrated that the total amount of RNA in these tissue samples increased during lactation likely due to few transcripts whose abundance becomes predominant in mammary tissue . This last observation also is supported by the ca. 10% of ⇑ DEG with >1-fold increase compared with -30d . The lack of repression of Gal4p uncovered in our analysis deserves further investigation because it appeared to be in some way associated with lactation. Several additional binding sites appeared to be ↑ or ↓ at the various time points vs. -30d.

In GO analysis, transcription was not among the most highly affected functions at all time points relative to -30d. However, categories enriched and in general ↓ during lactation included RNA metabolism. Transcription was, however, highly enriched between consecutive time points. GO uncovered a ↓ of transcription regulation at 1 vs. -30d, with a slightly greater number of genes involved in positive than negative regulation. Regulation of RNA metabolism also was ↓. GO analysis indicated a general ↓ of histone deacetylase during lactation with ↓ transcription particularly between -15 and 1d. However, general transcription was strongly ↑ between 1 and 15d and between 120 and 240d. These findings by GO are only weakly supported by the calculated number of DEG, where we observed a slighter greater number of ⇓ transcripts compared with ⇑ between -15 and 1d, and vice versa between 120 and 240d; however, between 1 and 15d the number of DEG decreased and there was no greater number of ⇑ compared with ⇓ DEG.

Among others, it was interesting to observe in GO analysis the ↓ in STAT nuclear translocation between -15 and 1 and ↑ between 120 and 240d. The STAT proteins, and particularly STAT5, have been considered to play a pivotal role in controlling expression of milk proteins . The ↓ in STAT nuclear translocation when milk casein expression increased dramatically does not support such a role. In addition, none of the STAT5 transcripts were significantly affected in our experiment, which supports previous findings . However, our data cannot definitively deny a pivotal role of this transcription factor in bovine mammary because its activity is mostly controlled via phosphorylation/dephosphorylation. A more detailed analysis in expression of genes involved in STAT5 signaling seems to indicate a minor role . The TGFβ receptor signaling pathway also was ↓ between -15 and 1d and ↑ between 120 and 240d. The TGFβ signaling through Smad is known to play a pivotal role in the cell cycle and might help to explain the reduced cell cycle activity between -15 and 1d.

The findings of a likely ↓ of gene expression together with a general ↓ of DNA and RNA metabolism and protein synthesis during lactation were all unexpected findings. In fact, lactating bovine mammary gland produces a large amount of protein and its total RNA increases dramatically compared with the non-lactating stage. These data strongly indicated that the mammary gland during lactation concentrates all its resources for synthesis of fewer proteins, i.e., on the one hand there is a decrease of the protein synthesis machinery available for translation coupled with a large increase in abundance of transcripts coding for relatively few proteins that are essential for milk synthesis itself or major components of the milk (e.g., α-lactalbumin, caseins, fatty acid binding proteins, stearoyl-CoA desaturase). This scenario suggests that the very large increase in expression of milk-related proteins is essential to provide a substantial competitive advantage relative to other types of proteins. In the end, it seems that the mammary gland favors such a mechanism instead of simply decreasing the abundance of other transcripts by a large margin. The proposed scenario is supported by the relatively low number of ⇓ DEG with expression >1-fold and by the demonstrated long half-life of casein mRNA .

Cellular assembly and organization**.** This function was amidst the most affected across lactation relative to pregnancy both in IPA and GO. Data interpretation from IPA analysis suggested a likely increase in organization of mammary tissue at 1 and 30d, a strong reorganization at the end of lactation, and an evident ↓ both at 15, 60, and 120 vs. -30d. This global function pertains to several cellular structures. The most evidently-affected during stages of lactation were the cytoskeleton, with organization of filaments being the most important (↓ from -15 to 60, but strongly ↑ at the end of lactation relative to -30d); formation of organelles, mainly peroxisomes (↓ from -15 to 240 vs. -30d and likely ↑ at 300d), lysosomes (largely ↓ from 15 to 120 vs. -30d, with likely ↑ activity at -15d through transport sorting of endosomes), and mitochondrial membranes (likely↑ at 15 and 30 vs. -30d and evident ↓ in mitochondria membrane fusion from 15 to 120 vs. -30d but an ↑ in mitochondrial membrane distribution at 15 vs. -30d); chromatin components and binding (strongly ↓ from 60 to 240 but likely↑ at 1 vs. -30d); vesicle formation and trafficking (likely↑ at 1 and 60 but ↑ at 300 vs. -30d); and quantity of centrosome and structures for cell division (important at 1, but ↓ at 15, 60, and 240 and likely↑ at 120 vs.-30d). In the comparison between consecutive time points, cell assembly and organization was consistently enriched particularly between 1 and 15d due to cytoskeleton rearrangement and endocytotic vesicle formation.

The GO analysis uncovered several biological processes which can be considered in the category of cellular organization and biogenesis. Relative to -30d, analysis revealed that this category is of central importance and was mainly ↓. However, analysis across consecutive time points indicated that cell organization and biogenesis was not among the most affected functions except between 1 and 15d, when there was a large re-organization of cytoskeleton and extracellular matrix. It was apparent from results that organization and biogenesis of organelles played a role, even though only mitochondria and lysosomes were substantially affected. The cytoskeleton organization and biogenesis was ↓ at the onset of lactation and progressively ↑ between -15 and 15d and at the end of lactation. Between -15 and 15d the substantial degree of cytoskeleton remodeling was probably coupled with ↑ in vesicle-mediated transport. The extracellular matrix and regulation of cell shape also were affected.

Results from GO cellular components revealed that the extracellular region components were of importance from 15 to 30 vs. -30d and at the end of lactation, when the extracellular matrix with formation of anchoring collagen became more important. Between -15 and 1d, focal adhesion was a highly-affected function together with a ↓ in plasma projection, assembly of ribosomes, and quantity of adherent junctions. The membrane became a prominent structure among ⇑ DEG at 15d, but the plasma membrane was more central at 240 vs. -30d. At 15 vs. -30d, the formation of apical tight junctions appeared evident, whereas the basal membrane was less enriched relative to the apical. However, analysis indicated that the basal membrane became more enriched at the end of lactation. Interestingly, between 120 and 240d, there was an evidently large reorganization of the whole cell including ↑ biogenesis of lysosomes, fusion of endocytic vesicles, and transport of Golgi vesicles.

Among specific organelles, none was important in GO Cellular components, except the ↑ in Golgi apparatus vesicle network between 1 and 15d; however, the separate analysis of ⇑ and ⇓ DEG allowed to uncover a significant effect on the endoplasmic reticulum (ER) constituents particularly integral to ER membrane and nuclear envelope-ER network during the whole lactation relative to -30d. The importance of the Golgi was apparently ↑ between -15 and 1d and between 1 and 15d, when the vesicle transport from Golgi was more important.

Analysis via IPA did not reveal significant canonical pathways associated with cell organization and biogenesis; however, two pathways could potentially be associated with these functions, namely tight junction signaling, which is important for regulation of cell cycle, cytosketetal organization, and barrier function, and regulation of actin-based motility by Rho with a role in regulation of cell polarity , which is essential for secretory processes in the mammary gland . Both were highly affected between 1 and 300d vs. -30d and also between 240 and 300d. Detailed analysis of the pathways among DEG relative to -30d indicated a more important biological role during lactation for junctional adhesion molecules (JAM) compared with claudins or occludins. This is supported by ⇑ of JAM genes and ⇓ of claudins downstream genes which regulate cytoskeletal organization; however, among 5 differentially expressed claudin genes only 2 were ⇓. The regulation of actin-based motility by Rho, which seems to be essential for the regulation of epithelial cell polarity , motility and phagocytosis, were significantly affected and ↑ at 240 vs. -30d and between 30 and 60d. However, it was ↓ between 240 and 300d.

The data uncovered a large degree of organization of mammary tissue cells before lactation, during the first month of lactation, and when lactation declined. Those data suggested that the cellular organization of the mammary gland has to be set before the beginning of copious milk secretion (or reaching peak milk production). At least from our data, mammary gland did not seem to undergo tissue organization during maximal milk synthesis, which is similar to findings in the mouse . Its re-organization, however, might play a role during the decrease of milk secretion.

The marked enrichment of ER and Golgi components in DEG between -15 and 1d was noteworthy as it revealed that most of those genes are involved in transport. This was reflected by the large enrichment of the ER-Golgi intermediate compartment. The lack of significant enrichment of ER and Golgi apparatus at all time points from 15 to 120d relative to -30d (when milk production was maximal) was striking. However, the ER was significantly enriched among ⇑ DEG during lactation relative to -30d. In addition, the genes coding for Golgi components were mostly ⇑ between -15 and 15d while components of ER membrane were ⇑ between -15 and 1d. Those results suggest a possible role of the Golgi in the progression from colostrum, which is the predominant mammary secretion at 1d, to mature milk, which is secreted after the first week of lactation. In addition, the data highlighted a crucial role of ER membrane components in the synthesis of milk components.

Cell-to-cell signaling and interaction**.** The importance of *cell-to-cell signaling and interaction* (which includes cell adhesion) and *cell signaling* was evident in both IPA and GO analyses. In summary, the results suggested that cellular signaling pathways are important for mammary tissue function during the first month and at the end of lactation, with kinase signaling (except MAPK) mostly ↓ during lactation. However, the ↑ of plasma membrane receptor binding at 1, 15,240, and 300 vs. -30d underscored the capacity of this tissue to allow for signaling mechanisms to take place. Epithelial cell adhesion at the onset and at the end of lactation was ↑, whereas appeared likely ↑ during the first mo of lactation. Interestingly, between 60 and 120d, cell signaling was not significantly enriched. Overall, the data suggest that mammary tissue is responsive both during the first 2 wk of lactation and at the end of lactation, whereas, it is less responsive once lactation is established. Those data partly support previous results from mouse mammary tissue during lactation, which suggested that the mammary cells segregate themselves from the outside environment in order to produce copious milk . Our results seem to suggest that the mammary gland is prone to external communication and cell-to-cell communication (e.g., epithelial adhesion) at the onset of lactation and during the rapid increase in milk synthesis; when lactation is established, mammary tissue appears less responsive to external stimuli but becomes again responsive as towards the end of lactation.

The importance of cell signaling also was evidenced by the canonical pathway analysis where the number of significantly-enriched signaling pathways was greater than metabolic pathways, i.e., 23 vs. 16 in the comparison of all time points vs. -30d and 46 vs. 19 at consecutive time points. Among signaling pathways, several were not directly related to particular tissues or functions and will be discussed in this section. The remaining signaling pathways are discussed in relation to specific functions. It is important to highlight that even though functional analysis supported a previously-suggested decrease in cell communication in murine mammary tissue during lactation , the signaling pathways largely affected in bovine mammary were not similar. In fact, the integrin signaling pathway was not significantly affected in our study, whereas in mouse it was among the most significantly-enriched .

The calcium signaling pathway was significantly enriched and probably ↓ at 15 vs. -30d as well as in subsequent comparisons between 15 and 60d and between 240 and 300d. The importance of calcium also was highlighted by functional analysis in IPA. Calcium in mammary tissue is extremely important both as a macroelement in milk and as a constituent of casein micelles, as well as being an intracellular signaling molecule . Judging by ⇓ of calcium down-stream signaling molecules, our data indicated that the mammary gland, despite its large uptake of calcium, experienced a ↓ of calcium signaling capacity during lactation. This effect might have been specific to control excessive cell proliferation stimulated by calcium, which was partly restored at the end of lactation. It is also possible that mammary tissue reduced its sensitivity to the multitude of effects triggered by calcium signaling.

The ERK (extracellular-regulated kinase)/MAPK (mitogen activated protein kinase) signaling pathway, involved in control of a broad range of intracellular functions , was substantially affected and likely ↓ at the onset of lactation (1 vs. -30d), between -15 and 1d, at 60 vs. -30d, and was ↑ when milk synthesis declined (between 120 and 240d). There is no apparent explanation for ↓ of this pathway during lactation. However, as previously reported, it controls a myriad of molecular events but its functions for the most part remain enigmatic to date .

Significantly-enriched pathways that affect several functions in many tissues such as muscle development were α-adrenergic signaling (↓ at 1 vs. -30d due to ⇓ of downstream genes despite ⇑ of adrenergic α2A receptor), cAMP-mediated signaling (↓ at 1 vs. -30d, except the signaling through Gi-coupled receptor, mainly involved in chemotaxis), and p38 MAPK signaling (↓ at 300 vs. -30d). Other affected pathways included ephrin receptor (↓ at 1 and 60 vs. -30d and between -15 and 1d and 120 and 240d), which controls cell behavior related to attraction/repulsion, adhesion/de-adhesion of neurons, angiogenesis, and synaptic plasticity; Hypoxia signaling (↓ between -15 and 1d); NFκB signaling (↑ between 15 and 30d, mostly due to the ↑ phosphorylation of IKβ); and ↓ erythropoietin signaling between 30 and 60d.

The enrichment of many signaling pathways in our data highlighted the importance of such level of regulation in the mammary gland, which has long been considered an organ under strict control by several hormones or other signaling molecules . In this regard, our data suggested that signaling through phosphorylation was ↓ or it was of lesser importance in mammary tissue during lactation than during the non-lactating period. In addition, an overview of the most affected signaling pathways suggested that in general there was a ↓ of intracellular signaling transmission.

## 3.5. General physiological system development and functional adaptation of mammary gland from pregnancy to lactation

Cell/tissue morphology and development. Overall results from the IPA and GO analyses highlighted a central role for cellular development and morphology throughout lactation. IPA analysis uncovered a general ↓ of cellular morphogenesis and development during lactation and ↑ before parturition (-15d, only in IPA analysis) and at the point that milk synthesis and secretion declined (240 vs. -30d). Analysis between consecutive time points highlighted a general ↓ of those functions between -15 and 1d and an (gradual) ↑ from 1 to 240d. Those findings suggested a sudden “termination” of morphogenesis at the onset of lactation, a process that was then reinitiated when milk yield declined. In addition, cellular morphogenesis was mostly ↓ during maximal milk production. The GO analysis supported the findings from IPA and underscored the ↓ of development and differentiation at 15 vs. -30d and between -15 and 1d, with increases between 1 and 15d and at 240 vs. -30d, except for the cellular development which, opposite to IPA, was ↓ at 240 vs. -30d. The tissue morphology and development together with organ development and morphology were generally ↓ during the first 2 mo of lactation and largely ↑ at 240 vs. -30d. Interesting in this regard was the indication by GO of ↑ in cell polarity between 15 and 1d and ↓ between 120 and 240d.

Overall, our results indicated that morphogenesis occurred prior to the onset of lactation, was discontinued during maximal milk production, and became again important when milk synthesis declined. This indicated that cell/tissue structure had to be set before lactation began and had to remain stable during milk synthesis. More importantly, our data strongly suggest that morphogenesis is involved in controlling milk synthesis. During pregnancy, the mammary gland of all species experiences alterations in morphogenesis and development. Both functions typically experience a ↓ at pregnancy, although in some species except the cow these processes continue during early lactation . The apparent ↑ in cell morphology between consecutive time points seems to support previous findings of changes in cellular morphology during the first period of lactation . Data also suggested that morphological and developmental changes were of different magnitude across distinct cell types. From IPA and GO analyses, it was observed that the cell types with greater changes were immune, fibroblast, epithelial, endothelial, neurons, and muscle. Particular functions of those cell types are discussed below.

The immune system**.** The immune system was denoted in IPA by *immune and lymphatic system development and function*, *immune response*, and partly by *Cellular Movement* and *cellular compromise* and *haematological system development and function*. The immune system was significantly affected during the various stages of lactation with a general nadir of significance (or non-significance) at -15d, a peak at 30d, a minimum during lactation at 120d, and a rebound (sometimes with a peak) at the end of lactation. In particular, considering the comparisons relative to -30d in IPA, data indicated an overall ↑ in immune system development between -15 to 30d and at 240 and 300d, with a ↓ at 60 and 120d. Immune response was likely ↑ during the first month of lactation and in late lactation; whereas, it was likely ↓ at the end of lactation. Considering the comparisons between subsequent time points, the development of the immune system was ↑ between 1 and 15d and between 60 and 120d and was ↓ between 120 and 240d. This last observation seems to contradict data vs. -30d, but details clearly highlighted different sub-functions among the two, with the comparison of 240 vs. -30d enriched by differentiation and between 120 and 240d enriched by adhesion of lymphocytes.

Before lactation, data suggested an ↑ of differentiation and development of immune cells, particularly granulocytes, including macrophages and B-lymphocytes but no inflammatory activity of the immune system was apparent. Judging by cellular movement, with selection between immune cells types, the immune cells appeared to be recruited at 1 vs. -30d and between -15 and 1d. Despite this apparent stimulation of the immune system and the ↑ in cell response to perturbation of plasma membrane, the data also suggested a ↓ of inflammatory response during this time frame, which also was supported by the likely ↓ in oxidative stress response. Between 1 and 15d, data clearly indicated an ↑ in immune cell recruitment but a ↓ in inflammatory response. At 15 vs. -30d, data suggested a likely ↑ of leukocyte proliferation, in particular lymphocytes, but a ↓ in differentiation and an ↑ in neutrophil polarization. Between 15 and 30d, there was an evident ↑ in inflammatory response which was substantiated when comparing responses at 30 with -30d. At 30d, despite an ↑ in recruitment of macrophages and monocytes, their infiltration into mammary tissue appeared to be ↓. In addition, data also suggested an ↑ of growth of memory T lymphocytes and likely an ↑ in immune cell death. At 60 and 120 vs. -30d, the immune system was likely shut-down, except at 120d when data suggested a likely ↑ in leukocyte growth. Between 120 and 240d there was an evident ↓ in immune system development and inflammatory activity. Despite this, at 240 vs. -30d data indicated an ↑ of recruitment of immune cells, particularly granulocytes (phagocytes and macrophages) and T-lymphocytes, with a likely ↑ in immune response. Between 240 and 300d there was indication of an ↑ in immune cell infiltration (eosinophils and lymphocytes). Compared with -30d at 300d there was likely an ↑ in movement and activation of leukocytes probably as consequence of ↑ secretion of cytokines. Data did not indicate an ↑ of inflammatory response but rather a likely ↓ judging by the ↓ of the antiviral response and selection of T lymphocytes.

The GO analysis revealed a central role of the immune system during lactation. Relative to -30d, the response to wounding was ↑ at 15d and at the end of lactation. In the latter, inflammatory response also was ↑ despite ↓ of defense response. Comparison of subsequent time points uncovered a substantial ↑ in inflammatory response between 15 and 30d and at the end of lactation. Furthermore, inflammatory response was enriched in ⇑ DEG at 60 and 120d. Antigen presentation was evidently ↓ during the whole lactation (except 30d) and particularly due to ⇓ of MHC class I and II protein complexes. However, this function was highly enriched in ⇑ DEG between 120 and 240d. Overall, the defense response appeared ↓ at the end of lactation, particularly vs. -30d. Other functions such as antiviral response, also were uncovered by GO with an interesting ↓ of viral replication between -15 and 1d a time when colostrum starts to be synthesized and, interestingly, the ↑ in response to viruses between 15 and 30d and 120 and 240d.

Many pathways within IPA are related to general immune system activity and immune response. Among those, antigen presentation, IL-4 signaling, interferon signaling, and LPS/IL-1 mediated inhibition of RXR function were significantly enriched in at the least 1 time point vs. -30d. Interestingly, however, these pathways were nearly all ↓ during lactation, except for IL-4 signaling. An additional large number of pathways (18) plus 3 related to stress (ER stress pathway, NRF-2-mediated oxidative stress, and hypoxia signaling) were enriched in the analysis covering subsequent time points. A common feature among most of those pathways was their predominance between 15 and 30d and between 30 and 60d. Despite the apparent importance of immune-related canonical pathways between 15 and 30d, the detailed analysis of the pathways did not indicate an overwhelming ↑ in immune-related activities. Among those ↑ or likely ↑ pathways were some related to interferon (which also included the activation of interferon-regulatory factor by cytosolic pattern recognition, and a role of protein kinase-regulated [PKR] in interferon induction of antiviral response), acute-phase response, NFκB, IL-10 signaling, and pattern recognition receptors for bacteria and viruses. Other pathways appeared to be ↓. Overall, data indicated a general ↓ of immune-related pathways between 30 and 60d.

In modern high-producing cows, the importance of the immune response in mammary gland is pivotal due to the need for preventing/fighting mastitis . Our data highlighted several unique aspects of mammary tissue:

- Despite the fact that many of the genes could be associated to the presence of immune cells forming part of the mammary gland compartment, the large enrichment of immune-related functions together with the predominance of epithelial cells in our core biopsies indicated that the mammary gland is actively involved in the immune system and supports the suggestion of the mammary gland being an evolutionary product of the innate immune system
- The mammary gland is highly active in recruiting and preparing immune cells, which our data showed began quite abruptly at day 1 of lactation but without an overt inflammatory response (except between 15 and 30d). The infiltration of immune cells, particularly macrophages, into mammary tissue is essential for the initiation and resolution of an inflammatory response . Our data suggested increased accumulation of macrophages or immune cells in general. The functional data was partly supported by specific immune cell markers. Those data were indicative of a more pronounced increase in macrophage infiltration between 1 through 60d when it reached a peak followed by a decrease afterwards. The increase in number of macrophages in mammary tissue during the surge in milk production is similar to data reported for the rat . In contrast, neutrophil infiltration seemed to increase smoothly through the end of lactation;
- Although none of the cows used in this study had signs of clinical or sub-clinical mastitis, data between 15 and 30d were suggestive of an inflammatory response. However, during that time-frame we observed the most pronounced increase in milk yield coupled with the lowest number of DEG relative to -30d; furthermore, the number of DEG between 15 and 30d was minimal. No explanation is readily available for this observed phenomenon;
- The significant enrichment of functions related to viral genome replication and life, particularly ↓ between -15 and 1d, indicated that the mammary gland shuts-down mechanisms to control potential viral infections during the formation of colostrum but it becomes an important process during the decrease in milk production. Even though the capacity of the mammary gland to “produce” viruses has been known for a long time , the biological meaning of this is not apparent;
- A striking finding was the consistent ↓ of the major histocompatibility complex (MHC), which is similar to microarray data of mouse mammary gland during lactation despite the fact that the MHC class II in mouse is not expressed in epithelial cells as it is in bovine . The MHC is present in all nucleated cells and it is the mechanism whereby all cells participate in the immune defense, presenting antigens from bacteria and virus to CD8+ T-lymphocytes . An association between Class I alleles and mastitis traits has been found . The mRNA expression of bovine mammary MHC components is increased due to intramammary challenge with *S. Uberis* strain O140J , probably due to the epithelial response, as previously demonstrated *in vitro* . Those data confirm the lack of mastitis in the mammary tissue used in the present investigation. In addition, the ↓ of MHC during lactation in mammary tissue goes along with the low immune activation inferred from IPA results. The reason for the ↓ of this function is not readily apparent because the mammary gland is an easily accessible site of bacterial entry which in turn requires a rapid immune response. Under such scenario we propose two hypotheses: 1) genetic selection of cattle for high milk production, which has brought about an increase in mastitis incidence , might have inadvertently reduced the expression of the MHC during lactation resulting in lower sensitivity to bacteria and a consequent decrease in energy expenditure for immune responses; 2) the MHC is a vesicle-dependent process which uses ER-Golgi networks as do milk components; thus, the MHC can be considered a competitor of the vesicle-transport system. The MHC inhibition can be considered a way for the mammocytes to spare resources. The first hypothesis, however, does not explain the reduction of MHC in mammary mouse which has not been selected for lactation, while the second hypothesis seems more holistic.

Cardiovascular system development and function**.** In IPA the cardiovascular system was affected only during lactation, except at 60d compared with -30d; whereas, in GO it was highly enriched between -15 and 15d but without a clear direction. Relative to -30d, it was characterized by a ↓ of endothelial morphology at 1d, a likely ↑ in endothelial remodeling and angiogenesis at 15d, a likely ↓ of binding of endothelial cells at 30 and 120d coupled with an effect on endothelial morphology at 30d and ↑ of area of blood cells at 120d, an ↑ in angiogenesis at 240d, and a likely ↓ in vascular remodeling at 300d. Analysis across consecutive time points revealed a likely ↓ in blood vessel development and morphology (angiogenesis) between -15 and 1d, a large ↑ in angiogenesis but ↓ in blood vessel morphology between 1 and 15d, a likely ↑ in angiogenesis and ↑ attachment of endothelial cells between 15 and 30d, a ↓ in morphology of endothelial cells between 30 and 60d, an ↑ of angiogenesis between 60 and 120d, and a likely ↓ of endothelial morphogenesis and binding of high endothelial postcapillary venules between 120 and 240d.

Few canonical pathways in IPA were related to cardiovascular system. β-adrenergic signaling was highly-enriched at 1 vs. -30d. Even though this pathway is important for the modulation of cardiac function, the detailed analysis indicated that the receptor for norepinephrine was ⇑ but its downstream signaling targets were ⇓. The ⇓ in downstream signaling targets might have ↓ the sensitivity towards blockage of milk ejection by epinephrine . In this regard, it was interesting that the same pathway was ↑ between -30 and -15d even though it was not significantly-enriched. The biological importance of this pathway in mammary tissue is not apparent; however, most of the adrenergic receptors were ⇑ during the whole lactation, which might indicate a particular sensitivity of the mammary gland to this type of hormone, particularly for the control of blood flow within endothelial cells . Another pathway related to blood flow control is hypoxia signaling in cardiovascular system , which was significantly enriched and ↓ between -15 and 1d. Although it was not significant, it was ↓ during the whole lactation suggesting a likely low state of hypoxia within the mammary gland.

Connective tissue development and function**.** The connective tissue was negatively affected during the whole lactation relative to -30d, particularly due to a ↓ quantity of fibroblasts (i.e., cell division/proliferation) except at 15 vs. -30d when there was an evident ↑ in colony formation of fibroblasts. In the comparison between consecutive time points we observed a large ↓ of proliferation, growth, cycling, development, and survival of fibroblasts between -15 and 1d; a ↓ of differentiation, adhesion, branching, chemotaxis and cycling, and an ↑ of formation of pericytes between 1 and 15d, and a ↓ in adhesion and cycling of fibroblasts between 240 and 300d. .

The GO analysis uncovered an overall ↑ in the response to wounding particularly at 15, 240, and 300 vs. -30d, which can be considered a task accomplished by fibroblasts . Analysis of ⇑ DEG suggested that this function was ↑ during the whole lactation and there was an ↑ in the response to wounding between 1 and 15d. In addition, GO analysis indicated an ↑ of fibroblast growth factor receptor binding between -15 and 1d.

The importance of connective tissue for the mammary gland has not been fully elucidated, but appears to be important for the regulation of mammary epithelial growth and ductal development/elongation (e.g. ). However, fibroblasts, which compose the connective tissue and become the supporting structure for other cells, also have an important effect on the function of epithelia related to the formation of a proper 3-dimensional structure . Our data suggested that the activity of the fibroblasts is generally ↓ during lactation. This observation seems to corroborate the overall ↓ of cell/tissue remodeling reported above.

Nervous system development and function**.** The nervous system was significantly affected during the first phase of lactation and particularly at 15 vs. -30d and between 15 and 30d. The most affected functions in the nervous system category were cell death, which appeared consistently ↓ between -15 to 120 vs. -30d, it returned to balance at 240 vs. -30d and it became non-significant at 300 vs. -30d. Also the morphogenesis and proliferation of the nervous system was ↓ for nearly the whole lactation compared with pregnancy. The nervous system, however, was highly active before parturition, at 15, 30, 120, and, partly, at 240 vs. -30d. Furthermore, data suggested a likely ↑ of innervation of the organ by patterning of axons at 1d and a ↓ at 240 vs. -30d. The GO analysis uncovered a ↓ in neuron differentiation between 1 to 120 relative to -30d, a ↓ in neuron development and morphogenesis between 15 to 60 relative to -30d and an ↑ in neurophysiological process between 15 to 120 relative to -30d. Similar results were suggested by consecutive time-point analysis. There were several significant pathways in IPA related to nervous system function. When considering comparisons vs. -30d, dopamine receptor signaling and axonal guidance signaling were significantly enriched at 1 and 30d and at 1d (mostly ↓), respectively. When consecutive time point comparisons were considered, neuregulin signaling (↓), synaptic long-term potentiation (↔) and synaptic long-term depression (↑) were all significantly affected between 15 and 30d.

Overall, the data clearly indicated that the nervous system was developed before parturition and then during lactation its development and cell turnover were inhibited but instead activity of these processes in mammary was highly active. Those data are in agreement with the overall response in mammary gland development discussed in previous sections, i.e., higher rates before parturition and rather quiescent during lactation. The importance of the nervous system in the mammary gland is well-known mainly for its role in milk ejection but also for its likely control of blood flow both of which are essential for lactation.

Endocrine system development and function**.** A potential endocrine role of mammary tissue was highlighted at several points during lactation and, based on the interpretation of the IPA, was generally ↓ during the whole lactation with no significant differences between consecutive time points except a noteworthy ↑ between 120 and 240d. The details in IPA revealed a ↓ of hormone binding, particularly progesterone, between -15 and 1d, and at 1, 60, 120, and 300 vs. -30d. Additional responses included a possible ↑ of estrogen secretion into milk (via ABCG2) between 30 and 60d, a likely ↓ in progesterone synthesis between 60 and 240d, a likely ↑ of estrogen binding at 60 and 120 vs. -30d, a ↓ of glucocorticoid synthesis at 15 and 240 vs. -30d, a likely ↑ in quantity of acinar gland cells from 1 to 120 vs. -30d, a large ↑ in hormone binding (particularly β-estradiol), a ↓ in synthesis of 4-androstene-3-17-dione between 120 and 240d, and a large ↓ in progesterone and estrogen binding between 240 and 300d. The GO analysis revealed a ↓ in hormone biosynthesis (mainly C21-steroids) between -30 and -15d, an ↑ of estrogen biosynthesis between -15 and 1d, a ↓ of insulin secretion between 1 and 15d, a ↓ of steroid hormone receptor signaling pathway between 15 and 30d, an ↑ of the progesterone signaling pathway between 120 and 240d, and a ↓ in response to hormone at 300 vs. -30d.

In IPA, C21-steroid hormone metabolism was significantly affected at 240 vs. -30d and details of the pathway indicated a clear ↑ of synthesis of progesterone and 17α-hydroxicortexone. Even though not always significant, there was a progressive increase between -30 to 240d of this pathway towards the synthesis of those hormones. In addition, we observed an increase in significance of androgen and estrogen metabolism which reached a P-value = 0.059 at 240 vs. -30d. The details of this pathway indicated an increase in the synthesis of 17β-estradiol from 17α-hydroxycortexone.

Overall, those data further indicated a strong effect of steroid hormones on mammary tissue, as also pointed out above and in a previous review . In addition, the results highlighted a possible role of ABCG2 for transport of estrogen into milk . The active transport of estrogen in milk has been clearly shown and partly explains the high relative mRNA abundance of *ABCG2* expression in bovine mammary tissue . The C21-steroid hormone metabolism pathway indicated that mammary gland can synthesize progesterone and estradiol particularly at the end of lactation. Those data might explain the increase in milk estradiol and its higher concentration compared with plasma estradiol . This latter observation, however, does not exclude a possible endocrine role of mammary tissue, as previously pointed out , which besides estrogen and testosterone also seems to produce cortisol (Suppl. Excel file 3). These data together with the significant increases in expression of several cytokines (e.g. TNFα), hormones (e.g., prolactin), and signaling molecules during lactation, support the view of a local control of mammary development and function . In this regard, a recent study reported a numerical or significant decrease in expression of several cytokines between the month prior to pregnancy to early lactation . Several of those previous data contrast with our microarray data, particularly TNFα.

**TABLE R4**. Tabulated results from Ingenuity Pathway Analysis® (IPA) at **-15 vs. -30d**. Reported are the functions with an Exact Fisher test P-value ≤ 0.05 sorted by decrease in significance. The category denotes the main functional category assigned by IPA. The functional annotation is derived by the “effect on function” in IPA. In parenthesis are reported the number of DEG for each specific function and the arrows denote the overall effect on the function inferred by the gene annotation using IPA ( = highly activated;  = activated;  = tend to be activated;  = highly inhibited;  = inhibited;  = tend to be inhibited). For detailed explanation see Additional file 2. Some annotations on functions are common between categories and were not repeated and the overall effect takes into consideration common functions among categories. Some categories do not make biological sense based on mammary gland features (see Additional file 2 for details explanation). For those two reasons the sum of the numbers in parenthesis very often does not correspond with the total DEG in the left column (DEG)

| **Category** | **Function Annotation** | **DEG** |
| --- | --- | --- |
| Protein Synthesis | Synthesis of protein (52, **⇑⇑**) | 75 **⇑⇑** |
| Cell Death | Cell death of neurons (28, **↓**); regeneration of cells (5, **↓**); apoptosis of peripheral blood lymphocytes (3, **↑**) | 71 **↓** |
| Cell funct & maint**^1^** | Exocytosis of proteins (4, **⇑**); discharge of neurons (2, **⇑**) | 8 **⇑** |
| Molecular transport**^2^** | Accumulation of inositol phosphate (8, **⇑**), prostaglandin (3, **⇓⇓**), nitrite (3, **⇓⇓**); transport of organic cation (5, **⇑⇑**), drugs (3, **↑**), taurocholic acid (3, **↓**); uptake of taurocholic acid (3, **⇓⇓**); clearance of cholesterol (2, **⇑**); export glutathione (2, **⇑**); quantity of arachidonoylglycerol (2, **⇑**); translocation of calcium (2, **⇓**); | 34 **⇑** |
| Cellular development | Development of leukocytes (20, **⇑**); differentiation of leukocytes (12, ⇔); pro-B lymphocytes (3, **⇑**); maturation of hematopoietic cells (7, **⇑**); colony formation of granulocytes (4, **⇑⇑**) | 51 **⇑** |
| Heam Syst D & F | Differentiation of leukocytes (12, ⇔); development of macrophages (7, **↑**); maturation of hematopoietic cells (6, **⇑⇑**); colony formation of granulocytes (4, **⇑⇑**); differentiation of pro-B lymphocytes (3, **⇑**); morphology of leukocytes (3, **⇑**); detachment of granulocytes (2, **⇑**); | 31 **⇑** |
| Cell cycle | Delay in cell division (13, **⇑**); G0 phase (10, **⇑⇑**); G0/G1 phase transition (10, **⇑⇑**); entry in mitosis (4, ⇔); biogenesis of mitotic spindle (4, **↑**); entry in cell division process of epithelial cells (3, **⇑⇑**); polyploidization of blood cells (3, **⇑⇑**); cycle progression muscle cell (2, **⇑**) | 38 **⇑⇑** |
| Carbohydrate metabolism | Hydrolysis of carbohydrates (17, **⇑⇑**), phosphatidic acid (13, **⇑⇑**), phopshionositide (6, **⇑**), ADP-D-ribose (2, **⇑**), phosphatidylglyceroids (2, **⇑**), phosphatidylserine (2, **⇑**); accumulation of carbohydrates (14, **⇑**), glycosides (4, **⇓**), inositol phosphate (8, **⇑⇑**) | 27**⇑⇑** |
| Cell growth & prolif | Outgrowth of cells (4, **⇑**); colony formation of stem cells (3, **↑**); | 25 **↑** |
| Cellular assembly and organization | Bundling of filaments (6, **⇓**); quantity of gap junctions (4, ⇔), secretory granules (4, **↑**); aggregation membrane raft (3, **⇑**); trafficking vesicles (3, **↑**); anchoring microtubules (2, **⇓**); formation peroxisomes (2, **⇓**); movement Golgi (2, **⇑**); transport sorting endosomes (2, **⇑**) | 30 ⇔ |
| Conn tissue D & F | Contraction of fibroblasts (2, **⇓**); conversion of fibroblasts (2, **⇑**) | 13 ⇔ |
| DNA rep rec & repair | Cross-link repair of DNA (3, **↑**) | 7 **↑** |
| Imm Syst D & F | Energy of T-lymphocytes (3, **⇓**) segregation of T and B lymphocytes (2, **⇓**) | 31 **⇑** |
| Lipid metabolism | Hydrolysis of lipid (17, **⇑⇑**); accumulation of eicosaenoic (3, **⇓**); | 32 **⇑** |
| Organismal function | Locomotion (15, **⇓**); recovery (4, **⇓**) | 19 **⇓** |
| AA metabolism | Prenylation AA (3, **↓**); farnesylation AA (2, **⇓**); secretion of thyroxin (2, **⇑**); transport of GABA (2, **⇓**) | 9 **↓** |
| Cell morphology | Contraction of cells (3, **⇓**); morphology of leukocytes (3, **↑**); frequency of micronuclei (2, **⇑**); mineralization of leukocytes (2, **⇑**); ruffling of epithelial cells (2, **⇑**) | 20 **↑** |
| Cell-to-cell sign & inter | Activation of stem cells (2, **⇑**); detachment of neutrophil (2, **⇑**); hyperexitation of neurons (2, **⇓**) | 11 **↑** |
| Gene expression | Transcription of Sp1 binding site (7, ⇔); activation p of NFAT RE (5, **↑**), acute phase response (3, **↓**), inverted CCAAT box (2, **⇓**), p73 binding site (2, **⇓**), initiation of transcription (5, **↑**); expression of E2F binding site (4, **⇑**), STAT3 binding site (2, **⇑**); transcription of STAT3/STAT6 binding site (2, **⇓**) | 27 **↑** |
| Endocrine system D & F | Biosynthesis of androgen (2, ⇔); quantity of corticotroph cells (2, **↓**) | 4 **↓** |
| Immune response | TH1 immune response (3, ⇔); stimulation of colony-forming units granulocytes (2, **⇑**) | 7 |
| Nervous Sys D & F | Nerological process (14, **↑**); long term depression (3, **↓**); discharge of neurons (2, **⇑**); | 31 **↑** |
| Organ morphology | Transformation of mammary gland (2, **⇑**) | 17 **⇑** |
| Post-translational mod | Prenylation of amino acids (3,** ↓**); farnesylation of amino acids (2, **⇓**); incorporation of protein (2, ⇔); ribosylation of proteins (2, **↓**) | 7 **↓** |
| Sk & musc sys D & F | Morphology of muscle (3, **↑**); skeletal and muscular process of fibroblast cells (3, **↑**); cell cycle progression of muscle cells (2, ⇔) | 12 **↑** |
| Tissue morphology | Quantity of colony forming unit-megakaryocites (3, **↓**), erythrocytes (3, **↓**); quantity of neuroblasts (2, **⇑**); separation of cells (2, **↑**) | 20 **↑** |
| Cell movement | Haptotaxis (5, **↓**); segregation of B lymphocytes (2, ⇔) | 9 **↓** |
| Hair & Skin D & F | Entry into S phase of epithelial cells (4, **⇑⇑**); arrest in G2 phase of epidermal cells (2, ⇔); ruffling of epithelial cells (2, **⇑**) | 11 **↑** |
| Nucleic acid metabolism | Binding of GDP (2, **⇑**); hydrolysis of ADP-D-ribose (2, **⇑**); incorporation of uridine (2, ⇔) | 6 **↑** |
| Cell signaling^3^ | Translocation of calcium (2, **⇓**) | 2 **⇓** |

^1^Also includes protein trafficking; ^2^Also includes small molecular biochemistry, drug metabolism, and part of lipid metabolism; ^3^Also includes Vitamin and Mineral metabolism. Other functions were not included because they did not make biological sense with respect to the mammary gland

**TABLE R5**. Tabulated results from Ingenuity Pathway Analysis® (IPA) at **1 vs. -30d**. Reported are the functions with an Exact Fisher test P-value ≤ 0.05 sorted by decrease in significance. The category denotes the main functional category assigned by IPA. The functional annotation was derived by the “effect on function” in IPA. In parenthesis are reported the number of DEG for each specific function and the arrows denote the overall effect on the function inferred by the gene annotation using IPA (⇑⇑ = highly activated; ⇑ = activated; 🡩 = tend to be activated; ⇓⇓ = highly inhibited; ⇓ = inhibited; 🡫 = tend to be inhibited). For detailed explanation see Additional file 2. Some annotations on functions are common between categories and were not repeated and the overall effect takes into consideration common functions among categories. Some categories do not make biological sense based on mammary gland features (see Additional file 2 for details explanation). For those two reasons the sum of the numbers in parenthesis very often does not correspond with the total DEG in the left column (DEG)

| **Category** | **Function Annotation** | **DEG** |
| --- | --- | --- |
| Cell Death | Fibroblasts (91, **↑**); neuron  (67, **↓**); muscle cell (43, **↓**); granule cells (15, **⇓**); stem cells (13, **↑**); vascular smooth muscle cells (10, **⇓**); helper T lymphocytes (4, **⇑**) | 616 **↓** |
| Tissue morphology | Quantity of fibroblasts (8, **⇓**), parenchymal cells (5, **↑**), brown adipose tissue (4, **⇓⇓**), progenitor cells (4, **⇓⇓**), acinar grand cells (3,**↑**); conduction of nerves (4, **⇓**); thickness of mucosa (3, **↓**) | 46 **⇓** |
| Nucl acid metab | Metabolization of nucleic acid component or derivative (15, **↓**) | 15 **↓** |
| Cell cycle | Mitosis (69, **⇓**); entry into cell stage (39, **↑**), fibroblasts (10, **↓**); G2 phase (38, **↓**), fibroblasts (8, **↓**); senescence (26, **↑**); cytokinesis (25, **⇓**); cell stage of fibroblasts (22, **↓**); modification of chromosome components (22, **↓**); interphase of fibroblasts (20, **↓**); arrest in cell division of fibroblasts (16, **⇓**); S phase of fibroblasts (14, **↓**); arrest in interphase of fibroblasts (10, **⇓**); arrest in G1 phase leukocytes (9, ⇔); aneuploidy (7, **⇑**); cell division process of adipocytes (7, **↑**), chromosomes (6, **⇑**), brown adipocytes (4, **⇑**); delay in M phase (6, **⇓**); G2/M phase transition of fibroblasts (6, ⇔); DNA damage checkpoint (4, **⇓**); M phase fibroblasts (4, ⇔); re-entry into cell stage fibroblasts (4, **⇓**); delay in G2/M phase transition (3, **↓**); mitogenesis brown adipocytes (3, **↑**); senescence stage (3, **⇑**) | 183 **↓** |
| Hair & Skin D & F | Differentiation epidermal cells (27, **↑**), keratanocytes (18, **↑**); development epidermis (22, **⇑**); re-epitheliazation of wound (4, **↑**); laxity of skin (3, **↑**) | 51 **↑** |
| Organ develop | Organogenesis (136, ⇔); morphogenesis of organ (30, **↓**); | 162 **↓** |
| Cell assembly & organ | Transport of vesicles (43, **↑**); binding of cellular membrane (13,** ⇓** ); elongation of neurites (13, ⇔); bundling of filaments (12, ⇔); organization of microtubules (10, **↓**); quantity of centrosome (10, ⇔); maintenance of telomeres (8, **⇓⇓**); formation of micronuclei (4, ⇔); perturbation of plasma membrane (4, **⇑⇑**); quantity of fibrils (4, **↑**); structure of chromatin (3, **↑**) | 123 **↑** |
| Cell-to-cell sign & interact | Activation of cells (144, **↑**); binding of epithelial cells (9, **↑**); adhesion of mast cells (7, **⇓⇓**); stimulation of neutrophils (5, **⇑⇑**); binding of neurons (4, ⇔); hyperactivation of T lymphocytes (4, **⇓**); recruitment of dendridic cells (4, **⇑**); adhesion of PBMC cells (3, **↑**) | 164 **↑** |
| Cell morphology | Transformation of cells (87, **↓**), fibroblasts (71, **↓**); morphology of fibroblasts (21, ⇔); autophagy (16, ⇔); blebbing (13, **⇑**); elongation of neurites (13, ⇔), blood cells (4, **⇑**); morphogenesis of cells (11, **↓**), endothelial cells (6, **↑**), epithelial cells (4, ⇔); polarization of Th1 lymphocytes (6, **⇑**); patterning of axons (4, **↑**); cell flattening of macrophages (3, **⇑**) | 151 **↓** |
| Cellular compromise | Oxidative stress response of cells (12, **↓**); instability of chromosomes (10, **↓**); condensation of nucleus (7, **↑**); degradation of cells (5, **⇓**); permeability of mitochondrial membrane (4, **⇑**); perturbation of plasma membrane (4, **⇑⇑**) | 33 ⇔ |
| Nerv Sys D & F | Binding of neurons (4, ⇔); innervations of organ (4, **⇑**); patterning of axon (4, **↑**); accumulation of microglia (3, **↓**) | 35 ⇔ |
| Protein synthesis | Stabilization of proteins (10, **⇓**); biosynthesis of cytokines (4, **⇑**) | 14 **↓** |
| Drug metabolism | Transport drug (6, **↑**); binding progesterone (5, **⇓⇓**); quantity folic acid (4, **⇓**); synthesis glutathione (4, **⇑**); transport glutathione (4, ⇔) | 18 **↑** |
| Molecular transport | Transport lipid (26, **↑**), heavy metal (9, ⇔), organic cation (7, **⇑⇑**), organic anion (5, **↓**); localization of mRNA (5, **⇓**); accumulation of nitrite (4, **⇓**); excretion of sterol (4, ⇔); localization lipid (4, **⇑**); release of neurotransmitter (4, **⇑⇑**); clearance of cholesterol (3, **⇑**); distribution of ganglioside (3, **⇑**) | 64 **⇑** |
| Cell movement | Migration epithelial cell (25, **↑**), phagocyte (7, **⇑**), antigen presenting cell (5, **⇑**), macrophage (5, **⇑**); rolling eosinophil (4, ⇔) | 64 **⇑** |
| DNA rep rec rep | DNA cleavage (12,⇔); DNA ligation (5,**⇓**); delay DNA fragmentation (4,**⇑⇑**); chromosome recombination (4,**↓**); DNA relaxation (4,**⇓⇓**) | 36 ⇔ |
| Hematolog Sys D & F | Maturation of myeloid cells (8, **⇑**), granulocytes (5, **↓**); survival of macrophages (7, **⇓**); polarization of Th1 lymphocytes (6, **⇑**); development of plasmacytoid dendridic cells (3, **↓**) | 39 **↑** |
| Organ morphol | Morphogenesis in organ (30,** ↓** ); healing of the organ (4, **⇓⇓**) | 30 **⇓** |
| Cell growt & prol | Proliferation (469, **↑**); growth (275, **↓**), fibroblasts (60, **↓**), mesenchymal cells (10, **↑**); colony formation (115, **↓**), fibroblasts (27, **↑**); | 572 **↑** |
| Cell development | Development fibroblasts (80, **↓**), neurons (66, **⇓**); differentiation of epithelial cells (50, **↓**), epidermal cells (27, **↑**);maturation of bone marrow cells (13, **↑**); lifespan of cells (10, **⇓**); self-renewal stem cells (9, ⇔); morphogenesis of endothelial cells (8, **↑**) | 196 **↓** |
| Carbohydrate metabolism | Accumulation of carbohydrates (32, **⇑**); exposure phosphatidylserine (7, **⇑**);synthesis of phopshatidylcholine (7, **⇑**); accumulation of glycosides (5, **⇓**); elimination of glucose (3, **⇓**) | 46 **⇑** |
| Cell signaling | Inactivation of MAP kinase (7, **↓**) | 7 **↓** |
| Lipid metabolism | Transport lipid (26, **↑**), exposure of phospholipids (8, **⇑**); binding of progesterone (5, **⇓⇓**); synthesis of glycosylceramide (5, **↑**); distribution of glycolipid (4,** ⇑**), ganglioside (3, **⇑⇑**); excretion of sterol (4, **↑**); localization of lipid (4, **⇑**); clearance of cholesterol (3, **⇑⇑**) | 51 **↑** |
| Molecular bioch | Modification of AA (135, **↓**); glycosylation AA (10, **⇑**); biosynthesis of hormones (5, **⇑**), essential AA (4, ⇔); catabolism of Ser A (4, ⇔); hydrodylation of AA (4, **⇑**) | 212 ⇔ |
| Card syst D & F^1^ | Morphogenesis of endothelial cells (6, **⇓**) | 69 **⇓** |
| Gene expression | Activation of NFAT RE (9, **↓**), p53 RE (6, **⇓⇓**), MYB binding site (4, **⇓**); repression of Gal4p binding site (4, **⇓⇓**) | 28 **⇓** |
| Tissue develop | Development of extraembryonic tissues (13, **⇓**), ectoderm (5, **⇓**) | 34 **⇓** |
| Cell func & maint | Organization of microtubules (10, **⇓⇓**); maintenance of telomeres (8, **⇓⇓**) | 18 **⇓⇓** |
| Conn tissue D & F | Proliferation of fibroblasts (60, **↓**); growth of fibroblasts (60, ⇔); morphology of fibroblasts (21, ⇔); quantity of fibroblast (7, **⇓**); colony formation of fibroblasts (7, **⇓⇓**); quantity of brown adipose tissue (4, **⇓⇓**); branching of fibroblasts (3, ⇔); mitogenesis of brown adipocytes (3, ⇔); senescence stage of fibroblasts (3, **⇑**) | 122 **⇓** |
| RNA trafficking | Localization of RNA (5, **⇓**) | 5 **⇓** |
| End syst D & F | Binding of hormones (8, **⇓**), progesterone (5, **⇓⇓**); quantity of acinar gland cells (3, **↑**) | 11 **⇓** |
| Immune response | Inflammation response (5, **⇓**); | 25 **↑** |
| Imm & lymp Sys D & F | Adhesion of mast cells (7, **⇓⇓**), PBMC cells (3, **↑**); polarization of Th1 lymphocytes (6, **⇑**); stimulation of neutrophils (5, **⇑**); hyperactivation of T lymphocytes (4, ⇔); cell flattening of macrophages (3, **⇑⇑**); development of plasmacytoid precursor dendritic cells (3, **↓**) | 25 **↑** |
| Post-trans modif | Modification of amino acids (135, **↓**); glycosylation of amino acids (10, ⇔); hydroxylation of amino acids (4, **⇑**); ribosylation of amino acids (3, **⇓**) | 137 **↓** |
| Amino acid metabolism^2^ | Biosynthesis of essential amino acids (4, ⇔ [**↑ **branched and Met AA; **⇓** Cys]), sulfur amino acids (4, ⇔ [**↑ **sulfur AA]); catabolism of Ser family amino acid (4, ⇔); quantity of folic acid (4, **⇓**); | 146 **↓** |
| Organismal devel | Development (47, **⇓**) | 51 **⇓** |
| Organismal funct | Healing of organ (4, **⇓**) | 4 **⇓** |
| RNA post-transcr | Processing of tRNA (4, ⇔ [**⇑ **Ala and Ser tRNA; **⇓ **Phe tRNA) | 4 ⇔ |

^1^Many genes are involved in hearth development and were not take into consideration for the mining analysis. ^2^Includes also Vitamin and Mineral metabolism. Functions not present in the table but reported in Suppl. Excel file 3 are those that did not make biological sense with respect to the mammary gland.

**TABLE R6**. Tabulated results from Ingenuity Pathway Analysis® (IPA) at **15 vs. -30d**. Reported are the functions with an Exact Fisher test P-value ≤ 0.05 sorted by decrease in significance. The category denotes the main functional category assigned by IPA. The functional annotation was derived by the “effect on function” in IPA. In parenthesis are reported the number of DEG for each specific function and the arrows denote the overall effect on the function inferred by the gene annotation using IPA (⇑⇑ = highly activated; ⇑ = activated; 🡩 = tend to be activated; ⇓⇓ = highly inhibited; ⇓ = inhibited; 🡫 = tend to be inhibited). For detailed explanation see Additional file 2. Some annotations on functions are common between categories and were not repeated and the overall effect takes into consideration common functions among categories. Some categories do not make biological sense based on mammary gland features (see Additional file 2 for details explanation). For those two reasons the sum of the numbers in parenthesis very often does not correspond with the total DEG in the left column (DEG)

| **Category** | **Function Annotation** | **DEG** |
| --- | --- | --- |
| Cell cycle | Cell stage (160, **↓**), fibroblast cell lines (28, **⇓**); mitosis (59, **↓**), fibroblast cell lines (7, **⇓**); entry into cell division process of cells (46, **↑**); entry into cell stage (41, **↑**), fibroblasts (9, **↓**); entry into interphase of eukaryotic cells (30, **↑**); arrest in entry into S phase of eukaryotic cells (29, **↑**);delay in cell stage (23, **↑**); senescence (22, **↑**), connective tissue cells (10, **↓**); cytokinesis (20, **↓**); G2/M phase transition of eukaryotic cells (19, **↑**), connective tissue cells (6, **↑**); G0/G1 phase transition of cell lines (15, **↑**); S phase of fibroblasts (12, **↑**); arrest in cell stage of connective tissue cells (10, **↓**); arrest in interphase of connective tissue cells (10, **↑**); initiation of interphase of cell lines (8, **⇓**); delay in mitosis (8, **↓**); aneuploidy (8, **↑**), lymphatic system cell (3, **↑**); checkpoint control (7, **⇓**); initiation of cell stage of fibroblast cell lines (6, **↓**); arrest in G2 phase of fibroblasts (6, **↑**); cell division process of stem cells (6, **↑**), chromosomes (5, **↑**) | 173 **↓** |
| Cellular Growth and Proliferation | Proliferation of cells (379, **↑**), leukocytes (99, **↑**), vascular smooth muscle cells (14, **↑**), muscle cells (41, **↑**), parenchymal cells (17, **↓**); colony formation of connective tissue cells (10, **↑**); growth of eukaryotic cells (213, ⇔), vascular smooth muscle cells (6, **↑**), nervous tissue cell lines (3, **↓**); formation of cells (61, **↑**), leukocyte cell lines (3, **↑**); pluripotency of embryonic stem cells (3, **↑**) | 448 **↑** |
| Cellular Development | Developmental process of neurons (64, **↓**), endothelial cells (30, **↑**), vascular smooth muscle cells (8, **↑**), leukocyte cell lines (38, ⇔); differentiation of neurons (46, **⇓**), leukocyte cell lines (23, **↓**), myoblasts (17, **⇓**); initiation of differentiation of cells (3, **⇓**); maturation of red blood cells (8, **⇑⇑**); morphogenesis of epithelial cells (4, **↓**) | 178 **↓** |
| Cell Death | Apoptosis (381, **↓**), fibroblast cell lines (73, **↑**), neurons (55, **↓**), endothelial cells (25, **↓**), T cell blasts (5, **↓**), muscle cells (35, **↓**), helper T lymphocytes (3, **↑**), mammary epithelial cells (3, **⇓**); inhibition of apoptosis (49, **↓**); activation-induced cell death (19, **↓**), leukocytes (12, **↑**), T lymphocytes (10, **⇑**); necrosis of cell lines (11, **⇓**); survival of stem cells (10,**↑**); regeneration of axons (7, **↓**) | 449 **↓** |
| Skel muscle dev & func | Proliferation muscle (41,**↑**), vascular smooth muscle (14, **⇑**);differentiation myoblast (17, **⇓**); growth vascular smooth muscle cell (6, **⇑**); | 123 **↑** |
| Cell assembly and organization | Quantity of neurites (14, **⇓**); binding of cellular membrane (11, ⇔); elongation of neurites (11, **⇓⇓**); extension of axon (10, **⇓**); quantity of centrosome (10, **↓**); biogenesis of lysosome (7, **⇓⇓**), peroxisome (6, ⇔), mitochondria membrane (3, **↑**); rearrangement of filaments (7, **⇓⇓**); quantity of chromosome (5, **⇓**); accumulation fo vesicles (4, **⇓⇓**); fusion of mitochondria (4, **⇓⇓**); mitochondria distribution (3, **⇑**) | 85 **⇓** |
| Lipid metabolism | Quantity of lipid (76, **⇑**), fatty acids (27, **⇑**), glycolipid (16, **↑**), ganglioside (6, **↑**); synthesis of sphingolipid (13, **⇑**), phosphatidic acid (9, **⇑**); cerebroside (5, **⇑⇑**), glucocorticoids (4, **⇓⇓**), prostaglandin E2 (3, **⇓**) | 91 **⇑** |
| Small molecular biochemistry | Homeostasis cation (28, ⇔); production D-glucose (13, **⇑⇑**); metabolism phosphoric acid (8, **↑**); transport essential amino acid except Arg (3, **⇑⇑**), Arg (2, **⇓**); binding GDP (3, **↓**); incorporation aromatic amino acid (3, **⇓⇓**); metabolism adenine (3, **↓**); uptake of Asp (3, **⇑⇑**) | 136 **⇑** |
| Cardiov syst dev & func | Angiogenesis (80, ⇔); spreading of endothelial cells (6, **⇑**); morphology of endothelial cells (5, **⇑**); remodeling of vasculature (4, ⇔) | 111 **↑** |
| Conn Tiss Dev & Func | Colony formation of fibroblasts (22, **⇑**); quantity of fibroblasts (7, **↓**); quantity of white adipose tissue (4, **⇓**) | 58 **↑** |
| Tissue morphology | Mass of tissue (30,⇔); thickness of tissue (18, **↓**); quantity of parenchymal cells (5, **↓**), progenitor cells (4, **⇓⇓**), acinal cells (3, **↑**) | 60 ⇔ |
| Cell morphology | Transformation of fibroblasts (57, ⇔); length of plasma membrane projection (13, ⇔); elongation of neurites (11, **⇓⇓**); extension of axons (10, **↓**); cell spreading of endothelial cells (6, ⇔); morphology of endothelial cells (6, **⇑**); length of dendrites (4, **⇓**); morphogenesis of epithelial cells (4, **↓**); polarization of granulocytes (4, **⇑**); frequency of micronuclei (3, **↑**) | 94 ⇔ |
| Molecular transport | Quantity of calcium (76, ⇔), lipid (76, **↑**), fatty acid (27,** ↑**), glycolipid (16, **↑**), ganglioside (6, **⇑**), glycosylceramide (5, **⇑**), cerebroside (4, **↑**); release of calcium (36, **⇑**); transport of metal ion (9, **↓**), drug (5, **⇑**), essential amino acid (5, **↑**), glutathione (3, ⇔); accumulation of reactive oxygen species (8, ⇔); distribution of glycosphingolipid (4, **⇑**); depletion of calcium (3, **⇑**); uptake of Asp (3, **⇑**) | 163 **↑** |
| Nervous sys D & F | Differentiation of neurons (46, **⇓⇓**); neurological process (38, **⇑**); migration of neurons (32, **↓**); quantity of neurites (14, **⇓**); elongation of neurites (11, **⇓⇓**); length of neurites (11, ⇔); extension of axons (10, **⇓**); nociception (7, **↑**); regeneration of axons (7, **⇓**); accumulation of neuroglia (4, **↑**);retraction of dendrites (4, **⇑**); growth of neurons (3, **⇓**) | 129 **↓** |
| Tissue development | Survival of tissue (4, **⇓⇓**); outgrowth of tissue (3, **↓**) | 22 **⇓** |
| Immune Syst D & F | Proliferation of leukocytes (99, **↑**), lymphocytes (95, **↑**), T lymphocytes (78, ⇔); activation of leukocytes (57, ⇔); differentiation of leukocytes (23, **⇓**); size of lymphoid organ (14, **↓**); polarization of neutrophils (4, **⇑**); stasis of leukocytes (4, **↑**); activation of PBMC (3, **↑**); formation of leukocytes (3, **↑**) | 133 **↑** |
| Organ morphology | Morphology of the organ (24, **↓**) | 44 **↓** |
| Carbohydrate metab | Quantity of carbohydrates (59, **↑**); biosynthesis of carbohydrates (22, **⇑**); metabolic process of polysaccharides (20, **↓**); | 83 **↑** |
| Cellular movement | Movement (211, ⇔); migration (209, ⇔); | 283 ⇔ |
| Post-translational modif | Activation proteins (19, ⇔), protein kinase (14, **⇑**); incorporation proteins (3, **↓**); nitration proteins (3, **↑**); ribosylation proteins (3, **⇓**) | 38 **↑** |
| DNA repl rec & repair | Fragmentation of DNA (36, **↑**); checkpoint control (17, **⇓**); quantity of centrosome (10, ⇔); instability of genomes (6, **↑**); quantity of chromosomes (5, **⇓**); relaxation of DNA (4, **⇓**); loss of chromosomes (3, **⇓**) | 64 **↓** |
| Cellular compromise | Atrophy (10, **⇓⇓**); fragmentation of nucleus (10, **⇑**); condensation of nucleus (6, **⇑⇑**) | 28 **↑** |
| Cell signaling | Homeostasis of calcium (18, ⇔); activation of MAPKKK (3, **↑**); loading of GTP (3, **⇑**) | 100 **↑** |
| Cell funct & maint | Homeostasis of calcium (18, ⇔);stasis of leukocytes (3, **⇑**) | 22 ⇔ |
| Vitamin & Min Metab | Quantity of calcium (76, ⇔); release of calcium (36, **⇑**); depletion of calcium (3, **⇑**) | 96 **↑** |
| Hem Syst D & F | Proliferation of leukocytes (99, **↑**); T lymphocytes (78, ⇔); activation of leukocytes (57, ⇔); differentiation of leukocytes (23, **⇓**);maturation of red blood cells (8, **⇑⇑**); polarization of neutrophils (4, **↑**); | 131 **↑** |
| Gene expression | Initiation of transcription (15, **⇓**); expression of rRNA (6, **⇓⇓**), C/EBP binding site (4, **↓**); repression of Gal4p binding site (4, **⇓⇓**); activation of E2F (4, **↓**), C/EBP (4, **↓**), CACCC box (3, ⇔), CCAAT element (3, ⇔), progesterone response element (3, **↓**); binding of CREB/ATF binding site (3, **↓**) | 38 **⇓** |
| Immune response | Proliferation of leukocytes (99, **↑**); lymphocytes (95, **↑**), T lymphocytes (78,** ↑**); activation of mononuclear leukocytes (57, ⇔); cytotoxic reaction of cells (7, **↑**); activation of PBMC (3, **↑**) | 118 **↑** |
| Amino acid metabolism | Transport of essential amino acid (5, **↑**); incorporation of Phe (3, **↓**); uptake of Asp (3, **⇑**) | 10 **↑** |
| Cell-to-cell sign & inter | Association of plasma membrane (4, **⇑**) | 65 **↑** |
| Endocrine syst D & F | Biosynthesis of glucocorticoid (4,** ⇓⇓**); quantity of acinar gland cells (3, **↑**) | 10 **↓** |
| Nucleic acid metabolism | Binding of GDP (3, **↑**), GTP (3, **↑**); metabolism of adenine (3, **↓**) | 9 **↑** |
| Drug metabolism | Transport of drug (5, **↑**), glutathione (4, **↑**); biosynthesis of prostaglandin E2 (3, **↓**) | 9 **↑** |
| Free radical scavenging | Accumulation of reactive oxygen species (8, ⇔) | 8 ⇔ |
| Organismal develop | Development of vessel (80, ⇔); response of organism (43, **↑**); fibrillogenesis (4, **⇓**) | 122 ⇔ |
| Organ development | Morphogenesis of organ (24, **↓**) | 32 **↓** |
| Reprod sys D & F | Nursing (3, **↑**) | 13 **↑** |
| Protein synthesis | Incorporation of Phe (3, **↓**) | 3 **↓** |

**TABLE R7** . Tabulated results from Ingenuity Pathway Analysis® (IPA) at **30 vs. -30d**. Reported are the functions with an Exact Fisher test P-value ≤ 0.05 sorted by decrease in significance. The category denotes the main functional category assigned by IPA. The functional annotation is derived by the “effect on function” in IPA. In parenthesis are reported the number of DEG for each specific function and the arrows denote the overall effect on the function inferred by the gene annotation using IPA (⇑⇑ = highly activated; ⇑ = activated; 🡩 = tend to be activated; ⇓⇓ = highly inhibited; ⇓ = inhibited; 🡫 = tend to be inhibited). For detailed explanation see Additional file 2. Some annotations on functions are common between categories and were not repeated and the overall effect takes into consideration common functions among categories. Some categories do not make biological sense based on mammary gland features (see Additional file 2 for details explanation). For those two reasons the sum of the numbers in parenthesis very often does not correspond with the total DEG in the left column (DEG)

| **Category** | **Function Annotation** | **DEG** |
| --- | --- | --- |
| Cell Death | Neurons (58, **↓**); epithelial cell (28, **↑**); phagocyte (24, **↑**); antigen presenting cell (19, **↑**); macrophage (18, **↑**); neuroglia (11, **↓**); monocyte (5, **↓**); peripheral lymphocyte (4, **↑**); leukocytes(56, ⇔); sensory neuron (9, **↑**); cytotoxic T cell (4, **↓**); cell viability of epithelial cell (5, **⇓**); condensation (3, **↓**); pyknosis (3, **↑**); self-renewal of cell (8, ⇔); self-renewal of hematopoietic progenitor cell (4, **↓**) | 137 **↑** |
| Lipid metabolism | Accumulation lipid (27, **⇑**); quantity of phospholipid (25, **⇓**), glycosphingolipid (16, **⇑**), phosphatidylinositol (14, **↓**), phosphatidate (22, **↓**), phosphoinositide (12, **↓**), ceramide (12, **↑**), phosphatidylinositol trisP (9, ⇔), phosphatidylinositol diP (7, **⇓**), phosphatidylinositol diP (4, **↓**), ganglioside GM2 (3, **↑**), lactosylceramide (3, **↓**); exposure phospholipid (6, **↑**); esterification cholesterol (6, **↑**); distribution glycosphingolipid (4, **⇑**), glycolipid (4, **⇑**), ganglioside (3, **⇑**); synthesis glycosylceramide (4, **↓**); lipolysis triacylglycerol (3, **⇑⇑**); release glycosphingolipid (3, **⇑⇑**) | 65 **⇑** |
| Molecular transport | Calcium flux (32, **↑**); calcium influx (21, **↑**); quantity of nitric oxide (9, **⇑**), NADPH (3, **↓**); reactive oxygen species secretion (4, **⇑**), release of Glu (7, **↑**), superoxide (6, **↓**), acetylcholine (4, **↑**), glycosphingolipid (3, **⇑**); transport organic anion (4, **↑**), aromatic AA (3, **⇑**); uptake Ala (2, **⇑**) | 93 **⇑** |
| Molec biochem | Release of amino acids (9, **↑**); metabolism of glutathione (8, **↓**), production of D-glucose (9, **↑**); activation of JUN KK (3, **↓**) | 93 **↑** |
| Imm & Lymph Syst. D & F | Activation of, leukocytes (54, ⇔), lymphocytes (44, ⇔), natural killer cells (14, **⇑⇑**), mast cells (6, **↑**), T lymphocytes (4,** ⇓**); quantity of bone marrow cells (30, **↓**), megakaryocytes (7, ⇔), leukocyte (5, **↑**); differentiation of leukocyte (16, **⇓**); proliferation of macrophages (7, **↓**); expansion of memory T lymphocytes (5, **⇑**); growth of memory T lymphocytes (5, **⇑**); formation of megakaryocytes (3, **↑**); | 86 **↑** |
| Cell cycle | Arrest in cell stage of eukaryotic cells (50, **↓**), lymphocytes (5, **↓**); mitogenesis of leukocyte cell lines (7, **↑**) | 67 **↓** |
| Cell morphology**^1^** | Morphology (73, ⇔); contraction (15, **⇓⇓**); shape change blood cell (15, **⇑**); sprouding cell (9, **⇓⇓**); reorganization filament (6, **⇑**); polarization microtubule organizing center (5, **⇓⇓**); size endocrine cell (5, **↑**); cell flattening, elongation blood cell (3, **⇑**) | 126 ⇔ |
| Organis funct | Coagulation of bodily fluid (17, **⇑**); energy expenditure (8, **↑**) | 31 **⇑** |
| Cell assem & org | Organization of filaments (23, **↓**), actin filaments (14, **↓**), microtubules (9, **↑**), actin stress fiber (6, **↑**); formation of focal adhesions (21, **↑**); reorganization of filaments (6, **⇑**); disruption of plasma membrane (3, **↑**); hyperpolarization of mitochondria (3, **↑**); | 68 **↑** |
| Cell funct & main | Hyperpolarization (7, **↓**); localization of blood cells (5, **↓**), bone marrow cells (3, **↓**), lymphatic system cells (3, **↓**); maintenance of neurites (2, **⇓**) | 44 **↓** |
| Card sys D & F | Binding of endothelial cells (17, **↓**); morphology of blood vessel (8, ⇔) | 33 **↓** |
| Cell-to-cell sign & interaction | Activation of blood cells (64, **↑**), mesothelial cells (6, **↓**); attachment of cells (21, **⇓**); adhesion of epithelial cells (11, **↑**), muscle cells (6, **⇓**), smooth muscle cells (5, **⇓**); plasticity of synapse (11, **↑**); recruitment of macrophages (11, **⇑**), monocytes (6, **↑**); activation blood platelet (10, **⇑**); | 114 **↑** |
| Free radic scaveng | Production of superoxide (16, **⇑**); release of superoxide (6, **↓**); secretion of reactive oxygen species (4, **⇑⇑**) | 21 **⇑** |
| RNA p-trans mod | Modification of RNA fragment (4, **⇓⇓**) | 4 **⇓⇓** |
| Cell development | Maturation of blood cells (21, **⇑⇑**); developmental process (17, **↓**); differentiation of epidermal cells (16, **↑**), leukocytes (16, **↓**), epithelial cells (12, **↑**), erythrocytes (4, ⇔); developmental process of peripheral blood leukocytes (6, **⇑**), erythrocytes (4, **↓**), mononuclear cells (4, **⇑**); self-renewal of blood cells (5, **⇓**); lifespan of blood cells (4, ⇔); growth of memory T lymphocytes (3, **↑**); remodeling of neurons (3, **↑**); | 71 **↑** |
| Hem sys D & F | Infiltration of macrophages (14, ⇔), monocytes (6, **⇓**); mitogenesis of leukocytes (7, **↑**); flow of blood (6, **⇓**); mobilization of phagocytes (5, **⇑⇑**); survival of macrophages (5, **⇓⇓**); | 112 **↑** |
| Tissue morphology | Quantity hematopoietic cell (19, **↓**), parenchymal cell (4, ⇔), stromal cell (4, ⇔); density of cell (11, **↑**); morphology blood vessel (8, ⇔), capillary vessel (3, **↓**); | 62 **↓** |
| Organismal devel | Morphology of vessel (8, ⇔); epithelial-mesenchymal transformation (4, ⇔); development of bodily fluid (2, **↓**) | 17 ⇔ |
| Cellular movement | Localization of blood cells (5, **↓**); chemotaxis of monocyte-derived dendritic cells (3, **⇑**); extravasation of mononuclear cells (2, **⇓**) | 35 ⇔ |
| Immune response | Recruitment macrophage (11, **↑**), monocyte (6, **↑**); inflammation tissue (10, **↑**); generation B lymphocyte (4, ⇔); stimulation Th1 lymphocyte (2, **⇑**) | 74 **↑** |
| Hair & skin D & F | Differentiation of epidermal cells (16, **↑**); adhesion of epithelial cells (11, **↑**); cell viability of epithelial cells (5, **↓**) | 29 **↑** |
| Nucleic acid metab | Metabolism of nucleic acid components and derivatives (10, **↓**) | 14 **↓** |
| Nervous sys D & F | Neurogenesis (56, **⇓**); neurological process (30, **↑**); survival of sensor neurons (6, **⇓⇓**); neuroprotection of cells (5, **↓**); regeneration of nervous tissue (3, **↑**); initiation of outgrowth of neurites (2, **⇓**); maintenance of neurites (2, **⇓**); | 98 **⇓** |
| Cell growth & prolif | Formation of blood cells (11, ⇔); colony formation of erythroid cells (4, **↑**); formation of hematopoietic progenitor cells (4, **⇑**); generation of B lymphocytes (4, ⇔); production of hematopoietic cells (4, ⇔) | 39 ⇔ |
| Tissue develop | Survival of tissue (4, **⇓**) | 18 **⇓** |
| Cell compromise | Damage of cells (17, **⇑**); fragmentation of nucleus (8, **↓**); injury of cells (8, **⇑**); permeability transition (6, **⇑**); condensation of nucleus (5, ⇔); disruption of plasma membrane (3, **↑**); loss of actin stress fiber (3, **↑**); perturbation of mitotic spindle (2, **⇓**) | 38 **⇑** |
| Amino acid metab | Release of amino acids (9, ⇔); transport of aromatic amino acids (3, **⇑⇑**); depletion of S-adenosylmethionine (2, **⇑**); uptake of Ala (2, **⇑**) | 13 **↑** |
| Protein synthesis | Synthesis of protein (56, **↓**); translation (22, **⇓**); catabolism of glycoprotein (4, **↓**); | 60 **↓** |
| Drug metabolism | Metabolism of glutathione (8, **↓**); co-transport of glutathione (2, ⇔); release of glutathione (2, **↓**); secretion of epinephrine (2, **⇓**) | 16 **↓** |
| Gene expression | Expression of cAMP response element (7, **↑**); binding of Sis-inducible element (5, **⇑⇑**); activation of C/EBP binding site (4, ⇔) | 15 **↑** |
| Cell signaling | Flux of calcium (32, **↑**); influx of calcium (21, **↑**); quantity of nitric oxide (9, **⇑**); activation of JUN Kinase Kinase (3, **↓**) | 41 **↑** |
| Vit & Min metabol | Flux of calcium (32, **↑**); influx of calcium (21, **↑**) | 32 **↑** |
| Protein degradation | Catabolism of glycoprotein (3, **↓**) | 3 **↓** |
| Sk & musc D & F | Adhesion of smooth muscle cells (5,** ↓**) | 24 **↓** |
| Conn tissue D & F | Quantity of stromal cells (4, **↑**); contraction of fibroblasts (2, **⇓**) | 6 ⇔ |
| Carbohydrate met | Production of carbohydrates (26, ⇔); accumulation of carbohydrates (21, **↑**); | 50 **↑** |
| DNA rep rec & repa | Condensation of chromatin (11, **⇑**); cleavage of DNA (8, ⇔); breakage of DNA (6, **↓**) | 20 **↑** |

Functional annotation of Endocryne system development and function and protein trafficking did not make sense with respect to the mammary gland

^1^Included reproductive system development and function

**TABLE R8**. Tabulated results from Ingenuity Pathway Analysis® (IPA) at **60 vs. -30d**. Reported are the functions with an Exact Fisher test P-value ≤ 0.05 sorted by decrease in significance. The category denotes the main functional category assigned by IPA. The functional annotation was derived by the “effect on function” in IPA. In parenthesis are reported the number of DEG for each specific function and the arrows denote the overall effect on the function inferred by the gene annotation using IPA (⇑⇑ = highly activated; ⇑ = activated; 🡩 = tend to be activated; ⇓⇓ = highly inhibited; ⇓ = inhibited; 🡫 = tend to be inhibited). For detailed explanation see Additional file 2. Some annotations on functions are common between categories and were not repeated and the overall effect takes into consideration common functions among categories. Some categories do not make biological sense based on mammary gland features (see Additional file 2 for details explanation). For those two reasons the sum of the numbers in parenthesis very often does not correspond with the total DEG in the left column (DEG)

| **Category** | **Function Annotation** | **DEG** |
| --- | --- | --- |
| Protein Synthes | Stabilization of protein (12, **↓**) | 12 **↓** |
| End syst.D & F | Hormonal stress response of organism (27, ⇔); binding hormone (11, **⇓**); binding βestradiol (6, **↑**); size endocrine cell (6, ⇔); quantity acinar gland cell (3, **↑**) | 43 **↓** |
| Mol biochemistry | Production of glucose (15, **⇑**); quantity folate (5, **↑**), NADPH (4, **↑**); glycosylation AA (11, **⇑**); elimination of glucose (3, **↓**); metabolism adenine (3, **↓**) | 116 **⇑** |
| Drug metab | Transport of drug (6, **↑)** | 16 **↑** |
| Lipid metabolism | Quantity FA (35, **⇑**); lipid transport (30, **↑**); synthesis of sphingolipid (17, **⇑**), glycolipid (16, **⇑**), glycosylceramide (7, **⇑**), cerebroside (6, **⇑**), galactosylceramide (3, **⇑**); glycosphingolipid distribution (4, **↑**); phospholipid incorporation (6, **↓**); generation glycosphingolipid (6, **⇑**); cholesterol clearance (3, **⇑⇑**) | 88 **⇑** |
| Cellular assembly and organization | Vesicle transport (44, **↑**); actin filaments assembly and organization (22 and 21, **⇓**); neurites elongation (15, **⇓**); binding of cellular membrane (12, **↓**), cell surface (10, **↓**), chromosome components (10, **⇓**), chromatin (8, **⇓**); fusion of cellular membrane (10, ⇔), mitochondria (4, **⇓⇓**); biogenesis of lysosome (9, **⇓⇓**), peroxisome (7, **↓**), mitotic spindle (6, **⇓**); exocytosis of secretory granules (4, **⇓**); bundling of actin filaments (3, **⇓**) | 173 **⇓** |
| Cellular development | Differentiation (150, **↓**), neuron (56, **⇓**), leukocyte (30, **⇓**), epidermal cell (27, **⇓**), schwann cell (5, **⇓**); development fibroblast (88, **⇓**), neuron (77, **⇓**), muscle cell (65, **⇓**), epithelial cell (16, **↑**), schwann cell (9, **⇓**), vascular smooth muscle (9, **⇓⇓**); growth fibroblast (64, **⇓**); dedifferentiation (13, **↑**); maturation red blood cell (10, **⇑⇑**); delay maturation cell (3, **⇑⇑**); onset differentiation fibroblast (3, **⇑⇑**) | 333 **⇓** |
| Con tiss D & F | Mineralization connective tissue (20, **⇓⇓**); quantity stroma cell (5, **⇓⇓**); | 130 **⇓** |
| Tissue morphology | Remodeling of tissue (31, **↑**), connective tissue (30, **↑**); mass of muscle (11, **↑**); fusion of tissue (10, ⇔); quantity of fibroblasts (10, ⇔), parenchymal cells (6, ⇔), stromal cells (6, **⇓**), acinar gland cells (3, **↑**); thickness of mucosa (3, **↓**) | 75 **↑** |
| Cell cycle | G2 phase (43, **↓**), fibroblasts (10, **⇓**); mitosis (41, **⇓**), muscle cells (7, **⇓⇓**); delay in cell division (34, **↓**); senescence (29, **↑**); modification of chromosome components (23, **⇓**); S phase of fibroblasts (16, **↓**); arrest in mitosis (12, **⇓⇓**); entry into cell division process of fibroblasts (12, **⇓**); entry into interphase of fibroblasts (10, **↓**); initiation of interphase (9, **⇓⇓**); initiation of S phase (9, **⇓⇓**); cell division process of stem cells (8, **⇓⇓**), adipocytes (8, **↑**), brown adipocytes (5, **⇑**); delay in cell cycle progression (8, **⇓**); delay in mitosis (8, **⇓**); cell cycle progression of epithelial cells (7, **⇓**); delay in M phase (7, **↓**); biogenesis of mitotic spindle (6, **⇓**); mitogenesis of fibroblasts (6, **↓**);anaphase (5, **⇓⇓**); delay in cytokinesis (4, **⇑**); G0/G1 transition of fibroblasts (4, **⇓**) | 158 **⇓** |
| Cell death | Death of fibroblasts (97, **↓**), muscle cells (54, **⇓**), neurons (52, **⇓⇓**), adipocytes (4, **⇓**); activation-induced cell death (20, **↓**); survival of stem cells (13, **↓**), muscle cells (7, ⇔), neural stem cells (3, **⇑**); regeneration of muscle cells (4, **⇓⇓**); | 217 **⇓** |
| Tissue development | Patterning of connective tissue (31, **↑**); developmental process (14, **⇓**); adhesion of mast cells (8, **⇓⇓**), mesangial cells (6, ⇔); accumulation of neuroglia (4, **↓**), differentiation of muscle (4, **⇓**); clustering of T lymphocytes (3, **↑**); outgrowth of tissue (3, **↓**) | 79 **↓** |
| Cell morphology | Transformation of cells (137, **↓**), fibroblasts (78, **↓**), leukocytes (7, **↑**); elongation of plasma membrane projection (18, **↓**); extension of axons (12, **⇓**); size of endocrine cells (6, ⇔); frequency of nucleus (4, ⇔), micronuclei (3, **↑**); branching of fibroblasts (3, ⇔); morphology of microvascular endothelial cells (3, **↑**); size of macrophages (3, **↑**); transmembrane potential of neuroglia (3, ⇔) | 164 **↓** |
| Nerv sys D & F | Neurogenesis (96, **⇓**); differentiation of neuroglia (22, **⇓⇓**); extension of axons (12, **⇓**); neuritogenesis (10, **⇓⇓**); regeneration of axons (8, **⇓⇓**); migration of oligodendrocytes (6, **⇓**); accumulation of neuroglia (4, **⇓**), microglia (3, **⇓**) | 160 **⇓⇓** |
| Cellular growth and proliferation | Growth of fibroblasts (64, ⇔), vascular smooth muscle cells (7, **⇓**); formation of muscle cells (19, **↑**), myotube (16, **↑**); outgrowth of cells (9, **↑**); arrest in growth of leukocytes (7, **⇓**); hypertrophy of neuroglia (3, **⇑**) | 160 ⇔ |
| Cell-cell sig & int | Selection of T lymphocytes (11, **⇓**); binding of cell surface (10, **↑**); adhesion of mast cells (8, **⇓**); fusion of cells (8, **⇑**); | 59 ⇔ |
| Gene expression | Expression of mRNA (24, **↓**), p53 binding site (4, **⇓**); activation of p53 response element (7, **⇓⇓**), E2F binding site (5, **↓**), p73 binding site (3,); localization of mRNA (5, **↓**); repression of Gal4p binding site (4, **⇓⇓**); binding of CREB/ATF binding site (3, **⇓**) | 48 **⇓** |
| Sk muscle D & F | Mass of muscle (11, **↑**); survival of muscle cells (7, **↓**); differentiation of skeletal muscle (4, **⇓**) | 124 **↑** |
| Im Lym S D & F | Differentiation of leukocytes (30, **↓**); size of lymphoid organ (17, **↓**); size of macrophages (3, **↑**) | 55 **↓** |
| Organ morphol | Size of organ (45, **⇓**); morphogenesis of organ (33,** ↑** ) | 77 **↓** |
| Hem sys D & F | Maturation of red blood cells (10,** ⇑⇑**); clustering of T lymphocytes (3, **↑**); maturation of erythroblasts (3, **⇑**) | 51 **↑** |
| Post-transl mod | Activation of protein kinase (15, **⇑**); glycosylation of amino acids (11, **⇑**); methylation of protein (9, **⇓⇓**); ribosylation of protein (3, **⇓**) | 37 **↑** |
| Cell movement | Haptotaxis (10, **⇓⇓**); migration of oligodendrocytes (6,** ↓** ), naïve lymphocytes (3, **↑**); delay in cytokinesis (4, **⇑**) | 83 **⇓** |
| Molecular transport | Transport of protein (63, **↓**); quantity of glycolipid (20, **↑**), glycosylceramide (6, **⇑**), cerebroside (4, **↑**); transport of oligosaccharide (7, **↓**); exocytosis of protein (5, **⇑⇑**); nuclear export of RNA (5, **⇓⇓**) | 155 **↑** |
| Carbohydrate metabolism | Synthesis of glycogen (18, ⇔); activation of carbohydrate (5, **↓**); elimination of glucose (3, **⇓**) | 43 **↑** |
| Amino acid met | Glycosylation of amino acids (11, **⇑**); quantity of folic acid (5, **↑**) | 16 **⇑** |
| Cell function & maintenance | Assembly of actin filaments (22, **⇓**); organization of actin filaments (21, **⇓**); exocytosis of protein (5, **⇑⇑**), secretory granules (4, **⇓**) | 51 **⇓** |
| DNA rep rec repair | Checkpoint control (21, **⇓⇓**); cleavage of DNA (13, **⇓**), DNA fragment (6, **⇓⇓**); instability of genomes (7, **↑**); relaxation of DNA (5, **⇓⇓**) | 38 **⇓** |
| Protein trafficking | Transport of protein (63, **↓**); targeting of protein (21, **↓**); exocytosis of protein (5, **⇑⇑**) | 85 **↓** |
| RNA trafficking | Export of RNA (5, **⇓**); localization of mRNA (5, **↓**); nuclear export of RNA (5, **⇓**) | 8 **⇓** |
| Vit & Mineral Met | Quantity of folic acid (5, **↑**) | 5 **↑** |
| Card Syst D & F | Development of microvascular endothelial cells (3, ⇔) | 38 ⇔ |
| Organ develop | Morphogenesis of organ (33,** ↑** ) | 37 **↑** |
| Cell compromise | Instability if genome (7, **↑**) | 7 **↑** |
| Hair & Skin D & F | Differentiation of epidermal cells (27, **↓**); binding of keratanocytes (5, **⇑**); | 40 **↓** |
| Immune response | Immune response of organism (57, ⇔); antiviral response (21, ⇔) | 80 ⇔ |
| Nucleic acid met | Quantity of NADPH (4, **↑**); metabolism of adenine (3, **↓**) | 7 ⇔ |

Annotation of Reproductive system development and function did not make sense with respect to the mammary

**TABLE R9**. Tabulated results from Ingenuity Pathway Analysis® (IPA) at **120 vs. -30d**. Reported are the functions with an Exact Fisher test P-value ≤ 0.05 sorted by decrease in significance. The category denotes the main functional category assigned by IPA. The functional annotation is derived by the “effect on function” in IPA. In parenthesis are reported the number of DEG for each specific function and the arrows denote the overall effect on the function inferred by the gene annotation using IPA (⇑⇑ = highly activated; ⇑ = activated; 🡩 = tend to be activated; ⇓⇓ = highly inhibited; ⇓ = inhibited; 🡫 = tend to be inhibited). For detailed explanation see Additional file 2. Some annotations on functions are common between categories and were not repeated and the overall effect takes into consideration common functions among categories. Some categories do not make biological sense based on mammary gland features (see Additional file 2 for details explanation). For those two reasons the sum of the numbers in parenthesis very often does not correspond with the total DEG in the left column (DEG)

| **Category** | **Function Annotation** | **DEG** |
| --- | --- | --- |
| Gene expression | Activation binding site NFAT (11, **⇓), **p53 (10, **⇓⇓),** E2F (5,** ↓**), p73 (3, **⇓**); binding TPA RE (4, ⇔), CREB/ATF (3, **↓**); repression Gal4p (4, **⇓⇓**) | 34 **⇓** |
| Cell cycle | Entry into cell stage fibroblasts (13, **⇓**); initiation cell stage (10, **⇓⇓**); arrest cell stage fibroblasts (11, **⇓**); cell division process adipocytes (8, **↑**), leukocyte (24, **↑**), stem cell (7, **↓**), chromosome (5, **↑**); entry S phase fibroblasts (12, **↓**); S phase fibroblasts (16, **⇓**); initiation S phase (9, **⇓⇓**); arrest interphase fibroblasts (11, **⇓**); delay G2/M phase transition (3, **↑**); G2/M phase transition (18, **⇓⇓**); mitosis (72, **⇓⇓**), mitosis muscle (7, **⇓⇓**), fibroblasts (4, **↓**); senescence (29, **⇓**) fibroblasts (11, **⇓**); aneuploidy (10, **⇑**); G0/G1 phase transition (21, **⇓**); G2/M phase fibroblasts (3, **⇓**); G2 phase (39, **↓**); modification chromosome components (23, **⇓**); fission (5, **↓**) | 184 **⇓** |
| Cell assemb & organization | Biogenesis peroxisomes (7, **↓**), lysosome (8, **⇓**); extension axons (13, **↓**); quantity filopodia (7, **↑**); quantity centrosome (10, **↑**); fusion mitochondria (4, **⇓**); establishment chromatin structures (7, **⇓**) | 57 **⇓** |
| Cell death | Apoptosis of fibroblasts (71, **↓**), adipocytes (4, **↓**); cell death of leukocytes (64, **↓**), neurons (51, **⇓**), muscle cells (22, **↓**), exocrine cells (7, **⇓**); cell viability (53, ⇔); self-renewal of cells (16, ⇔), stem cells (9, ⇔); survival of stem cells (12, **⇓**), muscle cells (6, **⇑**), neural stem cells (3, **↑**); regeneration of axons (8,** ⇓** ); lysis of fibroblasts (4, **⇑**); regeneration of muscle cells (4, **⇓⇓**) | 241 **↓** |
| Conn T D & F | Growth of fibroblasts (66, ⇔); mineralization of connective tissue (18, **↓**); quantity of adipose tissue (16, **⇓**), stromal cells (5, ⇔); quantity of fibroblasts (9, **⇓**); colony formation of fibroblasts (8, ⇔); stimulation of fibroblasts (7, **⇑**); mitogenesis of brown adipocytes (3, **⇑**) | 138 **↓** |
| Protein synthesis | Stabilization of proteins (11, **⇓**) | 11 **⇓** |
| End sys D & F | Binding of hormone (10, **⇓⇓**), β-estradiol (5, **↑**); differentiation of neuroendocrine cells (5, **⇓**); size of endocrine cells (5, **↓**); quantity of acinar gland cells (3, **↑**) | 21 **↓** |
| Small mol bioch | Modification of amino acid (142, **↓**); moiety attachment to amino acid (117, **↓**); production of inositol phosphate (18, **⇓**); production of glucose (13, **↑**); biosynthesis of proteoglycan (7, **⇑**), phosphatidylcholine (7, **↑**), glycoceramide (5, **⇑**); generation of glycosphingolipid (6, **⇑**); distribution of glycosphingolipid (4, **⇑**), ganglioside (3, **⇑**); clearance of cholesterol (3, **⇑**) | 200 **↓** |
| Cellular development | Developmental process of fibroblasts (86, **↓**), neurons (73, **⇓**), leukocytes (33, ⇔), myoblasts (23, **⇓**), vascular smooth muscle (9, **⇓⇓**); differentiation of epithelial cells (51, **⇓**), epidermal cells (28, ⇔), leukocytes (27, **⇓**), myoblasts (22, **⇓**), oligodendrocytes (10, **⇓**), melanocytes (9, **↑**), neuroendocrine cells (5, **⇓⇓**); senescence of fibroblasts (11, **↓**); maturation of red blood cells (9, **⇑⇑**); self-renewal of stem cells (9, **↓**); arrest in developmental process in leukocytes (7, **↓**); regeneration of muscle (4, **⇓⇓**); delay in maturation of cells (3, **⇑**); onset of differentiation of connective cells (3, **⇑**) | 282 **⇓** |
| Cell growth & proliferation | Proliferation (485, ⇔); growth (232, ⇔), fibroblasts (66, ⇔), leukocytes (33, **↑**), vascular smooth muscle (7, ); arrest growth (46, **⇓**), leukocytes (7, **↓**); colony formation of connective cells (12, **↑**); proliferation of mesenchymal cells (10, **⇑**); outgrowth (9, ⇔); stimulation of fibroblasts (7, **⇑**) | 565 ⇔ |
| Tissue morphology | Remodeling of connective tissue (26, ⇔); quantity of adipose tissue (16, **⇓**), fibroblasts (9, **⇓**), intraepithelial T lymphocytes (5, **⇓⇓**), stromal cells (5, ⇔), acinar gland cells (3, **↑**); area of blood vessel (3, **⇑**); thickness of mucosa (3, **↓**) | 71 **↓** |
| Cell-to-cell sign & interaction | Neurotransmission of synapse (11, **↑**); adhesion of mast cells (8, **⇓**), microvascular endothelial cells (6, ⇔); fusion of cells (8,** ↑**), muscle cells (5, **↑**); binding of keratanocytes (5, **⇑⇑**) | 47 **↑** |
| Hem Sys D & F | Growth of leukocytes (33, **↑**); differentiation of leukocytes (27, **⇓**); maturation of red blood cells (9, **⇑⇑**), erythroblasts (3, **⇑**); adhesion of mast cells (8, **⇓**); arrest growth of leukocytes (7, **↓**); quantity of intraepithelial T lymphocytes (5, **⇓**); size of macrophages (3, **↑**) | 63 **↑** |
| Im & Lym S D&F | Size of lymphoid organ (15, **↓**), bone marrow cells (5, **⇑**); macrophages (3, **↑**) | 67 **↓** |
| Tissue develop | Development of exocrine gland (28, **↑**), mammary gland (24, **↑**); survival of tissue (5, **⇓⇓**); accumulation of neuroglia (4, **↓**) | 81 **↑** |
| Sk & musc D & F | Skeletal and muscular process (20, **⇓**); formation of muscle cells (17, **↑**); growth of vascular smooth muscle cells (7, **⇓⇓**); formation of myosin stress fiber (5, **↓**); fusion of muscle cells (5, ⇔) | 137 **↓** |
| Cell morphology | Length of plasma membrane projection (15, ⇔), neurites (13, ⇔); transformation of leukocytes (8, **⇑**); frequency of nucleus (4, ⇔), micronuclei (3, **↑**); vacuolation of cells (3, **↓**) | 55 ⇔ |
| Lipid metabolism | Synthesis of phosphatidylcholine (7, **↑**), glycoceramide (5, **⇑**); generation of glycosphingolipid (6, **⇑**); binding of βestradiol (5, **↑**); distribution of glycosphingolipid (4, **⇑**), ganglioside (3, **⇑**); clearance of cholesterol (3, **⇑**) | 25 **⇑** |
| Mol transport | Transport of organic cation (7, **⇑⇑**), drug (5, **⇑**), sugar acid (3, **↓**); uptake of ascorbic acid (3, **⇓**) | 23 **⇑** |
| Nerv sys D & F | Neurogenesis (92, **⇓**); neurological process (57, **↑**); motor function (19, **↑**); regeneration of axons (8, **⇓**); migration of oligodendrocytes (6, **↓**); accumulation of neuroglia (4, **↓**), microglia (3, **↓**) | 171 **↓** |
| Drug metabolism | Binding of βestradiol (5, **↑**); transport of drug (5, **⇑**) | 10 **⇑** |
| DNA rep rec rep | Checkpoint control (22, **⇓⇓**); modification of chromatin (22, **⇓**); cleavage of DNA (13, **⇓⇓**), DNA fragments (5, **⇓⇓**); quantity of centrosome (10, **⇓**); aberration of chromosomes (5, **⇓⇓**); relaxation of DNA (5, **⇓⇓**); initiation of synthesis of DNA (4, **⇓⇓**) | 67 **⇓⇓** |
| Hair & skin D&F | Differentiation of epidermal cells (28, ⇔), melanocytes (9, **↑**); pigmentation (19, **⇓**); binding of keratanocytes (5, **⇑⇑**); dermatological process of mice (5, **⇓**) | 45 **↓** |
| Card sys D & F | Adhesion of microvascular endothelial cells (6, **↓**); area of blood vessel (4, **⇑**) | 38 ⇔ |
| Organ morphol | Morphogenesis of organ (31, ⇔); size of lymphoid organ (15,** ↓** ) | 50 **↓** |
| Amino acid met | Modification of amino acid (142, **↓**); moiety attachment to amino acid (117, **↓**) | 142 **↓** |
| Post-trans modif | Methylation of proteins (8, **⇓⇓**); O-glycosylation (7, **⇓⇓**); regulation of kinase (5, **⇑⇑**); ribosylation of protein (3, **⇓**) | 162 **⇓** |
| Carbohyd met | Production of carbohydrates (⇔); regulation of carbohydrates (5, **↓**) | 61 **↑** |
| Cell Func & Main | Contact growth inhibition (3, **⇓**); cytostasis (3, **⇓**) | 3 **⇓** |
| Cell movement | Migration of oligodendrocytes (6, **↓**), naïve lymphocytes (3, **↑**) | 9 **↓** |
| Vit & Mineral Met | Uptake of ascorbic acid (3, **⇓**) | 3 **⇓** |
| RNA post-tr mod | Modification of RNA fragments (4, **⇓⇓**) | 4 **⇓⇓** |
| Organ develop | Morphogenesis of organ (31, ⇔); development of mammary gland (24, **↑**) | 52 **↑** |
| Repr sys D & F | Maternal behavior (4, ⇔) | 33 **↑** |
| Cell compromise | Degradation of cell (5, **↓**), mitochondria (5, **⇑⇑**) | 10 **⇑** |
| Imm response | Cytotoxic reaction (7, **↑**); migration of naïve lymphocytes (3, **↑**) | 7 **↑** |

**TABLE R10**. Tabulated results from Ingenuity Pathway Analysis® (IPA) at **240 vs. -30d**. Reported are the functions with an Exact Fisher test P-value ≤ 0.05 sorted by decrease in significance. The category denotes the main functional category assigned by IPA. The functional annotation is derived by the “effect on function” in IPA. In parenthesis are reported the number of DEG for each specific function and the arrows denote the overall effect on the function inferred by the gene annotation using IPA (⇑⇑ = highly activated; ⇑ = activated; 🡩 = tend to be activated; ⇓⇓ = highly inhibited; ⇓ = inhibited; 🡫 = tend to be inhibited). For detailed explanation see Additional file 2. Some annotations on functions are common between categories and were not repeated and the overall effect takes into consideration common functions among categories. Some categories do not make biological sense based on mammary gland features (see Additional file 2 for details explanation). For those two reasons the sum of the numbers in parenthesis very often does not correspond with the total DEG in the left column (DEG)

| **Category** | **Function Annotation** | **DEG** |
| --- | --- | --- |
| Cellular Growth and Proliferation | Proliferation (243, **↑**), muscle (27,** ⇑**), pericytes (6, **⇑**), blood cells (63, **↑**); growth (198, **↓**), smooth muscle (8, **⇑**); colony formation (63, **↓**); formation of cells (39, **↑**), connective tissue cells (13, **↑**) | 332 **⇑** |
| Cellular Movement | Movement (134, **↑**), mononuclear leukocytes (26, **⇑**), epithelial (14, **⇓**), leukocytes (42,), keratanocytes (8, **⇓⇓**), neutrophils (25, **⇑**), bone marrow cells (32, **⇑**), myeloid cells (31, **⇑**), macrophages (17, **⇑⇑**), monocytes (14, **⇑⇑**), lymphocytes (20, **⇑**), antigen presenting cells (21, **⇑**), endothelial cells (27, ⇔), stem cells (5, **⇑**); homing of cells (48, **↑**); invasion of cells (60, **⇑**); mobilization of phagocytes (6, **⇑**), chemotaxis (24, **⇑**); segregation of lymphocytes (3, **↓**); chemokinesis of leukocytes (4, **⇑**); infiltration of plasma cells (3, **↑**) | 186 **⇑** |
| Cell-To-Cell Signaling and Interaction | Adhesion (100, **↑**), fibroblasts (9, **⇔**), breast cell lines (3, **↑**), lymphatic system (25,** ⇑⇑**); attachment of cells (20, **⇑**); activation of cells (73, **⇑**); quantity of focal adhesions (9, ⇔); recruitment of cells (25, **⇑⇑**), phagocytes (16, **⇑⇑**), T lymphocytes (7, **⇑**); function of synapse (3, **⇑**); attraction of macrophages (4, **↑**); fusion of cell lines (5, **↑**); phagocytosis of cells (19, **⇑**) | 163 **⇑** |
| Tissue Development | Developmental process of muscle (40, **↑**), cardiovascular tissue (13, **↑**), tissue (93, **⇑**); development of exocrine gland (15, **⇑**); remodeling of connective tissue (15, **⇑**), formation of tissue (37, ⇔); accumulation of lymphocytes (10, **↓**); assembly of extracellular matrix (9, **⇑**) | 182 **⇑** |
| Card Syst Devel & Function | Development of blood vessel (52, **↑**); angiogenesis (41, **↑**); patterning of blood vessel (13, **⇑**); adhesion of endothelial cells (15, **↑**); cardiovascular process of blood vessel (31, **↑**); formation of endothelial tube (11,** ⇔** ) | 70 **↑** |
| Tissue Morphology | Quantity of cells (126, **↑**), blood cells (66, **↓**); thickness of tissue (14, **↓**); size of lesion (14, **⇑**), vessel (5, **↑**); morphology of tissue (17, **⇑**), connective tissue (6, **⇑**); mass of tissue (19, **⇑**) | 159 **⇑** |
| Cell Morphology | Morphogenesis cells (66, **⇑**); morphology cells (61, **⇑**), filaments (4, **⇑**); shape change (63, **⇑**), connective tissue cells (9, **⇑⇑**); cell spreading of connective tissue cell (9, **⇑⇑**); cell rounding (13, **⇑**); transmembrane potential mitochondrial membrane (5, **↑**); polarization of phagocytes (5, **⇑⇑**) | 120 **⇑⇑** |
| Connect D. & F. | Adipogenesis of cells (12, **↑**); differentiation of fibroblasts (17, **↓**); colony formation of fibroblasts (15, **⇓**); lipolysis of adipocytes (4, **↑**) | 74 **↓** |
| Cell Death | Cell death (255, **↑**), neurons (46, ⇔), bone marrow cells (16, **↓**); cell viability (37, **↑**); lysis of fibroblasts (3, **↑**) | 262 **↑** |
| Cellular Development | Differentiation of cells (151, **↑**), epithelial cells (28, **⇑**), fibroblasts (17, **↓**), granulocytes (11, **↑**), muscle cell lines (18, ⇔), neurons (26, **↑**); development of cells (125, **⇑**), granulocytes (13, ⇔), epithelial cell lines (15, **⇑**), neurons (36, **⇑**), blood cells (78, ⇔), bone marrow cells (29, **⇑**), endothelial cell lines (15, ⇔), fibroblasts (38, **↑**); metaplasia of epithelial cells (3, **↑**) | 233 **⇑** |
| Cell compromise | Injury of cells (9, **⇑⇑**); damage of cells (13, **⇑**); disassembly of actin filaments (9, **↑**); oxidative stress response of cells (8, **↑**) | 34 **⇑** |
| Cell Cycle | Cell division (129, **↑**); cell stage (56, **↓**); delay in cell stage (11, **⇓**); arrest of G2/M phase transition (8, **⇓**); delay in mitosis (6, **⇓⇓**) | 132 **↑** |
| Cellular Assembly and Organization | Formation of peroxisomes (2, **⇓**); formation of filaments (38, **⇑**); assembly of filaments (20, **↑**); fusion of cellular membrane (7, **⇑**); quantity of chromosomes (4, **⇓**), function of synapse (3, **⇑**); outgrowth of axons (9, **↑**); organization of cells (7, **⇑**) | 78 **⇑** |
| Cell F & Maint | Engulfment of cells (24, **↑**); endocytosis (22, **↓**); phagocytosis of cells (19, **↑**) | 57 **↑** |
| Ske mus D & F | Proliferation of muscle cells (27, **⇑**); differentiation of muscle cells (18, ⇔); growth of smooth muscle cells (8, **⇑⇑**) | 93 **⇑** |
| Immune response | Immune response (81, **↑**); movement granulocyte (30, **⇑**); immune response organism (29, ⇔); inflammation organ (22, **↓**); phagocytosis (19, **↑**); accumulation lymphocyte (10, **↓**); recruitment T lymphocytes (7, **⇑**); aggregation antigen presenting cells (5, **↑**); chemokines leukocyte (4, **⇑**) | 115 **↑** |
| Cell signaling | Quantity of calcium (47, **⇑**); RAC protein signaling transduction (6, ⇔) | 53 **↑** |
| Hem sys D & F | Hematopoiesis (63, ⇔); quantity of leukocytes (56, ⇔); accumulation of lymphocytes (10, **↓**), T lymphocytes (8, **↓**); recruitment of T lymphocytes (7, **⇑**); chemokinesis of leukocytes (4, **⇑**); infiltration of plasma cells (3, **⇑**); release of neutrophils (3, **↑**); segregation of B lymphocytes (3, **↓**); stimulation of colony-forming units granulocytes (2, **⇑**) | 117 **↑** |
| DNA rep rec rep | Synthesis of DNA (53, **↑**); cleavage of DNA fragment (4, **↓**); quantity of chromosomes (4, **⇓⇓**); recombination of chromosomes (3, **↑**) | 62 **↑** |
| Repr sys D & F | Fertility (18, **↓**) | 37 **↓** |
| Gene expression | Binding of cAMP response element (7, **⇓⇓**); expression of DNA endogenous promoter (7, **↑**); activation of TATA box (3, **↑**) | 17 **↓** |
| Carbohyd met | Metabolic process of carbohydrates (46, **↑**), monosaccharide (13, **↓**); production of glucose (9, **⇑⇑**) | 49 **↑** |
| Small molecular biochememistry | Quantity of lipid (48, **↑**), acylglycerol (23, ⇔), triacylglycerol (21, ⇔), fatty acid (12, **⇑**); modification of fatty acid (21, **⇑**); concentration of fatty acid (4, **⇓**); biosynthesis of prostaglandin E2 (3, **⇓**); synthesis of dopamine (3, **↓**), hydrocortisone (3, **↓**); degradation of L-dopa (2, **⇓**); metabolism of choline (2, **⇓**); quantity of palmitoleic acid (2, **⇑**) | 64 **↑** |
| Organismal dev | Development of vessel (52, **⇑**); size of vessel (5, **⇑⇑**) | 52 **⇑** |
| Drug metabolism | Biosynthesis of prostaglandin E2 (3, **↓**), hydrocortisone (3, **↓**); synthesis of dopamine (3, **↓**); degradation of L-dopa (2, **⇓**) | 9 **↓** |
| End Sys D & F | Biosynthesis of hydrocortisone (3, **↓**); quantity of corticotroph cells (2, **⇓**) | 5 **↓** |
| Imm & Ly S D & F | Quantity of leukocytes (56, ⇔); differentiation of myeloid cells (17, **⇑**), leukocytes (14, **↑**); accumulation of lymphocytes (10, **↓**), T lymphocytes (8, **⇓**); degranulation of mast cells (8, ⇔); segregation of B lymphocytes (3, **↓**); stimulation of colony-forming units granulocytes (2, **⇑**) | 81 **↑** |
| Lipid metabolism | Quantity of lipid (48, **↑**), acylglycerol (23, ⇔), triacylglycerol (21, ⇔), fatty acid (12, **⇑**); modification of fatty acid (21, **⇑**); lipolysis of adipocytes (4,); concentration of fatty acid (4, **⇓**); biosynthesis of prostaglandin E2 (3, **↓**); hydrocortisone (3, **↓**); quantity of palmitoleic acid (2, **⇑**) | 59 **↑** |
| Nerv sys D & F | Outgrowth of axons (9, **↑**); nociception (6, **↓**); differentiation of interneurons (3, **↓**); function of synapse (3, **⇑**); patterning of axons (3, **↓**); size of presynaptic terminals (2, **↑**) | 49 **↑** |
| Organ develop | Organogenesis (65, **⇑**) | 67 **⇑** |
| Mol transport | Quantity of calcium (47, **⇑**); secretion of reactive oxygen species (3, **⇑**); concentration of bicarbonate (2, **⇑**) | 83 **⇑** |
| Free radic scav | Secretion of reactive oxygen species (3, **⇑**) | 3 **⇑** |
| Hair & skin D & F | Differentiation of epithelial cell lines (5, **⇑⇑**); dermatological process of wound (4, **↑**); fragility of skin (2, **⇓**) | 11 **↑** |
| Amino acid metab | Degradation of L-dopa (2, **⇓**) | 2 **⇓** |
| Vit & Miner Metab | Quantity of calcium (47, **⇑**) | 47 **⇑** |

Organ morphology was not considered because it was not pertinent to mammary gland

**TABLE R11**. Tabulated results from Ingenuity Pathway Analysis® (IPA) at **300 vs. -30d**. Reported are the functions with an Exact Fisher test P-value ≤ 0.05 sorted by decrease in significance. The category denotes the main functional category assigned by IPA. The functional annotation is derived by the “effect on function” in IPA. In parenthesis are reported the number of DEG for each specific function and the arrows denote the overall effect on the function inferred by the gene annotation using IPA (⇑⇑ = highly activated; ⇑ = activated; 🡩 = tend to be activated; ⇓⇓ = highly inhibited; ⇓ = inhibited; 🡫 = tend to be inhibited). For detailed explanation see Additional file 2. Some annotations on functions are common between categories and were not repeated and the overall effect takes into consideration common functions among categories. Some categories do not make biological sense based on mammary gland features (see Additional file 2 for details explanation). For those two reasons the sum of the numbers in parenthesis very often does not correspond with the total DEG in the left column (DEG)

| **Category** | **Function Annotation** | **DEG** |
| --- | --- | --- |
| Cell Death | Lysis of cells (12, **⇑⇑**), fibroblast cell lines (4, **⇑**); cytolysis of fibroblast cell lines (3, **↑**), endothelial cells (3, **↓**) | 22 **⇑** |
| Cellular Assembly and Organization | Transport of vesicles (20, **⇑**); biogenesis of organelle (17, **↑**); fusion of cellular membrane (8, **↑**); modification of cytoskeleton (2, **⇑**); removal of filaments (2, **⇑**); morphology of fibrils (2, **⇑**); quantity of ribosome (2, **↓**); shortening of filaments (2, **⇑**) | 50 **⇑** |
| Cell morphology | Transformation of epithelial cells (6, ⇔); length of filaments (5, **↓**); shape change and spreading of epithelial cells (5, **⇑**); vacuolation of cells (4, **↑**); morphology of stem cells (3, **↑**) | 33 **↑** |
| Cellular movement | Cell movement (99, **↑**); migration of endothelial cells (25, **↑**), neurons (19, **↑**); epithelial cells (7, **↑**), granulocytes (3, **⇓**), memory T cells (3, **⇑**), naïve lymphocytes (3, **↑**); localization of leukocytes (5, **↑**); rolling of granulocytes (4, ⇔); segregation of lymphocytes (3, **↓**) | 127 **↑** |
| Nucleic acid met | Metabolism of adenine (3, **↓**); salvage of adenine (2, ⇔); synthesis of CTP (2, **⇓**) | 5 **↓** |
| Small molecular biochemistry**^1^** | Phosphorylation of amino acid (45, **↑**); modification of fatty acid (20, **↑**); oxidation of fatty acid (15, **↑**); quantity of cholesterol ester (6, **↑**), fat (4, **⇑**); storage of lipid (6, **⇓**); production of acylglycerol (5, **↓**); release of norepinephrine (5, **⇓**); synthesis of phosphatidylcholine (5, **↓**); binding of progesterone (4, **⇓⇓**); deposition of lipid (4, **⇑⇑**); metabolic process of polyols (4, **⇑⇑**); production of diacylglycerol (4, **⇓**); transport of long chain fatty acid (4, **⇑**); accumulation of nitrite (3, **⇓**); uptake of taurocholic (3, **↓**); metabolism of adenine (3, **↓**); synthesis CTP (2, **⇓**) | 102 **↑** |
| Cell funct & maint | Presence of leukocytes (6, **↑**), lymphocytes (4, **↑**); localization of leukocytes (5, **↑**); pinocytosis (4, **⇓**), of protein (2, **⇓**); phagocytosis of fibroblasts (3, **⇓**) | 16 ⇔ |
| Hem sys D & F | Cell movement of leukocytes (48, **↑**), myeloid cells (29, **↓**); differentiation of Th2 lymphocytes (8, **↑**); activation of leukocytes (5, **↑**), mast cells (5, **↑**), peripheral blood leukocytes (4, **⇑**), helper T lymphocytes (2, **⇑**); development of regulatory T lymphocytes (2, **⇑**); stimulation of Th1 lymphocytes (2, **⇑**) | 64 **↑** |
| Immune response | Degranulation of mast cells (7, **↓**); selection of T lymphocytes (6, **⇓**); antiviral response (3, **⇓**); biosynthesis of cytokines (3, **↓**); secretion of cytokines (3,** ⇑** ); Th2 immune response of organism (2, **⇓**) | 68 **↓** |
| Im & lym S D & F | Differentiation of Th2 lymphocytes (8, **↑**); segregation of lymphocytes (3, **↓**); quantity of colony-forming units granulocytes (2, **⇓**) | 37 **↑** |
| Conn tiss D & F | Growth of fibroblasts (28, ⇔); mass of connective tissue (16, **↓**), adipose tissue (11, **↓**); development of connective tissue (12, ⇔); adipogenesis (10, **⇓**), fibroblasts (7, **↓**); stimulation of fibroblasts (4, **⇑⇑**); cell movement of dermal fibroblasts (3, **↓**); deposition of subcutaneous fat (2, **⇑**); shape change in dermal fibroblasts (2, **⇑**) | 76 **↓** |
| Tissue develop | Patterning of tissue (18, **⇑**), connective tissue (16, **⇑**); remodeling of tissue (17, **⇑**), connective tissue (16, **⇑**); adhesion of neurons (3, **↑**); aggregation epithelial cells (3, **↑**); accumulation microglia (2, **⇓**) | 30 **⇑** |
| Tissue morphology | Mass of tissue (18, **↓**); thickness of tissue (11, **↓**); deterioration of tissue (4, ⇔); quantity of parenchymal cells (4, ⇔), colony forming-units granulocytes (2, **⇓**); size of blood vessel (4, ⇔) | 53 ⇔ |
| Cell growth & prol | Proliferation (217, ⇔); growth (188, ⇔), fibroblasts (28, ⇔) | 296 ⇔ |
| Cell development | Development (114, **↑**), oligodendrocytes (4, ⇔), myofiber (2, **⇑**), regulatory T lymphocytes (2, **⇑**); developmental process of fibroblasts (36, ⇔), Th2 lymphocytes (9, ⇔); differentiation of interneurons (3, **↓**) | 145 ⇔ |
| Lipid metabolism | Modification of fatty acid (20, **⇑**); oxidation of fatty acid (15, **↑**); quantity of free fatty acid (11, **⇑**), cholesterol ester (6, **↑**), palmitoleic acid (2, **⇑**); storage of lipid (6, **↓**); production of acylglycerol (5, ⇔), diacylglycerol (4, **↑**); synthesis of phosphatidylcholine (5, **↓**); binding of progesterone (4, **⇓⇓**); deposition of lipid (4, **⇑⇑**); quantity of fat (4, **⇑**), phosphatidylserine (4, ⇔); transport of long chain fatty acid (4, **⇑**); accumulation of monounsaturated fatty acid (3, **⇑**); transport of palmitic acid (3, **⇑**); uptake of taurocholic (3, **↓**); lipolysis of triacylglycerol (2, **⇑**) | 51 **⇑** |
| Gene expression | Expression of mRNA (12, **↓**); transcription of protein binding site (8, **↑**), adipose response element (2, **⇓**); activation of NFAT response element (6,** ↑** ); activation of C/EBP binding site (3, **↑**), Myb binding site (3, **↓**), farnesoid X receptor response element (2, **⇓**); binding of Hnf1 binding site (2, **⇓**) | 31 **↓** |
| Drug metabolism | Release of norepinephrine (5, **⇓⇓**); binding of progesterone (4, **⇓⇓**) | 9 **⇓⇓** |
| End Sys D & F | Binding of progesterone (4, **⇓⇓**) | 4 **⇓⇓** |
| Cell-cell sign & int | Binding of cells (58, **↓**); attachment (17, ⇔); adhesion of epithelial cells (8, **⇑⇑**), neurons (3, **↑**); selection of T lymphocytes (7, **⇓**); activation of leukocytes (5, **↑**), mast cells (5, **↑**); allostimulatory capacity of cells (2, **⇑**) | 84 ⇔ |
| Card sys D & F | Size of blood vessel (4, **↓**); remodeling of vasculature (3, **↓**) | 22 **↓** |
| Organ develop | Development of epidermis (10, **⇑**) | 22 **⇑** |
| Carbohydrate met | Metabolic process of polyols (4, **⇑⇑**) | 8 **⇑** |
| Cell cycle | Cell cycle progression of epithelial cells (4, ⇔); endoreduplication (4, **↓**); arrest in cell cycle progress of blood platelets (2, **⇑**) | 20 ⇔ |
| Nerv Sys D & F | Migration of neurons (19, **↑**); development of oligodendrocytes (4, ⇔); adhesion of neurons (3, **↑**); differentiation of interneurons (3, **↓**); accumulation of microglia (2, **⇓**); size of presynaptic terminals (2, **⇓**) | 29 **↑** |
| Cell compromise | Damage of cells (13, ⇔); degranulation of mast cells (7, **↓**); acidification of lysosome membrane (2, **⇓**) | 25 **↓** |
| Hair & skin D & F | Development of epidermis (10, **⇑**); re-epithelialization (5, **⇑**), of wound (3, **⇑**); fragility of skin (2, **⇓**); laxity of skin (2, **⇓**) | 27 **⇑** |
| Mol transport | Secretion of protein (11, **↓**); accumulation of anion (4, **⇓**), calcium (4, ⇔), nitrite (3, **↓**); concentration of bicarbonate (2, **⇑**); pinocytosis of protein (2, **⇓**) | 48 **↓** |
| Protein trafficking | Secretion of protein (11, **↓**), cytokines (3, **↑**); pinocytosis of protein (2, **⇓**) | 13 **↓** |
| Protein synthesis | Synthesis of protein (42, **⇓**); translation (17, **⇓**); elongation of protein (3, **⇓**) | 42 **⇓** |
| Organismal func | Thermoregulation (8, **↑**); recovery (4, **⇓**) | 12 **↓** |
| Amino acid met | Phosphorylation of amino acids (45, **↑**); clearance of amino acids (2, **⇑**) | 47 **↑** |
| Post-transl mod | Phosphorylation of amino acids (45, **↑**); association of proteins (3, **↓**); binding of protein fragment (2, ⇔); ribosylation of protein (2, **⇓**) | 50 **↑** |
| Cell signaling | Inactivation of MAP kinase (4, **⇓**); presence of calcium (3, **⇑**) | 11 **↓** |
| Vit Mineral Metab | Accumulation of calcium (4, ⇔); presence of calcium (3, **↑**) | 7 **↑** |
| Organismal dev | Production of bodily fluid (6, ⇔) | 8 ⇔ |

Organ morphology, Reproductive system development and function, and Organismal suvival were not considered because they were not pertinent to mammary gland

**TABLE R12**. Tabulated results from Ingenuity Pathway Analysis® (IPA) at **1 vs. -15d**. Reported are the functions with an Exact Fisher test P-value ≤ 0.05 sorted by decrease in significance. The category denotes the main functional category assigned by IPA. The functional annotation is derived by the “effect on function” in IPA. In parenthesis are reported the number of DEG for each specific function and the arrows denote the overall effect on the function inferred by the gene annotation using IPA (⇑⇑ = highly activated; ⇑ = activated; 🡩 = tend to be activated; ⇓⇓ = highly inhibited; ⇓ = inhibited; 🡫 = tend to be inhibited). For detailed explanation see Additional file 2. Some annotations on functions are common between categories and were not repeated and the overall effect takes into consideration common functions among categories. Some categories do not make biological sense based on mammary gland features (see Additional file 2 for details explanation). For those two reasons the sum of the numbers in parenthesis very often does not correspond with the total DEG in the left column (DEG)

| **Category** | **Function Annotation** | **DEG** |
| --- | --- | --- |
| Cell Death | Apoptosis (302, **↓**), fibroblast cell lines (42, **↑**); inhibition of apoptosis (39, **⇓**); survival of stem cells (8, **↑**), fibroblast cell lines (12, **⇓**); self-renewal of neural stem cells (3, **↑**) | 370 **↓** |
| Cellular Development | Developmental process of fibroblast cell lines (60, **⇓**), stem cells (30, **↓**), natural killer cells (8, **↑**), muscle cells (38, **⇓**), endothelial cells (21, **↓**); lifespan of epithelial cells (3, **⇓⇓**); morphogenesis of epithelial cells (4, **↓**); differentiation of stem cells (18, **↓**); differentiation of mesenchymal stem cells (3, **↑**); maturation of bone marrow cells (8, **↑**) | 149 **⇓** |
| Cellular Growth and Proliferation | Growth of cells (262, **↓**), fibroblasts (45, **⇓**), epithelial cells (16, **⇓**), blood cells (31, **↑**), stem cells (8, ⇔); proliferation (279, **↓**), fibroblasts (38, **⇓**); colony formation of leukocytes (8, **↑**), fibroblasts (18, **↓**); production of cells (11, **↑**) | 416 **↓** |
| Cell Cycle | Cell stage (129, **↓**), fibroblasts (20, **⇓**); entry into cell stage of fibroblasts (9, **↓**); arrest in cell stage of fibroblasts (9, **↓**); delay in cell stage (20, **↑**); exit from cell stage (9, **↓**); cell stage of stem cells (4, ⇔); interphase (73, **↓**), fibroblasts (18, **⇓**); entry into interphase (23, **↓**), fibroblasts (8, **↓**); arrest in interphase of bone marrow cells (3, **⇑⇑**); G2 phase (29, **↓**), fibroblasts (9, **↓**);arrest in G2 phase (20, **⇓**), fibroblasts (6, **↓**); S phase (38, **⇓**), fibroblasts (13, **⇓**); entry into S phase (23, **↓**), fibroblasts (8, **↓**); delay in cell division process (25, **↓**); cell division process (171, **↓**), fibroblasts (25, **↓**); stem cells (5, **↑**); arrest in cell division process (63, **↓**), fibroblasts (13, **⇓**); delay in cell division process (20, **↓**); cell cycle progression (124, **⇓**); arrest in cell cycle progression (50, **↓**), fibroblasts (7, **↓**); termination of cell cycle progression of fibroblasts (3, **⇓⇓**); G2/M phase (13, **↓**), fibroblasts (3, **⇓⇓**); arrest in G2/M phase (8, **↑**), bone marrow cells (2, **⇑**), fibroblasts (2, **↓**), lymphocytes (2, **⇑**); spindle checkpoint of fibroblasts (3, **⇓⇓**); biogenesis of mitotic spindle (5, **⇓**); G2/M phase transition (13, **⇓**), fibroblasts (4, ⇔); arrest in G2/M phase transition (10, **↑**); G1 phase of stem cells (2, **↑**); arrest in G1 phase of fibroblasts (4, **↓**); delay in cytokinesis (3, **↑**); formation of mitotic spindle (9, **⇓**); mitosis (44, **⇓⇓**); polyploidization (5, **⇓⇓**); length of telomeres (6, **⇓⇓**) | 195 **⇓** |
| Gene Expression | Transcription (250, **↓**); repression of Gal4p binding site (4, **⇓⇓**); binding of RNA (6, **⇑**); activation of TCF binding site (3, **↓**); activation of LEF1 binding site (5, **⇓⇓**); methylation of p53 consensus binding site (2, **⇓**); methylation of DNA (8, **⇓⇓**); transactivation of Ets1 binding site (2, ⇔), p53 response element (4, **⇓⇓**) | 257 **⇓** |
| Connective Tissue Develop & Function | Growth of fibroblasts (45, **⇓**); quantity of fibroblasts (7, **↓**); branching of fibroblasts (3, ⇔); morphology of fibroblasts (15, **↓**); colony formation of fibroblasts (18, **↓**); entry into M phase of fibroblasts (2, **⇓**); entry into anaphase of fibroblasts (2, **↑**); survival of fibroblasts (12, **⇓⇓**) | 111 **⇓** |
| Cell Morphology | Morphogenesis (16, **⇓**), epithelial cells (4, **⇓⇓**); transformation of cells (68, **⇓**), lymphocytes (8, **↓**), endocrine cells (3, **↑**); fibroblasts (23, **↓**); branching of cells (10, **↓**); blebbing (10, **↑**); vacuolation of cytoplasm (3, **⇓**); keratanization (4, **↑**); deformation of red blood cells (2, **⇑**); volume of nucleus (2, **⇑**); elongation of axons (6, **↓**) | 123 **⇓** |
| Imm. & Lymphatic Syst Dev & Funct | Development of thymocytes (16, **⇓**), natural killer cells (5, **⇑**); hyperactivation of T lymphocytes (4, **⇓**); colony formation of leukocyte (8, **⇑**); accumulation of monocytes (2, **⇓**); proliferation of lymphocytes (71, **↑**); adhesion of mast cells (5, **⇓**) | 95 ⇔ |
| Cell compromise | Oxidative stress response (11, **⇓**); instability of genomes (5, **↑**); deformation of red blood cells (2, **⇓**) | 22 **⇓** |
| Card sys D & F | Development blood vessel (68, **↓**); angiogenesis (50, **↓**); cardiovascular process of blood vessel (16, ⇔); morphology of blood vessel (13, **⇓⇓**) | 98 **↓** |
| Organismal dev | Development of mammalian (32, **⇓**); tolerance of mammalian (3, **↓**) | 99 **⇓** |
| Tissue dev | Developmental process of skeletal muscle (9, **⇓**), endoderm (8, **↓**), ectoderm (4, **⇓**); accumulation of monocytes (2, **⇓**) | 40 **⇓** |
| Tissue morphology | Quantity of fibroblasts (7, ⇔), neuronal progenitor cells (3, **⇓**); degeneration of skeletal muscle (3, **↑**); disorganization of lymphoid tissue (2, **⇑**) | 26 **↓** |
| DNA rep rec rep | Modification of DNA (39, **⇓⇓**); DNA damage response of cells (18, ⇔); checkpoint control (13, **⇓⇓**); conformational modification of DNA (10, **⇓⇓**); formation of mitotic spindle (9, **⇓⇓**), spindle fiber (9, **⇓⇓**); alkylation of DNA (8, **⇓⇓**); methylation of DNA (8, **⇓⇓**); moiety attachment of DNA (8, **⇓⇓**); initiation of DNA replication (7, **⇓⇓**); instability of genomes (6, **↑**); relaxation of DNA (4, **⇓⇓**); conformational change of DNA (3, **⇓**); loss of chromosomes (3, **↓**); recombination of chromosomes (3,** ↑**); assembly of nuclear envelope (2, **↓**); replication of DNA lesion (2, **⇓**) | 72 **⇓⇓** |
| RNA post-trans mod | Modification of RNA (28, **⇓⇓**); mRNA (25, **⇓⇓**); processing of RNA (24, **⇓⇓**); splicing of RNA (20, **⇓⇓**); binding of RNA (6, **⇑**); modification of tRNA (4, ⇔) | 51 **⇓⇓** |
| Carbohydrate metabolism | Biosynthesis of carbohydrate (16, **⇑⇑**); exchange of carbohydrate (3, **⇑**); biosynthesis of lactose (2, **⇑**); import of UDP-galactose (2, **⇑**) | 18 **⇑⇑** |
| Lipid metabolism | Biosynthesis of lipid (24, **⇑⇑**); quantity of fatty acid (20, **⇑**), glycolipid (12, **⇑⇑**), fat (5, **⇑⇑**), palmitoleic acid (2, **⇑**), retinyl ester (2, **⇑**); transport of fatty acid (11, **⇑⇑**), oleic acid (4, ⇔); uptake of fatty acid (7, **⇑⇑**), oleic acid (3, **↑**); incorporation of oleic acid (4, **⇑**); synthesis of diacylglycerol (4, **⇑**), galactosylceramide (3, **⇑**); formation of triacylglycerol (3, **⇑**); internalization of fatty acid (3, **↓**); clevage of glycolipid (2, **⇑**) | 61 **⇑⇑** |
| Mol biochem | Moiety attachment of amino acids (67, ⇔); binding of hormone (6, **↓**); hydroxylation of Pro (3, **↑**); O-glycosylation of protein (3, **↓**) | 132 **⇑** |
| Hem sys D & F | Proliferation of lymphocytes (71, ⇔); colony formation of leukocytes (8,** ↑** ); movement of natural killer cells (7, **↓**); development of natural killer cells (5, **⇑⇑**); hyperactivation of T lymphocytes (4, **⇓**); accumulation of monocytes (2, **⇓**) | 95 ⇔ |
| Cell assembly & org | Formation of focal adhesion (22, ⇔); elongation of axons (6, **↓**); missegregation of chromosomes (4, **↑**); activation of plasma membrane projection (3, **⇓**); exocytosis of secretory granules (3, **↓**); formation of endosome (3, **↓**); assembly of ribosome (2, **⇓**); quantity of adherens junction (2, **⇓**); volume of nucleus (2, **⇑**) | 65 **↓** |
| Cell-cell sign & inter | Contact growth inhibition (25, **⇓⇓**); recruitment of antigen presenting cells (12, **↑**); long-term potentiation of synapse (6, ⇔); attraction of granulocytes (5, **⇓**); recruitment of dendritic cells (4, **⇑**); growth of intercellular junctions (3, **↑**) | 69 **↓** |
| Immune response | Proliferation of lymphocytes (71, ⇔); recruitment of antigen presenting cells (12, ⇔) ; cell movement of natural killer cells (7, **↓**); hyperactivation of T lymphocytes (4, **⇓**); recruitment of dendritic cells (4, **⇑**); accumulation of monocytes (2, **⇓**) | 85 ⇔ |
| Molecular transport | Transport of protein (36, **↓**) | 86 **⇑** |
| Cellular movement | Chemotaxis (27, **⇑**); migration of fibroblasts (17, ⇔); invasion of fibroblasts (10, **⇓**); delay in cytokinesis (3, **↑**); infiltration of microglia (2, **⇓**) | 58 **↑** |
| Cell funct & maint | Cytostasis (45, **⇓**); ER stress response (14, **⇓**) | 64 **⇓** |
| Amino acid met | Moiety attachment of amino acids (67, ⇔); hydroxylation of Pro (3, **↑**) | 67 ⇔ |
| Cell signaling | Acetylcholine receptor signaling, muscarin pathway (3, **↓**); activation of MAPKKK (3, **↑**) | 6 ⇔ |
| Post-transl mod | Moiety attachment amino acids (67, ⇔); phosphorylation of protein fragment (7, ⇔); hydroxylation of Pro (3, **↑**); O-glycosylation protein (3, **↓**) | 71 ⇔ |
| End Sys D & F | Binding of hormone (6, **↓**) | 9 **↓** |
| Skel Musc D & F | Relaxation of vascular smooth muscle cells (2, ⇔) | 17 ⇔ |
| Protein synthesis | Release of protein (8, **↓**) | 8 **↓** |
| Nerv Sys D & F | Elongation of axons (6, **⇓**); long-term potentiation of synapse (6, **↑**); quantity of neuronal progenitor cells (3, **⇓**); infiltration of microglia (2, ⇔) | 16 **↓** |
| Nucleic acid met | Exchange of AMP, CMP-sialic acid, UDP-galactose, UMP (2, ⇔); import and transport of galactose (2, **⇑**) | 3 **↑** |
| Vit & Mineral met | Quantity of retinyl ester (2, **⇓**) | 2 **⇓** |
| Protein trafficking | Transport of protein (36, **↓**) | 36 **↓** |

Organ morphology, Organ development, Organismal survival were not considered because they were not pertinent to mammary gland

**TABLE R13**. Tabulated results from Ingenuity Pathway Analysis® (IPA) at **15 vs. 1d**. Reported are the functions with an Exact Fisher test P-value ≤ 0.05 sorted by decrease in significance. The category denotes the main functional category assigned by IPA. The functional annotation is derived by the “effect on function” in IPA. In parenthesis are reported the number of DEG for each specific function and the arrows denote the overall effect on the function inferred by the gene annotation using IPA (⇑⇑ = highly activated; ⇑ = activated; 🡩 = tend to be activated; ⇓⇓ = highly inhibited; ⇓ = inhibited; 🡫 = tend to be inhibited). For detailed explanation see Additional file 2. Some annotations on functions are common between categories and were not repeated and the overall effect takes into consideration common functions among categories. Some categories do not make biological sense based on mammary gland features (see Additional file 2 for details explanation). For those two reasons the sum of the numbers in parenthesis very often does not correspond with the total DEG in the left column (DEG)

| **Category** | **Function Annotation** | **DEG** |
| --- | --- | --- |
| Cell Death | Cell death (227, ⇔), neurons (39, **↑**), epithelial cell lines (20, **↑**), endothelial cell lines (14, **⇓**), endocrine cells (10, ⇔); survival of cells (77, **↑**), neurons (17, **↑**); Inhibition of apoptosis (32, **↑**); lysis of cells (8, ⇔); apoptosis of effector T lymphocytes (4, ⇔); activation-induced cell death of natural killer T lymphocytes (2, **⇑**) | 254 ⇔ |
| Gene Expression | Expression of synthetic promoter (47, **↑**); activation of Smad3-Smad4 binding element (8, **↑**), E box motif (7,** **⇔), retinoic acid response element (5, ⇔),TGF beta response element (5, **⇑**), AP1 consensus site (5, **⇓**), CaGA box (3,** ↑**), Pu.1 binding site (3, **↑**); transcription of TGF beta response element (5, **⇑**), CBF1 binding site (2, **⇑**); binding of Smad binding sequence (4, **⇑⇑**), cytokine response element (2, **⇑**) | 58 **↑** |
| Cell Morphology | Transformation of cells (58, **↑**); morphology of cells (56, **⇑**), muscle cells (6, **⇑⇑**), myofiber (3, **↑**); morphogenesis of cells (54, **⇑**); blebbing (9, **⇓**); branching of neurites (9, **↑**), cells (8, ⇔), fibroblasts (2, **⇑**); extension of axons (7, **↓**); cellularity (6, ⇔); vacuolation of cytoplasm (4, **↑**); compaction of cells (3, **↑**); transmembrane potential of mitochondrial (3, **↑**); permeabilization of vesicles (2, **⇓**) | 146 **⇑** |
| Cell assembly & organization | Formation of actin stress fiber (21,** ⇔**); rearrangement of cytoskeleton (12, **⇑**); quantity of actin stress fiber (9, **⇓**), focal adhesion (7, **↓**); biogenesis of cellular membrane (3, **↓**); cohesion of sister chromatids (3, **↑**); fusion of late endosome (3, **↑**), endocytotic vesicle (2, **⇑**); induction of filaments (3, **⇑**); rearrangement of actin stress fiber (3, **⇑**); disruption of lipid bilayer (2, **⇓**); | 62 **↑** |
| Card sys D & F | Development of blood vessels (50, **⇑**); cardiovascular process of organism (16, **⇑**), tissue (15, **⇓**); angiogenesis of organism (14, **↑**), artery (3, **↑**); formation of blood vessel (10, **⇑**); morphogenesis of blood vessel (8, **⇓**); development of vasculature (6, ⇔); vasculogenesis of blood vessel (3, **↑**) | 53 **⇑** |
| Cellular movement | Movement of cells (113, **↑**), mononuclear cells (6, ⇔), dermal fibroblasts (3, **↑**); migration (110, **↑**), epithelial cells (14, **⇓**), monocytes (11, **⇑**); infiltration of mononuclear leukocytes (14, **⇑**), monocytes (4, **↑**); chemotaxis of neuroglia (6, **⇑**), microglia (5, **⇑**), mononuclear cells (4, **↑**), neurons (4, **⇑**), peripheral blood leukocytes (4, **⇑⇑**), dermal fibroblasts (2, **⇑**); locomotion of cells (3, **⇑**) | 127 **⇑** |
| Cell growth & prol | Proliferation (192, ⇔), stem cells (13, **⇑⇑**), pericytes (5, **⇑**), nervous tissue cells (3, **↑**), mesenchymal stem cells (3, **⇑**); growth (163, ⇔), epithelial cells (12, **⇓**), epidermal cells (8, **⇓**), keratanocytes (7, **⇓**), pre-B lymphocytes (3, **↓**); expansion (5, **⇑⇑**), leukocytes (3, **⇑**) | 258 **↑** |
| Molecular transp | Efflux of cholesterol (9, **↓**); release of protein (8, **↓**); quantity of superoxide (7, **⇓**), lysophospholipids (4, **⇑**), sodium (4, **⇓**); removal of superoxide (3, **⇑**); accumulation of anion (4, ⇔); clearance of lipid (4, **↓**); transport of acidic amino acid (4, ⇔), CMP-sialic acid (2, **⇑**); exchange of AMP, UMP, and UDP-galactose (2, **⇑**); pinocytosis of protein (2, **⇑**); redistribution of phosphatidylinositol (2, **⇑**); uptake of Arg (2, **⇑**), Asp (2, **⇑**) | 41 ⇔ |
| Protein synthesis | Release of protein (8, **↓**) | 8 **↓** |
| Nerv sys D & F | Differentiation of astrocytes (6, **↑**); quantity of sensory neurons (5, **↓**); discharge of cells (3, **⇓**); cell-cell contact of neurons (2, **⇑**); cytostasis of astrocytes (2, **⇑**) | 43 **↑** |
| Cell-cell sign & inter | Response of cells (35, ⇔), lymphocytes (11, **↓**), T lymphocytes (10, **↓**), Th2 cells (4, **↓**), fibroblasts (3, **⇓**); adhesion of cells (35, **↑**), fibroblasts (8, **⇑**); contact growth inhibitor (17, **↑**), epithelial cells (3, **⇓**); fusion of cells (12, ⇔), muscle cells (4, **⇓**); cell-cell contact (4, **↑**) | 84 ⇔ |
| Cellular dev | Differentiation of cells (124,** ↑**), epithelial cells (24, **⇑**), antigen presenting cells (15, **↑**), fibroblasts (15, **⇓**), phagocytes (14, **↑**), stem cells (13, **⇑**), astrocytes (6,** ↑**), mesenchymal cells (6, **↑**), stromal cells (3, **⇓**), adipose stromal cells (2, **⇓**); morphogenesis of cells (54, **⇑**); developmental process of fibroblasts (36, ⇔), epithelial cells (32, ⇔), bone marrow cells (25, **↑**), stem cells (21, **↑**), keratanocytes (13, **⇑**), oligodendrocytes (7, ⇔), bone marrow-derived macrophages (6, **⇓**), neural stem cells (5, **⇑**), glomerular cells (2, **⇑**); maturation of cells (30, **⇑**); adipogenesis of fibroblasts (7, **↑**); epithelial-mesenchymal transition (7, **⇑⇑**); arrest in developmental process of leukocytes (4, ⇔); onset of differentiation of connective tissue cells (2, **⇑**) | 183 **↑** |
| Repr sys D & F | Growth of breast cell lines (⇔); movement of breast cell lines (⇔); involution of mammary gland (5, **↑**) | 24 **↓** |
| Sk & muscle D & F | Proliferation of muscle cells (23, **⇑⇑**); contraction of smooth muscle (8, **↓**); morphology of muscle cells (6, **↓↓**); morphology of myofiber (3, **↑**) | 54 **⇑** |
| Organismal dev | Developmental process of organism (94, **↑**); development of organism (80, **↑**) | 95 **↑** |
| Cell cycle | Cell cycle progression (38, **↑**); cell division (3, **⇑**); interphase of keratanocytes (3, **⇑**); G2/M phase of fibroblasts (2, **⇑**) | 46 **↑** |
| Conn tissue D & F | Differentiation of fibroblasts (15, **⇓**), stromal cells (3, **⇓⇓**), adipose stromal cells (2, **⇓**); adhesion of fibroblasts (8, **⇑**), bone marrow stromal cells (2, **⇑**); adipogenesis of fibroblasts (7, **↑**); proliferation of pericytes (5, **⇑**); cell movement of dermal fibroblasts (3, **↑**); response of fibroblasts (3, **⇓⇓**); branching of fibroblasts (2, **⇑**); chemotaxis of dermal fibroblasts (2, **⇑**); G2/M phase of fibroblasts (2, **⇑**); mineralization of fibroblasts (2, ⇔) | 49 ⇔ |
| Cell funct & maint | Cytostasis (22, ⇔); homeostasis of T lymphocytes (4, ⇔); switching of cells (2, **⇑**) | 34 **↑** |
| Tissue morphology | Remodeling of tissue (15, **⇑**); dilation of tissue (3, **↓**); structural integrity of basement membrane (2, **⇓**) | 48 **↑** |
| Tissue development | Developmental process of tissue (88, **⇑**), muscle (30, **⇑**), mesoderm (8, **⇑**), endoderm (6, **⇑**); formation of tissue (33, **⇑**); accumulation of cells (30, **⇑**), leukocytes (20, **⇑**), lymphatic system cells (10, ⇔); morphogenesis of tissue (15, **⇑⇑**); clustering of dendritic cells (4, **↑**) | 115 **⇑** |
| Hem Sys D & F | Accumulation of blood cells (20, **⇑**), leukocytes (19, **⇑**), monocytes (2, **⇑**); differentiation of phagocytes (14, **↑**), bone marrow-derived macrophages (4, ⇔); infiltration of mononuclear leukocytes (14, **⇑**), monocytes (4, **↑**); response of lymphocytes (11, ⇔), T lymphocytes (10, ⇔), Th2 cells (4, **↓**); cell movement of mononuclear cells (6, ⇔); chemotaxis of mononuclear cells (4, **⇑**); homeostasis of T lymphocytes (4, ⇔); expansion of leukocytes cells (3, **↑**); growth of pre-B lymphocytes (3, **↓**); suppression of Th2 cells (2, **⇑**) | 51 **⇑** |
| Im & Lym S D & F | Accumulation of leukocytes (19, **⇑**), monocytes (2, **⇑**); infiltration of mononuclear leukocytes (14, **⇑**), monocytes (4, ⇔); migration of monocytes (11, **⇑**); cell movement of mononuclear cells (6, ⇔); chemotaxis of peripheral leykocytes (4, **⇑⇑**), mononuclear cells (4, **⇑**) | 32 **⇑** |
| Free radical scav | Quantity of superoxide (7, **⇓**); removal of superoxide (3, **⇑**) | 9 **⇑** |
| Hair & skin D & F | Growth keratanocytes (7, **⇓**); contact growth inhibition epithelial cells (3, **⇓**); chemotaxis dermal fibroblasts (2, **⇑**); migration melanocytes (2, **⇓**) | 14 **⇓** |
| Organ developm | Formation of organ (12, ⇔) | 21 ⇔ |
| Lipid metabolism | Efflux of cholesterol (9, **↓**); production of acylglycerol (6, **⇓**); hydrolysis of phosphatidylcholine (5, **↑**), phosphatidylethanolamine (3, **↓**), phosphatidylglycerol (2, **⇓**); synthesis of triacylglycerol (5, **↑**), diacylglicerol (3, **↑**); clearance of lipid (4, **↑**); quantity of lysophospholipid (4,** ⇑**); redistribution of phosphatidylinositol (2, **⇑**) | 23 ⇔ |
| Mol biochem | Synthesis of glycosaminoglycan (6, **↓**); production of nitrite (5, **↓**), proteoglycan (3, **↑**); consumption of D-glucose (3, **⇓**); cleavage of heme (3, **⇓**); nitration of Tyr (2, **⇓**); oxidation of heme (2, **⇓**) | 46 **⇓** |
| Carbohydrate met | Synthesis of polysaccharide (15, ⇔); production of polysaccharide (5, **↑**); glycogenolysis (3, **↑**) | 30 ⇔ |
| Cell signaling | Acetylcholine receptor signaling, muscarinic pathway (3, **↑**) | 3 **↑** |
| Organ morphology | Involution of mammary gland (5,** ⇓**) | 19 **⇓** |
| Amino acid met | Transport of acidic amino acid (4, ⇔); nitration of Tyr (2, ⇔); uptake of Arg (2, **⇑**), Asp (2, **⇑**), Ser (2, **↑**) | 8 **↑** |
| Cell compromise | Damage of cells (12, **⇓**), mitochondria (5, **↓**); injury of cells (7, **⇓**); fragmentation of nucleus (6, **⇑**), mitochondria (3, **↓**); degradation of cells (3, **⇑**); disruption of lipid bilayer (2, **⇓**); toxicity of cells (2, **⇑**) | 22 **↓** |
| Drug metabolism | Oxidation of dopamine (2, ⇔); transport of drug (2, ⇔) | 4 ⇔ |
| Nucleic acid met | Exchange of AMP, CMP-sialic acid, UDP-galactose, UMP (2, **⇑**); transport of CMP-sialic acid, UMP (2, **⇑**) | 2 **⇑** |
| Post-transl mod | Nitration of Tyr (2, ⇔); | 2 ⇔ |
| Protein trafficking | Pinocytosis of protein (2, **⇑**); redistribution of F-actin (2, ⇔) | 4 **↑** |
| Organism function | Recovery (4, **↑**) | 4 **↑** |
| RNA posttrans mod | Metabolism of RNA (4, **⇑**); processing of RNA (3, **↓**); | 8 ⇔ |
| Immune response | Growth of pre-B lymphocytes (3, **⇓**) | 6 **⇓** |
| RNA damage & rep | Metabolism of RNA (4, **⇑**); editing of mRNA (2, ⇔) | 6 **↑** |
| Organismal surv | Death of animal (91, **↑**) | 91 **↑** |
| DNA rep rec rep | Oxidation of DNA (2, **⇓**); morphology of nuclear bodies (2, ⇔) | 4 **↑** |

Endocrine system development and function was not considered because it was not pertinent to mammary gland

**TABLE R14**. Tabulated results from Ingenuity Pathway Analysis® (IPA) at **30 vs. 15d**. Reported are the functions with an Exact Fisher test P-value ≤ 0.05 sorted by decrease in significance. The category denotes the main functional category assigned by IPA. The functional annotation is derived by the “effect on function” in IPA. In parenthesis are reported the number of DEG for each specific function and the arrows denote the overall effect on the function inferred by the gene annotation using IPA (⇑⇑ = highly activated; ⇑ = activated; 🡩 = tend to be activated; ⇓⇓ = highly inhibited; ⇓ = inhibited; 🡫 = tend to be inhibited). For detailed explanation see Additional file 2. Some annotations on functions are common between categories and were not repeated and the overall effect takes into consideration common functions among categories. Some categories do not make biological sense based on mammary gland features (see Additional file 2 for details explanation). For those two reasons the sum of the numbers in parenthesis very often does not correspond with the total DEG in the left column (DEG)

| **Category** | **Function Annotation** | **DEG** |
| --- | --- | --- |
| Cell Morphology | Morphology of leukocytes (4, **⇑**), phagocytes (3, **↑**), nucleus (3, **⇑⇑**); transformation of dendritic cells (2, **⇑**); depolarization of mitochondria (3, **⇑⇑**); mass of cells (3, **↓**); modification of cells (4, **↓**); blebbing (3, **↓**) | 17 **⇑** |
| Nervous System Dev. and Funct. | Neurological process (9, **⇑**); axonogenesis (2, **⇑**); long-term potentiation (6, **⇑**); synaptic transmission (7, **⇑⇑**) | 19 **⇑** |
| DNA Replic. Recomb. & Repair | Breakage of DNA (4, **⇑**), damage of DNA (5, **⇑**); cleavage of DNA fragment (2, **⇑**); elongation of DNA (2, **↑**) | 10 **⇑** |
| Cell Compromise | Oxidative stress response of epithelial (2, **⇑**); condensation of mitochondria (2, **⇑**); disruption of plasma membrane (2, **⇑**) | 6 **⇑** |
| Lipid metabolism | Uptake of taurocholic acid (2, **⇑**); | 7 **↑** |
| Small Molecule Biochemistry**^1^** | phosphorylation of amino acids (12, ⇔), tyrosine (5, ⇔); Import of D-glucose (3, **⇓⇓**); uptake of thymidine (3, **⇓**), L-glutamic acid (2, **⇓**), taurocholic acid (2, **⇑**); release of glutamic acid (3, **⇑⇑**); quantity of nitrite (2, **⇑**) | 24 ⇔ |
| Cellular Growth and Proliferation | Quantity of fibroblasts (4,** ↑**); growth of cells (39, **↓**), fibroblasts (10, **↑**), epithelial (4, **↑**), smooth muscle cells (3, **↓**); proliferation of macrophages (3, **↓**), B lymphocytes (7, **↑**), lymphatic system cells (14, **↓**); formation of cells (10, **↓**), multinucleated cells (3, **⇓**); stimulation of cell (6, **⇑**), muscle cells (2, **↑**), fibroblasts (4, **↑**) | 48 **↑** |
| Conn Tissue D. & F. | Quantity of fibroblasts (4, **↑**); cell viability of fibroblasts (3, **↓**) | 17 **↑** |
| Gene Expression | Activation of Ets1 binding site (2, **⇑**), NFAT response element (3, **⇓**), T-cell factor responsive element (2, **⇑**); binding of C/ebp beta binding site (2, **↓**), Sp1 binding site (3, **⇓**), Sis-inducible element (2, **⇑**); expression of DNA endogenous promoter (3, **↓**); transcription (34, **↓**) | 36 **↓** |
| Cell Cycle | Arrest in G0 phase (5, ⇔); arrest in interphase of muscle cells (2, **⇓**); interphase of cells (13, **↓**), stem cells (2, **⇓**); arrest in cell division process of muscle cells (2, **⇓**); cell division process of DNA (3, **⇓⇓**), chromosomes (2, **⇑**); arrest in cell stage (10, **↑**); G2/M phase transition (3, **↓**); senescence of fibroblast cells (3, **↓**) | 18 **⇓** |
| Immune Response | Antiviral response (5, **⇑⇑**); cytotoxic T lymphocyte response (2, **↑**); activation of leukocyte (3, **↑**); secretion of cytokine (2, **⇑**); response of neutrophils (2, **⇑**) | 14 **⇑** |
| Cellular Development | Developmental of fibroblasts (10, **↑**), T lymphocytes (10, ⇔), muscle cells (8, **↓**); angiogenesis of endothelial cells (3, **↑**); maturation of dendritic cells (4, ⇔); development of macrophages (3, ⇔) | 31 **↑** |
| Cellular Movement | Migration of B lymphocytes (3, **⇑**), endothelial cells (4, **⇑**), monocyte-derived dendritic cells (2, **↑**) | 13 **⇑** |
| Immune system dev & function | Proliferation of B lymphocytes (7, ⇔); macrophages (3, **↓**); priming of macrophages (2, **⇑**) | 13 ⇔ |
| Tissue Morphology | quantity of tissue (6, **⇓**), connective tissue (4, **⇓**) | 13 **⇓** |
| Organ Development | Organogenesis (15, **↑**) | 15 **↑** |
| Cell-To-Cell Sign & Interaction | neurological process of intercellular junctions (5, **⇑**); attachment of cells (6, **⇑**), endothelial cells (2, **⇑**); activation of leukocyte (3, **↑**); fusion of cells (5, **↑**); stimulation of cells (6, **↑**); neurotransmission (7, **⇑**); response of phagocytes (2, **⇑**); sensitization of cell lines (2, **⇑**) | 22 **⇑** |
| Skel Musc D & F | Growth of smooth muscle cells (2, **↓**); stimulation of muscle cells (2, ⇔) | 10 **↓** |
| Cell death | Cell death (41, ⇔), endocrine cells (4, ⇔), hematopoietic cells (3, **↑**), microvasculature endothelial cells (2, **⇑**) ; cytotoxicity of leukocytes (5, ⇔); survival of B lymphocytes (4, **⇑**); viability of fibroblasts (3, **⇑**) | 49 ⇔ |
| Card sys D & F | Angiogenesis of endothelial cells (3, **↑**); attachment of endothelial cells (2, **⇑**) | 9 **⇑** |
| Organ morphology | Thickness of organ (2, **⇓**) | 4 **⇓** |
| Hem sys D & F | Quantity of B lymphocytes (7, **↓**); survival of B lymphocytes (4,** ⇑** ); activation of leukocytes (3, **↑**); development of macrophages (3, **↓**); response of neutrophils (2, **⇑**) | 13 ⇔ |
| Tissue development | Formation of connective tissue (5, ⇔); activation of tissue (3, ⇔) | 10 ⇔ |
| Carbohydrate met | Import of carbohydrates (3, **⇓**) | 3 **⇓** |
| Mol transport | Transport of cation (8, ⇔), calcium (5, **↑**); entrance of calcium (3, **↑**); import of carbohydrates (3, **⇓**); release of Glu (3, **⇑**); uptake of thymidine (3, **⇓**); quantity of nitrite (2, **⇑**); secretion of cytokines (2, **⇑**); uptake of Glu (2, **⇓**) | 18 **↑** |
| Nucleic acid met | Metabolism of nucleic acid component or derivative (3, **↑**) | 6 ⇔ |
| Amino acid met | Phosphorylation of amino acid (12, ⇔); modification of aromatic amino acid (5, **↓**); release of α-amino acid (3, **⇑**) | 15 ⇔ |
| Cell assembly & org | Depolarization of mitochondria (3, **⇑**); morphology of nucleus (3, **⇑**); discruption of plasma membrane (2, **⇑**) | 12 **⇑** |
| Cell signaling | IκB kinase/NFκB cascade (6, **⇑**) | 16 **⇑** |
| Post-transl modific | Phosphorylation of amino acids (12, ⇔), Tyr (5, ⇔); | 14 **↓** |
| Vit & Min metab | Transport of calcium (5, **↑**); entrance of calcium (3, **↑**) | 8 **↑** |
| Protein trafficking | Secretion of cytokines (2, **⇑**) | 3 **⇑** |
| Cell Func & Maint | Contact growth inhibition of epithelial cells (2, **↓**); function of dendritic cells (2, **⇑**) | 5 ⇔ |
| Hair & skin D & F | Growth of epithelial cells (4, **⇓**) | 6 **⇓** |
| Drug metabolism | Conversion of retinoic acid (1,** ↑** [*TNF*]) | 1 **↑** |
| Organismal dev | Delay in re-epithelization (1, **↓ **[*TNF*]) | 1 **↓** |
| Protein synthesis | Aminoacylation of Trp (1, **↓ **[*WARS*]) | 1 **↓** |
| RNA damage & rep | Degradation of 18s rRNA (1,** ↑** [*TNF*]) | 1 **↑** |
| RNA post-trans mod | Annealing of RNA (1, **↑** [*EIF2AK2*]; decapping of RNA (1, **↑** [*PABPC1*]) | 2 **↑** |

Reproductive and endocrine system development and function were not considered because they were not pertinent to mammary gland

**TABLE R15**. Tabulated results from Ingenuity Pathway Analysis® (IPA) at **60 vs. 30d**. Reported are the functions with an Exact Fisher test P-value ≤ 0.05 sorted by decrease in significance. The category denotes the main functional category assigned by IPA. The functional annotation is derived by the “effect on function” in IPA. In parenthesis are reported the number of DEG for each specific function and the arrows denote the overall effect on the function inferred by the gene annotation using IPA (⇑⇑ = highly activated; ⇑ = activated; 🡩 = tend to be activated; ⇓⇓ = highly inhibited; ⇓ = inhibited; 🡫 = tend to be inhibited). For detailed explanation see Additional file 2. Some annotations on functions are common between categories and were not repeated and the overall effect takes into consideration common functions among categories. Some categories do not make biological sense based on mammary gland features (see Additional file 2 for details explanation). For those two reasons the sum of the numbers in parenthesis very often does not correspond with the total DEG in the left column (DEG)

| **Category** | **Function Annotation** | **DEG** |
| --- | --- | --- |
| Lipid Metabolism | Cleavage of lipid (4, **⇑⇑**), glycolipid (2,** ⇑**), leukotriene (2, **⇑**); accumulation of phosphatidic acid (3, **⇓⇓**), phosphatidylinositol phosphate (2, **⇓**); hydrolysis of sphingolipid (3, **↑**); storage of lipid (3, **⇓⇓**); release of leukotriene (3, **↑**); quantity of phosphatidylinositol 3,4-diphosphate (2, **⇓**); generation of ceramide (2, **⇑**) | 22 **↑** |
| Small Molecule Biochemistry | Dephosphorylation of amino acids (5, **↑**); metabolism of glutathione (4, ⇔); release of histamine (3, **⇑⇑**) | 36 **↑** |
| Cellular Compromise | Degeneration of cells (8, ⇔) | 16 ⇔ |
| Tissue morphology | Quantity of blood cells (20, **↑**), phagocytes (9, **⇓**), connective tissue cells (7, **↓**), red blood cells (4, **↓**), megakaryocyte (3,** ↓**); morphology of blood vessel (3, **↓**); volume of tissue (3, **⇓**) | 35 **↓** |
| Conn Tissue D & F | Quantity of connective tissue cells (7, **↓**) | 16 **↓** |
| Sk & muscle D & F | Differentiation of muscle cells (7, **↑**); fusion of muscle cells (2, **⇓**) | 22 ⇔ |
| Gene expression | Activation of AP1 consensus site (6, ⇔), antioxidant response element (3, **⇓**), Ets element (3, **↑**) | 27 ⇔ |
| Carbohydrate met | Accumulation of phosphatidylinositol (2, **⇓**), phosphatidic acid (3, **⇓⇓**), phosphoinositide (3, **⇓⇓**), phosphatidylinositol phosphate (2, **⇓**), | 5 **⇓** |
| Cellular development | Differentiation (21, ⇔); development of blood cells (14, **↓**), leukocytes (11, **↓**), pre-B lymphocytes (3, **↑**); developmental process of muscle cells (8, **↑**), erythroid progenitor cells (3, **↑**); arrest in development of blood cells (3, **⇓**) | 33 ⇔ |
| Drug metabolism | Metabolism of glutathione (4, ⇔); accumulation of drugs (2, **⇓**) | 10 **↑** |
| Molecular transport | Release of histamine (3, **⇑**), leukotriene (3, **↑**) | 20 ⇔ |
| Nerv sys D & F | Proliferation of neurons (5, **↓**), granule cell precursors (3, **↓**), neuronal progenitor cells (3, **↓**) | 8 **↓** |
| DNA rep rec rep | Modification of DNA (11, **↑**); double-stranded DNA break repair (5,** ⇓⇓** ) | 13 **↑** |
| Cell morphology | Transformation of cells (21, ⇔), connective tissue cells (7, **↑**); size of cells (11, **⇑**); morphology of endothelial cells (2, **⇓**) | 34 **↑** |
| Cell growth & prol | Colony formation (18, ⇔) | 27 ⇔ |
| Im & lym S D & F | Degranulation of mast cells (4, **⇑**); size of bone marrow cells (2, **⇑**) | 20 **↓** |
| Cell death | Death of blood cells (18, **⇓**), organ (5, **↑**), lymphatic system cells (3, **↓**), mast cells (3, **↓**), red blood cells (3, **↓**) | 26 **↓** |
| Cell cycle | G1 phase of blood cells (3, ⇔); cell division process of astrocytes (2, **⇓**) | 9 **↓** |
| Cell assembly & org | Accumulation of autophagic vacuoles (2, **⇓**) | 21 **↓** |
| Repr sys D & F | Lactation of mice (3, **⇓**); reproduction (3, **↓**) | 11 **↓** |
| Immune response | Antiviral response (5, **⇓⇓**); hyperactivation of T lymphocytes (2, **⇓**) | 12 **⇓** |
| Organ development | Increase development of dermis and nipple (PTHLH, **⇑**); increase formation of tectorial membrane (THRA, **⇑**) | 6 **↑** |
| Post-transl mod | Modification of protein (28, **↑**); dephosphorylation of amino acids (5, **↑**); glycosylation of protein (3, **↑**) | 28 **↑** |
| Cell-cell sig & inter | Fusion of cells (5, **↓**), muscle cells (2, **⇓**); hyperactivation of T lymphocytes (2, **⇓**) | 13 **↓** |
| Organismal survival | Death of animal (32, **↑**) | 32 **↓** |
| RNA trafficking | Localization of mRNA (2, ⇔) | 2 ⇔ |
| Card sys D & F | Morphology of blood vessel (3, **↓**), endothelial cells (2, **⇓**) | 9 **↓** |
| Organismal develop | Morphology of vessel (3, **↓**) | 3 **↓** |
| Tissue development | Growth of connective tissue (3, **↑**); delay in differentiation of sensory epithelium (1,** ↓ [***THRA*]); generation of oligodendrocytes (1,** ↓ [***FGF2*]) | 8 ⇔ |
| Free radical scav | Metabolism of superoxide (2, ⇔) | 2 ⇔ |
| RNA postransl mod | Cleavage of mRNA (2, ⇔); demethylation of RNA (1,** ↑ [***ALKBH3*]); unwinding of siRNA (1, **↓ [***DDX58*]) | 4 ⇔ |
| Amino acid metab | Dephosphorylation of amino acids (5, **↑**); metabolism of amino acid analogs (1,** ↑ [***CCBL1*]);quantity of Cys (1,** ↑ [***GGT1*]); utilization of Glu (1,** ↑ [***GGT1*]); | 7 **↑** |
| Cell signaling | Reduction of calcium (1, ,** ↑ [***PTHLH*]); tie receptor signaling pathway (1, **↓ [***TEK*]) | 2 |
| Cell function & main | Investment of smooth muscle cells (1, **↓ [***MAPK7*]); presence of membrane ruffle (1, ,** ↑ [***ABL1*]); reorganization of microfilaments (1, **↓ [***MAPK7*]) | 4 ⇔ |
| End Sys D & F | Binding of dexamethaspne (1, **↓ [***HDAC6*]); excretion of β-estradiol (1, **↑ [***ABCG2*]); quantity of dexamethasone (1, **↑ [***AHR*]) | 5 ⇔ |
| Energy production | Trapping of ATP (1, **↑ [***ABCG2*]) | 1 **↑** |
| Hair & skin D & F | Development of dermis (1, **↑ [***PTHLH*]) | 1 **↑** |
| Nucleic acid metab | Clearance of drug (1, **↑ [***ABCG2*]); incorporation of dCMP (1, **↑ [***REV1*]); transport of dADP, dATP, dCDP, deoxycytidien triphosphate, dGTP, dTTP (1, **↑ [***SLC25A19*]) | 3 **↑** |
| Organ morphology | Hypertrophy of skin (1, **↑ [***AHR↓*]); | 1 ⇔ |
| Vit & Mineral Met | Catabolism of retinoid (1, **↑ [***AHR↓*]);hydrolysis of palmitoylethanolamide (1, **↑ [***NAAA*]); reduction of calcium (1, **↑ [***PTHLH*]) | 3 **↑** |

Organismal function and Cellular movement were not considered because it was not pertinent to mammary gland

**TABLE R16**. Tabulated results from Ingenuity Pathway Analysis® (IPA) at **120 vs. 60d**. Reported are the functions with an Exact Fisher test P-value ≤ 0.05 sorted by decrease in significance. The category denotes the main functional category assigned by IPA. The functional annotation is derived by the “effect on function” in IPA. In parenthesis are reported the number of DEG for each specific function and the arrows denote the overall effect on the function inferred by the gene annotation using IPA (⇑⇑ = highly activated; ⇑ = activated; 🡩 = tend to be activated; ⇓⇓ = highly inhibited; ⇓ = inhibited; 🡫 = tend to be inhibited). For detailed explanation see Additional file 2. Some annotations on functions are common between categories and were not repeated and the overall effect takes into consideration common functions among categories. Some categories do not make biological sense based on mammary gland features (see Additional file 2 for details explanation). For those two reasons the sum of the numbers in parenthesis very often does not correspond with the total DEG in the left column (DEG)

Organ morphology and Organismal survival were not considered because it was not pertinent to mammary gland

| **Category** | **Function Annotation** | **DEG** |
| --- | --- | --- |
| Cell Death | Cell death (62, **↑**) | 63 **↑** |
| Cell Morphology | Shape change of fibroblasts (5, **⇑⇑**); cell rounding of cells (4, ⇔); cell spreading of smooth muscle cells (2, **⇑**), epithelial cells (2, **⇑**); conversion of cells (2, **⇓**), fibroblasts (2, **⇓**) | 19 **⇑** |
| Connective Tissue D & F | Growth of fibroblasts (11, **↓**); development of fibroblasts (2, **⇑**); binding of fibroblasts (3, **⇓⇓**); adipogenesis of fibroblasts (3, **⇓⇓**); adhesion of fibroblasts (3, **↑**) | 22 **↓** |
| Carbohydrate Metabolism | Quantity of carbohydrate (10, **⇓**); synthesis of polysaccharide (6, **↓**); incorporation of phosphatidylcholine (2, **⇓**); | 20** ⇓** |
| Drug Metabolism | Production of progesterone (4, **↓**) | 16 **↓** |
| Small Molecule Biochemistry | Accumulation of lipid (8, **⇓**); quantity of acylglycerol (7, **⇓⇓**), fatty acid (6, **↓**), acyl-coenzyme A (2, **↑**); production of hormone (5, ⇔); incorporation of lipid (3, **⇓⇓**), phosphatidylcholine (2, **↑**), triacylglycerol (2, **⇓**); synthesis of steroid hormone (2, **⇑**), amino acid (2, **⇑**); | 29 **⇓** |
| Cell development | Developmental process of fibroblast cells (11, ⇔); growth of fibroblast cells (11, **↓**); adipogenesis (3, **⇓**); development of fibroblasts (2, **⇑**) | 18 ⇔ |
| Cell growth & prol | Growth of cells (50, **↓**); colony formation (14, **⇓**); proliferation of multilineage progenitor cells (2, **⇓**) | 53 **↓** |
| Cell movement | Migration of T lymphocytes (5, **⇑⇑**); invasion of fibroblasts (3, **↓**) | 15 **↑** |
| Cell assembly & org | Biogenesis of organelle (7, **↓**), Golgi apparatus (2, **⇓**) | 26 **↓** |
| Immune response | Antiviral response (5, ⇔) | 8 ⇔ |
| Repr sys D & F | *Included only 1 gene per annotated function; overall there were an decrease in the function due to* **⇓** *of PRLR and FOXB1* | 9 **↓** |
| Lipid metabolism | Production of progesterone (4, **↓**); biosynthesis of steroids (2, **⇑**); incorporation of acylglycerol (2, **⇓**); oleic acid (2, **⇓**) | 22 **↓** |
| Organismal devel | Differentiation or organism (1, **↑ [***NR5A1*]); secretion of breast milk (1 ,**↓ [***PRLR↓*]) | 7 ⇔ |
| Cell-cell sign & inter | Binding of cells (17, **↓**), fibroblasts (3, **↓**); adhesion of fibroblasts (3, **↑**); detachment of cells (3, **↓**) | 24 **↓** |
| Gene expression | Activation of dioxin response element and Sf1 binding site (2, **⇑**); stimulation of cAMP response element (2, **⇑**) | 6 **⇑** |
| Molecular transport | Accumul lipid (8, **⇓);** quantity FA (6, **↓**), acylglycerol (7, **⇓⇓**), acyl-CoA (2, **↑**), phosphate (2, ⇔) | 19 **⇓** |
| Nervous Sys D & F | Development of peripheral nervous system (2, **⇑**); size of neurons (2, **↓**) | 9 ⇔ |
| End Sys D & F | Production of progesterone (4, **↓**) | 7 **↓** |
| Tissue morphology | Modification of tissue (3, **↓**); diameter of blood vessel (2, **⇑**) | 13 ⇔ |
| Cell cycle | *Included only 1 gene per annotated function; overall there were an equilibrium between induction and inhibition* | 11 ⇔ |
| Tissue development | Deposition of subcutaneous fat (1,**↓ [***SPARC↑*]); developmental of nerve process (1,**↓ [***FOXB1↓*]), vascular smooth muscle (1, ,**↓ [***JAG1↓*]); | 12 **↓** |
| Organismal function | Body temperature (2, **⇑**); delay in recovery (1,**↓ [***TEK↑*]); | 3 **↑** |
| Amino acid metab | Synthesis of amino acid (2, **⇑**), Cys (1, **↑ [***CBS↑*]), Ser (1, **↓ [***LEPR↑*]); uptake of Ser (1, **↓ [***SLC3A2↓*]); | 4 **↑** |
| Card sys D & F | Angiogenesis of capillary vessel (2, **⇑**) | 9 **⇑** |
| Cell signaling | Activation of calcium (1,**↓ [***ITPR1↓*]);tie receptor signaling pathway (1, ,**↑ [***TEK↑*]); | 2 ⇔ |
| Cell compromise | *Included only 1 gene per annotated function; overall there were an tendency to induce cell compromise* | 9 **↑** |
| Cell function & main | Cytostasis of muscle cells (1, **↑ [***MYOD1↑*]); organization of podocytes (1, **↑ [***FBLN1↑*]); reorganization of microfilaments (1, **↑ [***MGAT5***↓]**), | 6 **↑** |
| DNA rep rec & rep | Morphology of nuclear matrix (1, **↑ [***VIM↑*]) | 1 **↑** |
| Energy production | Electron transport of submitochondrial particles (1, **↑ [***STC1↑*]) | 1 **↑** |
| Hair & skin D & F | Morphology of melanocytes (1, **↑ [***SPARC↑*]) | 1 **↑** |
| Hem Sys D & F | *Included only 1 gene per annotated function; overall there were an tendency to inhibit the function* | 7 **↓** |
| Im & lym S D & F | *Included only 1 gene per annotated function; overall there were an tendency to induce immune & lymphatic system devel & function* | 5 **⇑** |
| Nucleic acid metab | Quantity of acyl-CoA (2, ⇔); *included also several annotated functions with 1 gene* | 8 **↓** |
| Organ development | Outgrowth of mammary lobuloalveaolar unit (1, **↓ [***PRLR↓*]); | 7 **↓** |
| Post-translat mod | Thiolation of protein (1, **↓ [***XDH↓*]); | 1 **↓** |
| Protein trafficking | Accumulation of protein fragment (1, **↑ [***FN1↑*]); uptake of green fluorescent protein (1, **↑ [***IGFBP3↑*]) | 2 **↑** |
| RNA postrans mod | Alternative splicing (1, **↑ [***MYOD1↑*]); decapping of RNA (1, **↓ [***PABPC1↓*]); | 2 ⇔ |
| Skel & musc D & F | Cell spreading of smooth muscle cells (2, ⇔); *included also several annotated functions with 1 gene* | 8 ⇔ |
| Vit & Mineral met | Activation of calcium (1, **↓ [***ITPR1↓*]); catabolism of retinoid (1, **↑ [***AHR↑*]);hydroxylation of 4-hydroxyretinoic acid (1, **↑ [***CYP26A1↑*]) *included also other annotated functions with 1 gene* | 3 **↑** |

**TABLE R17.** Tabulated results from Ingenuity Pathway Analysis® (IPA) at **240 vs. 120d**. Reported are the functions with an Exact Fisher test P-value ≤ 0.05 sorted by decrease in significance. The category denotes the main functional category assigned by IPA. The functional annotation is derived by the “effect on function” in IPA. In parenthesis are reported the number of DEG for each specific function and the arrows denote the overall effect on the function inferred by the gene annotation using IPA (⇑⇑ = highly activated; ⇑ = activated; 🡩 = tend to be activated; ⇓⇓ = highly inhibited; ⇓ = inhibited; 🡫 = tend to be inhibited). For detailed explanation see Additional file 2. Some annotations on functions are common between categories and were not repeated and the overall effect takes into consideration common functions among categories. Some categories do not make biological sense based on mammary gland features (see Additional file 2 for details explanation). For those two reasons the sum of the numbers in parenthesis very often does not correspond with the total DEG in the left column (DEG)

Reproductive system development and function was not considered because it was not pertinent to mammary gland

| **Category** | **Function Annotation** | **DEG** |
| --- | --- | --- |
| Cell-To-Cell Signaling and Interaction | Binding of epithelial cells (9, **⇓**), keratanocytes (4, **⇓**); adhesion of fibroblasts (9, **⇑**), epithelial cells (11, **↓**), B lymphocytes (6, **↓**), pre-B lymphocytes (4, **↑**), mesangial cells (4, **⇓**), microvascular endothelial cells (4, **↑**); fusion of muscle cells (8, **⇓**); response of antigen presenting cells (6, **↓**); signaling (6, **↓**); dynamics of focal adhesions (2, **⇓**); cell-cell contacts (3, **↓**); attachment of endothelial cell lines (3, **↑**); hyperactivation of T lymphocytes (3, **↓**); recruitment of mononuclear cells (3, **↑**) | 64 **↓** |
| Cardiovascular Syst Dev & Function | Development of endothelial cells (14,** ↓**); angiogenesis (12, ⇔); adhesion of microvascular endothelial cells (4, ⇔); binding of high endothelial postcapillary venule (3, **↓**) | 32 **↓** |
| Cellular Development | Differentiation of epithelial cells (30, **↓**), epidermal cells (18, ⇔), keratanocytes (14, **↑**); developmental process of endothelial cells (23, **↓**), epidermal cells (22, **↑**), myoblasts (13, **⇑**) pre-B lymphocytes (9, **⇑**), neuronal progenitor cells (6, **⇑**),microvascular endothelial cells (4, **↓**), neural precursor cells (4, **↓**); dedifferentiation of cells (8, **↓**); regeneration of muscle cells (4, **⇑⇑**); growth of stromal cells (3, **↑**); maturation of myeloid dendritic cells (3, **↓**); morphogenesis of epithelial cells (3, **↑**) | 108 **↑** |
| Cell death | Cell death of fibroblast (51, **↑**), sensory neurons (7, **↑**), peripheral blood cells (4, **↓**); inhibition of apoptosis (35, **⇑**); viability of blood cells (8, ⇔), lymphocytes (5, **↓**); apoptosis of peripheral blood cells (7, **↑**); regeneration of muscle cells (4, **⇑⇑**) | 113 **↓** |
| Gene expression | Inhibition of genes (17, **↑**); localization of mRNA (5, **↑**); recruitment of mRNA (4, ⇔) | 26 **↓** |
| Molecular transport | Concentration of calcium (14, **↓**); accumulation of reactive oxygen species (6, **⇑**); quantity of sphingomyelin (6, **⇓**); release of superoxide (6, **↓**); quantity of malonyl-CoA (5, **⇓**); exocytosis of proteins (4, **↓**); release of neurotransmitter (3, **⇓**); transport of lactate (3, **⇓**) | 55 **↓** |
| RNA trafficking | Localization of mRNA (5, **↑**); recruitment of mRNA (4, ⇔); export of RNA (3, **↑**) | 11 **↑** |
| RNA post-trans mod | Modification of RNA fragment (4, **⇑⇑**) | 4 **⇑⇑** |
| Lipid metabolism | Exposure of phospholipid (6, **⇑**), phosphatidylserine (5, **⇑**); quantity of Acyl-CoA (5, **⇓**), malonyl-CoA (5, **⇓**); synthesis of phosphatidylcholine (5, ⇔), diacylglycerol (4, ⇔); generation of ceramide (4, **⇓⇓**), ganglioside GD3 (2, **⇓**); production of 4-androstene-3,17-dione (4, **⇓⇓**); binding of βestradiol (3, **↓**); desaturation of fatty acid (3, **↓**); distribution of glycolipid (3, **↓**); metabolism of acetyl-CoA (3, **↓**); cleavage of glycolipid (2, **⇓**); hydrolysis of phosphatidylserine (2, **⇓**); targeting of ganglioside GD3 (2, **⇓**) | 40 **↓** |
| Nucleic acid metab | Binding of ATP (4, **⇑**); metabolism of S-adenosylmethionine (3, **⇓**); depletion of S-adenosylmethionine (2, **⇓**) | 14 ⇔ |
| Small molecular biochem | Homeostasis of calcium (21, **⇑⇑**), metal ion (5, **⇑⇑**), Cu^2+^ (3, **⇑⇑**); binding of hormone (6, **⇑⇑**); metabolic process of polyols (6, **⇓**); oscillation of calcium (6, ⇔); formation of anion (4, **↓**), hydrogen peroxide (4, **↓**); hydroxylation of L-amino acid (3, **⇓**); metabolism of N-acetylglucosamine (3, **↑**); release of neurotransmitter (3, **⇓**); degradation of L-dopa (2, **⇓**); oxidation of anion and nitrite (2, **⇓**) | 93 **↑** |
| Tissue development | Adhesion of epithelial cells (11, **↓**), extracellular matrix (7, ⇔), B lymphocytes (6, **↓**), mesangial cells (4, **⇓**), microvascular endothelial cells (4, ⇔), pre-B lymphocytes (4, ⇔) | 25 **↓** |
| Cell assembly & org | Organization of cytoskeleton (23, ⇔); remodeling of chromatin (13, **↑**); transport of Golgi vesicles (10, **⇑**); disruption of filaments (7, ⇔); biogenesis of lysosome (6, **⇑**); formation of tubules (6, **⇑**); elongation of filaments (4, **⇑**); formation of actin ring (4, **⇑**); morphology of filaments (4, **⇑**); nucleation of actin (4, **⇑⇑**); remodeling of nucleosomes (4, **↓**); disruption of plasma membrane (3, **↓**); formation of cell-cell contact (3, ⇔), hemidesmosomes (3, ⇔), membrane blebs (3, **↑**); fusion of lysosome (3, **⇑**); quantity of lysosome (3, **⇑**); reorganization of microtubules (3, **↓**); assembly of ribosome (2, **⇑**); fusion of endocytotic vesicle (2, **⇑**); remodeling of axons (2, **⇓**); structural integrity of plasma membrane (2, **⇑**); translocation of microtubule organizing centers (2, **⇓**) | 98 **⇑** |
| Hair & skin D & F | Differentiation of epidermal cells (18, ⇔) | 32 ⇔ |
| Energy production | Binding of ATP (4, **⇑**) | 5 **⇑** |
| Cellular movement | Movement of macrophages (24, ⇔), monocytes (17, ⇔); migration of epithelial cells (16, ⇔), epidermal cells (9, ⇔), memory T lymphocytes (4, ⇔), peripheral blood lymphocytes (4, **⇑**) | 58 ⇔ |
| Hem Sys D & F | Movement of macrophages (24, ⇔), monocytes (17, ⇔); adhesion of B lymphocytes (6, **↓**); migration of memory T lymphocytes (4, ⇔); hyperactivation of T lymphocytes (3, ⇔); recruitment of mononuclear cells (3, **↑**); formation of multinucleated giant cells (2, **⇓**) | 39 **↓** |
| Immune response | Inflammation of the organism (16, **↑**); antibacterial response of organism (9, **⇓**); response of dendritic cells (5, **↓**); biosynthesis of cytokine (3, **⇓**); cytotoxic T lymphocyte response (3, **↑**); hypeactivation of T lymphocytes (3, **↓**); recruitment of mononuclear cells (3, **↑**) | 49 ⇔ |
| Cell compromise | Dysfunction of cells (8, **⇓**); stress response of cells (4, **↓**) | 29 **↓** |
| Cell morphology | Morphogenesis (12, **⇑**), epithelial cells (3, **↑**); branching of cells (5, **↑**); polarization of phagocytes (5, **↑**); branching morphogenesis (4, **↓**); transformation of cells (4, **↓**); transformation of lymphatic system cells (3, **↓**); length of mitochondria (2, **⇑**) | 51 **↑** |
| Cell funct & mainten | Exocytosis of proteins (4, ⇔); authophagy of epithelial cells (3, **↓**) | 43 ⇔ |
| Imm Sys D & F | Adhesion of B lymphocytes (6, **⇓**); hyperactivation of T lymphocytes (3, ⇔); formation of multinucleated giant cells (2, **⇓**) | 11 **⇓** |
| Nerv sys D & F | Neurological process of cells (29, ⇔); long-term potentiation of cells (10, **↓**); proliferation of neuronal progenitor cells (5, **⇑**) | 51 ⇔ |
| Organ development | Remodeling of organ (4, **↓**) | 11 **↓** |
| Organ morphology | Healing of organ (4, **↑**) | 9 **↑** |
| Organismal function | Healing of organ (4, **↑**) | 4 **↑** |
| Protein trafficking | Exocytosis of protein (4, **↓**); localization of green fluorescent protein (3, **⇑⇑**) | 7 ⇔ |
| Tissue morphology | Fusion of tissue (7, **↓**); hypoplasia of muscle (5, **↑**); quantity of fibroblasts (5, ⇔) | 24 ⇔ |
| Cell cycle | Arrest in cell cycle progression (46, **↑**); modification of chromatin (16, **⇑**); senescence (16, ⇔); arrest in G1/S phase transition (9, **↓**); entry into cell division process of fibroblasts (7, **↑**); re-entry into cell cycle progression (7, **↓**); sub-G1 phase (6, **↓**); interphase of B lymphocytes (4, **⇑**); termination of cell cycle progression (4, **↓**); exit from S-phase (3, **⇓**); mitosis of connective tissue cells (3, **↓**); entry into S-phase of cortical neurons (2, **⇑**) | 85 **↓** |
| DNA rep rec & rep | Modification of chromatin (16, **⇑**); remodeling of chromatin (13, **⇑**), nucleosomes (4, **↓**); replication of genomic DNA (2, ⇔) | 22 **⇑** |
| Carbohydrate met | Metabolic process of polyols (6, **⇓**); exposure of phospholipid (6, **⇑**), phosphatidylserine (5, **⇑**); synthesis of phosphatidylcholine (5, ⇔), metabolism of glycerol (4, **↓**), polyols (4, **↓**), N-acetylglucosamine (3, **↑**); hydrolysis of phosphatidylserine (2, **⇓**); inactivation of platelet activating factor (2, ⇔) | 20 ⇔ |
| Cell signaling | Concentration of calcium (14, **↓**); stabilization of calcium (2, **⇑**) | 20 ⇔ |
| Cell growth & prol | Proliferation of fibroblasts (36, **↓**), pericytes (6, **↓**), myoblasts (5,**↓** ); outgrowth of cells (6, ⇔); growth of stromal cells (3, **↑**) | 64 **↓** |
| Vit & Mineral met | Concentration calcium (14, **↓**); oscillation calcium (6, ⇔); homeostasis of Ca (3, **⇑⇑**); stabilization calcium (2, **⇑**); stimulation calcium (2, ⇔) | 20 **↓** |
| End Sys D & F | Binding of hormone (6, **⇑⇑**), β-estradiol (3, **⇑⇑**); production of 4-androstene-3,17-dione (4, **⇓**) | 16 **⇑** |
| Amino acid metab | Hydroxylation of amino acid (3, **⇓⇓**); metabolism of S-adenosylmethionine (3, **⇓⇓**); degradation of L-dopa (2, **⇓**); depletion of S-adenosylmethionine (2, **⇓**) | 9 **⇓** |
| Drug metabolism | Binding of β-estradiol (3, **⇑⇑**); degradation of L-dopa (2, **⇓**); clearance of drug (2, ⇔) | 7 **↑** |
| Organismal devel | Arrest development of organ (6, **⇑**); development process of virus (3, ⇔); fibrillogenesis (3, **↑**) | 16 **↓** |
| Post-trans mod | O-glycosylation (5, **⇑**); isomerization of protein (2, **⇓**); prenylation of protein (2, **⇓**) | 14 ⇔ |
| Conn tissue D & F | Proliferation of fibroblasts (36, **↓**); adhesion of fibroblasts (9, ⇔); entry into S-phase of fibroblasts (6, ⇔); proliferation of pericytes (6, ⇔); quantity of fibroblasts (5, ⇔); growth of stromal cells (3, **↑**); proliferation of dermal fibroblasts (3, ⇔) | 57 **↓** |
| Skel muscle D & F | Fusion of myoblasts (7, ⇔); proliferation of myoblasts (5, **↓**) | 14 ⇔ |
| Free radical scav | Production of reactive oxygen species (23, **⇑**); accumulation of reactive oxygen species (6, **⇑⇑**); release of superoxide (6, **⇓**) | 32 **⇑** |
| Protein synthesis | Refolding of protein (2, ⇔); biosynthesis of cytokines (3, **⇓⇓**) | 5 **⇓** |

**TABLE R18**. Tabulated results from Ingenuity Pathway Analysis® (IPA) at **300 vs. 240d**. Reported are the functions with an Exact Fisher test P-value ≤ 0.05 sorted by decrease in significance. The category denotes the main functional category assigned by IPA. The functional annotation is derived by the “effect on function” in IPA. In parenthesis are reported the number of DEG for each specific function and the arrows denote the overall effect on the function inferred by the gene annotation using IPA (⇑⇑ = highly activated; ⇑ = activated; 🡩 = tend to be activated; ⇓⇓ = highly inhibited; ⇓ = inhibited; 🡫 = tend to be inhibited). For detailed explanation see Additional file 2. Some annotations on functions are common between categories and were not repeated and the overall effect takes into consideration common functions among categories. Some categories do not make biological sense based on mammary gland features (see Additional file 2 for details explanation). For those two reasons the sum of the numbers in parenthesis very often does not correspond with the total DEG in the left column (DEG)

| **Category** | **Function annotation** | **DEG** |
| --- | --- | --- |
| Molecular Transport | Quantity of hormone (6, **⇑**), beta-estradiol (4, ⇔), progesterone (4, **↑**), testosterone (4, **⇑**)**; **transport of mitochondrial membrane (3, **⇓⇓), **Cu2+ (3, **↑**); deposition of proteoglycan (2, **↑**); localization of lipid (2, **⇑**); binding of progesteron (2, **⇓**) | 22 **↑** |
| Carbohydrate met | Deposition of proteoglycan (2, **↑**); metabolic process of glycerol (2, ⇔); secretion of glycosaminoglycan [hyaluronic acid] (2, ⇔); localization of phosphatidylserine (1, **↑ [***CASP3↑*]); metabolism of xilulose (1,** ↑ [***DCXR↑*]); quantity of chondroitin sulfate proteoglycan (1, **↓ [***PSEN1↓*]) | 9 **↑** |
| Endocrine Syst Devel & Function | Binding of hormone (3, **⇓⇓**), estrogen (2, **⇓**), progesterone (2, **⇓**) | 9 **⇓** |
| Lipid metabolism | Quantity of β-estradiol (4, ⇔), progesterone (4, **↑**), testosterone (4, **⇑**); binding of estrogen (2, **⇓**), progesterone (2, **⇓**); localization of lipid (2, **⇑**); localization of phosphatidylserine (1, **↑ [***CASP3↑*]); reduction of prostaglandin F2alpha (1, **↑ [***AKR1A1↑*]); secretion of 4-androstene-3,17-dione (1, **↓ [***ESR1↓*]) | 12 **↑** |
| Organ Development | Organogenesis (20, ⇔) | 22 ⇔ |
| Molecule Biochem | Metabolism of polyamines (2, **⇓**) | 27 **⇑** |
| Cellular Development | Differentiation (19, **⇓**), neural stem cells (3, **↓**); developmental process of effector T lymphocytes (3, **↑**), muscle cell lines (7, **⇓**); commitment of cells (7, **⇑**); morphogenesis of epithelial cells (2, **↑**); myogenesis of muscle cell lines (3, **↓**) | 30 **⇓** |
| Cell morphology | Morphology of cells (21, **↑**); transformation of cells (21, **↑**) | 39 **↑** |
| Cell growth & prol | Colony formation (16,** ↓**); quantity of cells (10,** ↑** ); proliferation of effector T lymphocytes (3, **⇓**) | 28 **↓** |
| Cell-cell sign & inter | Response of cells (12, **⇑**); cell-cell contact (6, **⇓**); adhesion of fibroblasts (4, **⇓⇓**); potentiation of synapse (3, **⇑**) | 26 **↑** |
| Cell death | Apoptosis (36, **↑**), monocytes (3, **⇑**) | 41 **↑** |
| Gene expression | Activation of cAMP response element (6, **⇑⇑**); expression of antioxidant response element (2, **⇓**), p53 response element (2, **↓**); stimulation of cAMP response element (2, **⇑**); transcription of GATA site (2, **⇑**) | 16 **⇑** |
| Tissue development | Developmental process of tissue (27, ⇔); muscle (10, **⇓⇓**) | 30 **↓** |
| Hem sys D & F | Movement of leukocytes (5, **↓**), B lymphocytes (3, **⇑**); homing of leukocytes (4, **↓**); infiltration of eosinophils (3, **⇑**), B lymphocytes (2, **⇑**); proliferation of T lymphocytes (3, **↓**) | 14 ⇔ |
| Immune response | Cell movement of leukocytes (5, **↓**), B lymphocytes (3, **⇑⇑**); homing of leukocytes cells (4, **↓**); infiltration of eosinophils (3, **⇑**); B lymphocytes (2, **⇑**); proliferation of effector T lymphocytes (3, **⇑**); cytotoxic T lymphocytes response of mice (2, ⇔); inflammation of eukaryotic cells (2, **⇑**); Th2 immune response (2, ⇔); *included also several annotated functions with 1 gene* | 13 ⇔ |
| Im & lym S D & F | Quantity of dendritic cells (4, **↑**) | 8 **↑** |
| Nervous sys D & F | Long-term potentiation (7, **↑**); differentiation of neuronal progenitor cells (3, **↓**); quantity of bipolar cells (2, **⇓**) | 17 **↑** |
| Drug metabolism | Quantity of β-estradiol (4, ⇔), progesterone (4, **↑**), testosterone (4, **⇑**); binding of progesterone (2, **⇓**); secretion of glycosaminoglycan [hyaluronic acid] (2, ⇔); clearance of lithium (1, **↑ [***PVALB↑*]); reduction of folic acid (1, **↓ [***DHFR↓*]); transport of thyroxine (1, **↑ [***SLCO1A2↑*]) | 14 **↑** |
| Cell cycle | Cell cycle progression of leukocytes (3, **↓**); G1/S phase transition of connective tissue cells (2, **⇓**) | 13 **↓** |
| Tissue morphology | Quantity of dendritic cells (4, **↑**), bipolar cells (2, **⇓**), neuronal progenitor cells (2, **⇑**), adhesions (1, **↓ [***CCR5↓*]); dysplasia of epithelial tissue (2, **⇓**) | 16 ⇔ |
| Tissue morphology | Dysplasia of epithelial cells (2, **⇓**); quantity of bipolar cells (2, **⇓**), neuronal progenitor cells (2, **⇓**) | 16 **↓** |
| Cell movement | Movement (30,** ↑** ) | 31 **↑** |
| Cell compromise | Proteolysis of protein (4, **↑**); permeability transition (3, ⇔); breakage of chromosomes (2, **↓**); degeneration of striatal neurons (2, **⇓**) | 14 ⇔ |
| Conn tissue D & F | Adhesion of fibroblasts (4, **⇓⇓**); G1/S phase transition of fibroblasts (2, **⇓**) | 9 **⇓** |
| Protein degradation | Hydrolisis and proteolysis of proteins (4, ⇔) | 4 ⇔ |
| DNA rep rec rep | Breakage of chromosomes (2, **↓**); delay in fragmentation of DNA (2, ⇔); quantity of chromosomes (2, ⇔); unwinding of origin of replication in short region (1, **↓ [***DNAJB1↓*]) | 6 **↓** |
| Sk muscle D & F | Development of muscle (10, **⇓⇓**); myogenesis (3, **⇓**) | 18 **⇓** |
| Cell Assem & Organ | Formation of lamellipodia (6, ⇔); quantity of chromosomes (2, ⇔); *included also several annotated functions with 1 gene (mostly* ⇓*)* | 16 ⇔ |
| Cell funct & maint | Endocytosis (7, **⇓⇓**); *included also several annotated functions with 1 gene (all* ⇓*)* | 13 **⇓** |
| Post-transl modif | Protein cleavage (2, **⇑**); aggregation of F-actin (1, **↓ [***SHROOM3↓*]) | 3 **↑** |
| Amino acid metab | Reduction of folic acid (1, **↓ [***DHFR↓*]); transport of thyroxine (1, **↑ [***SLCO1A2↑*]) | 2 ⇔ |
| Cell signaling | Excretion of calcium (1, **↑ [***PVALB↑*]); exhalation of nitric oxide (1, **↑ [***IL10↑*]); Tie receptor signaling pathway (1,** ↓ [***TEK↓*]) | 3 **↑** |
| Hair & Skin D & F | Function of epithelial barrier (1,** ↑ [***IL10↑*]); stratification of keratanocytes (1,** ↓ [***CDH1↓*]); structural integrity of epithelial barrier (1,** ↑ [***IL10↑*]); | 2 ⇔ |
| Organismal develop | Development of mammalia (8, **↓**) | 10 **↓** |
| Protein synthesis | *Included only 1 gene (FARS1,* **⇑**) | 1 **↑** |
| Protein trafficking | Localization of F-actin (1, **↓ [***SHROOM3↓*]) | 1 **↓** |
| RNA postrans mod | Unwinding of siRNA (1, **↓ [***DDX58↓*]) | 1 **↓** |
| Vit & Mineral Met | Excretion of calcium (1, **↑ [***PVALB↑*]); reduction of folic acid (1, **↓ [***DHFR↓*]) | 2 ⇔ |
| Organismal function | Recovery of mice (2, **⇓**) | 2 **⇓** |

Reproductive system development and function, Cardiovascular system development and function, and Organ morphology were not considered because they were not pertinent to mammary gland

# REFERENCES

1. Lemay DG, Neville MC, Rudolph MC, Pollard KS, German JB: **Gene regulatory networks in lactation: identification of global principles using bioinformatics**. *BMC Syst Biol* 2007, **1**:56.

2. Huang da W, Sherman BT, Lempicki RA: **Bioinformatics enrichment tools: paths toward the comprehensive functional analysis of large gene lists**. *Nucleic Acids Res* 2009, **37**(1):1-13.

3. Draghici S, Khatri P, Tarca AL, Amin K, Done A, Voichita C, Georgescu C, Romero R: **A systems biology approach for pathway level analysis**. *Genome Res* 2007, **17**(10):1537-1545.

4. Piantoni P, Bionaz M, Graugnard DE, Daniels KM, Everts RE, Rodriguez-Zas SL, Lewin HA, Hurley HL, Akers M, Loor JJ: **Functional and gene network analyses of transcriptional signatures characterizing pre-weaned bovine mammary parenchyma or fat pad uncovered novel inter-tissue signaling networks during development**. *BMC Genomics* 2010, **11**:331.

5. Rudolph MC, McManaman JL, Phang T, Russell T, Kominsky DJ, Serkova NJ, Stein T, Anderson SM, Neville MC: **Metabolic regulation in the lactating mammary gland: a lipid synthesizing machine**. *Physiological Genomics* 2007, **28**(3):323-336.

6. Bionaz M, Loor JJ: **Gene networks driving bovine milk fat synthesis during the lactation cycle**. *BMC Genomics* 2008, **9**(1):366.

7. Forsyth IA: **The Endocrinology of Lactation**. In: *Biochemistry of Lactation.* Edited by Mepham TB. Amsterdam: Elsevier Science Publishers B.V.; 1983: 309-349.

8. Keenan TW, James Morré D, Huang CM: **Membranes and the Mammary Gland**. In: *Lactation: a comprehensive treatise.* Edited by Larson BL, Smith VR, vol. 2. New York: Academic Press; 1974: 191-233.

9. Bauman DE, Davis CL: **Biosynthesis of milk fat**. In: *Lactation: a comprehensive treatise.* Edited by Larson BL, Smith VR, vol. 2. New York: Academic Press; 1974: 31-75.

10. Loor JJ, Dann HM, Everts RE, Rodriguez-Zas SL, Lewin HA, Drackley JK: **Mammary and hepatic gene expression analysis in peripartal dairy cows using a bovine cDNA microarray**. *J Dairy Sci* 2004, **87**(Suppl. 1):T134.

11. Finucane KA, McFadden TB, Bond JP, Kennelly JJ, Zhao FQ: **Onset of lactation in the bovine mammary gland: gene expression profiling indicates a strong inhibition of gene expression in cell proliferation**. *Funct Integr Genomics* 2008, **8**(3):251-264.

12. Joris I, Majno G, Corey EJ, Lewis RA: **The mechanism of vascular leakage induced by leukotriene E4. Endothelial contraction**. *Am J Pathol* 1987, **126**(1):19-24.

13. Mehta D, Malik AB: **Signaling mechanisms regulating endothelial permeability**. *Physiol Rev* 2006, **86**(1):279-367.

14. DePeters EJ, Cant JP: **Nutritional factors influencing the nitrogen composition of bovine milk: a review**. *J Dairy Sci* 1992, **75**(8):2043-2070.

15. Baumrucker CR: **Amino acid transport systems in bovine mammary tissue**. *J Dairy Sci* 1985, **68**(9):2436-2451.

16. Lemay DG, Lynn DJ, Martin WF, Neville MC, Casey TM, Rincon G, Kriventseva EV, Barris WC, Hinrichs AS, Molenaar AJ *et al*: **The bovine lactation genome: insights into the evolution of mammalian milk**. *Genome Biol* 2009, **10**(4):R43.

17. Bionaz M, Loor JJ: **mTOR, AMPK, and insulin receptor signaling networks in the bovine mammary gland during the lactation cycle**. *Faseb Journal* 2007, **21**(6):A1109-A1109.

18. Menzies KK, Lefevre C, Macmillan KL, Nicholas KR: **Insulin regulates milk protein synthesis at multiple levels in the bovine mammary gland**. *Funct Integr Genomics* 2009, **9**(2):197-217.

19. Delehedde M, Lyon M, Sergeant N, Rahmoune H, Fernig DG: **Proteoglycans: pericellular and cell surface multireceptors that integrate external stimuli in the mammary gland**. *J Mammary Gland Biol Neoplasia* 2001, **6**(3):253-273.

20. Tao N, DePeters EJ, Freeman S, German JB, Grimm R, Lebrilla CB: **Bovine milk glycome**. *J Dairy Sci* 2008, **91**(10):3768-3778.

21. Sonnino S, Mauri L, Chigorno V, Prinetti A: **Gangliosides as components of lipid membrane domains**. *Glycobiology* 2007, **17**(1):1R-13R.

22. Newburg DS, Chaturvedi P: **Neutral glycolipids of human and bovine milk**. *Lipids* 1992, **27**(11):923-927.

23. Tao N, DePeters EJ, German JB, Grimm R, Lebrilla CB: **Variations in bovine milk oligosaccharides during early and middle lactation stages analyzed by high-performance liquid chromatography-chip/mass spectrometry**. *J Dairy Sci* 2009, **92**(7):2991-3001.

24. Lee WJ, Monteith GR, Roberts-Thomson SJ: **Calcium transport and signaling in the mammary gland: targets for breast cancer**. *Biochim Biophys Acta* 2006, **1765**(2):235-255.

25. Neville MC: **Calcium secretion into milk**. *J Mammary Gland Biol Neoplasia* 2005, **10**(2):119-128.

26. Neville MC, Keller RP, Casey C, Allen JC: **Calcium partitioning in human and bovine milk**. *J Dairy Sci* 1994, **77**(7):1964-1975.

27. Shennan DB, Peaker M: **Transport of milk constituents by the mammary gland**. *Physiol Rev* 2000, **80**(3):925-951.

28. Kroger M: **General Environmental Contaminants Occuring in Milk**. In: *Lactation: a comprehensive treatise.* Edited by Larson BL, Smith VR, vol. 3. New York: Academic Press; 1974: 135-157.

29. Ito S, Alcorn J: **Xenobiotic transporter expression and function in the human mammary gland**. *Adv Drug Deliv Rev* 2003, **55**(5):653-665.

30. Tucker HA: **Hormones, mammary growth, and lactation: a 41-year perspective**. *J Dairy Sci* 2000, **83**(4):874-884.

31. Bionaz M, Loor JJ: **Identification of reference genes for quantitative real-time PCR in the bovine mammary gland during the lactation cycle**. *Physiol Genomics* 2007, **29**(3):312-319.

32. Delbecchi L, Miller N, Prud'homme C, Petitclerc D, Wagner GF, Lacasse P: **17 beta-estradiol reduces milk synthesis and increases stanniocalcin gene expression in the mammary gland of lactating cows**. *Livestock Production Science* 2005, **98**(1-2):57-66.

33. Akama KT, McEwen BS: **Estrogen stimulates postsynaptic density-95 rapid protein synthesis via the Akt/protein kinase B pathway**. *J Neurosci* 2003, **23**(6):2333-2339.

34. Feng Y, Manka D, Wagner KU, Khan SA: **Estrogen receptor-alpha expression in the mammary epithelium is required for ductal and alveolar morphogenesis in mice**. *Proc Natl Acad Sci U S A* 2007, **104**(37):14718-14723.

35. Li RW, Capuco AV: **Canonical pathways and networks regulated by estrogen in the bovine mammary gland**. *Funct Integr Genomics* 2008, **8**(1):55-68.

36. Diamond JM: **Mammary gland as an endocrine organ: implications for mastectomy**. *Nature* 1982, **295**(5846):191-192.

37. Bionaz M, Loor JJ: **ACSL1, AGPAT6, FABP3, LPIN1, and SLC27A6 are the most abundant isoforms in bovine mammary tissue and their expression is affected by stage of lactation**. *J Nutr* 2008, **138**(6):1019-1024.

38. Capuco AV, Wood DL, Baldwin R, McLeod K, Paape MJ: **Mammary cell number, proliferation, and apoptosis during a bovine lactation: relation to milk production and effect of bST**. *J Dairy Sci* 2001, **84**(10):2177-2187.

39. Norgaard JV, Theil PK, Sorensen MT, Sejrsen K: **Cellular mechanisms in regulating mammary cell turnover during lactation and dry period in dairy cows**. *J Dairy Sci* 2008, **91**(6):2319-2327.

40. Lavrik I, Golks A, Krammer PH: **Death receptor signaling**. *J Cell Sci* 2005, **118**(Pt 2):265-267.

41. Bionaz M, Loor JJ: **Comparative MammOmics™ of milk fat synthesis in Mus musculus vs. Bos taurus**. *J Dairy Sci* 2008, **91**(Suppl. 1):566-567.

42. Ehrenhofer-Murray AE: **Chromatin dynamics at DNA replication, transcription and repair**. *Eur J Biochem* 2004, **271**(12):2335-2349.

43. Serfling E, Berberich-Siebelt F, Avots A, Chuvpilo S, Klein-Hessling S, Jha MK, Kondo E, Pagel P, Schulze-Luehrmann J, Palmetshofer A: **NFAT and NF-kappaB factors-the distant relatives**. *Int J Biochem Cell Biol* 2004, **36**(7):1166-1170.

44. Polager S, Ginsberg D: **E2F - at the crossroads of life and death**. *Trends Cell Biol* 2008, **18**(11):528-535.

45. Jerry DJ, Dickinson ES, Roberts AL, Said TK: **Regulation of apoptosis during mammary involution by the p53 tumor suppressor gene**. *J Dairy Sci* 2002, **85**(5):1103-1110.

46. Nerlov C: **The C/EBP family of transcription factors: a paradigm for interaction between gene expression and proliferation control**. *Trends Cell Biol* 2007, **17**(7):318-324.

47. Smallwood A, Esteve PO, Pradhan S, Carey M: **Functional cooperation between HP1 and DNMT1 mediates gene silencing**. *Genes Dev* 2007, **21**(10):1169-1178.

48. Watson CJ, Neoh K: **The Stat family of transcription factors have diverse roles in mammary gland development**. *Semin Cell Dev Biol* 2008, **19**(4):401-406.

49. Bionaz M, Loor JJ: **Gene networks driving bovine mammary protein synthesis during the lactation cycle**. *Bioinform Biol Insights* 2011, **5**:83-98.

50. Liu F: **Receptor-regulated Smads in TGF-beta signaling**. *Front Biosci* 2003, **8**:s1280-1303.

51. Choi KM, Barash I, Rhoads RE: **Insulin and prolactin synergistically stimulate beta-casein messenger ribonucleic acid translation by cytoplasmic polyadenylation**. *Mol Endocrinol* 2004, **18**(7):1670-1686.

52. Nusrat A, Giry M, Turner JR, Colgan SP, Parkos CA, Carnes D, Lemichez E, Boquet P, Madara JL: **Rho protein regulates tight junctions and perijunctional actin organization in polarized epithelia**. *Proc Natl Acad Sci U S A* 1995, **92**(23):10629-10633.

53. Itoh M, Bissell MJ: **The organization of tight junctions in epithelia: implications for mammary gland biology and breast tumorigenesis**. *J Mammary Gland Biol Neoplasia* 2003, **8**(4):449-462.

54. Kolch W: **Coordinating ERK/MAPK signalling through scaffolds and inhibitors**. *Nat Rev Mol Cell Biol* 2005, **6**(11):827-837.

55. Keren A, Tamir Y, Bengal E: **The p38 MAPK signaling pathway: a major regulator of skeletal muscle development**. *Mol Cell Endocrinol* 2006, **252**(1-2):224-230.

56. Akers RM: **Major advances associated with hormone and growth factor regulation of mammary growth and lactation in dairy cows**. *J Dairy Sci* 2006, **89**(4):1222-1234.

57. Anderson RR: **Endocrinological Control**. In: *Lactation: a comprehensive treatise.* Edited by Larson BL, Smith VR, vol. 1. New York: Academic Press; 1974: 97-140.

58. Pitelka D, Hamamoto ST: **Ultrastructure of the Mammary Secretory Cell**. In: *Biochemistry of Lactation.* Edited by Mepham TB. Amsterdam: Elsevier Science Publishers B.V.; 1983: 29-70.

59. Bar D, Tauer LW, Bennett G, Gonzalez RN, Hertl JA, Schukken YH, Schulte HF, Welcome FL, Grohn YT: **The cost of generic clinical mastitis in dairy cows as estimated by using dynamic programming**. *J Dairy Sci* 2008, **91**(6):2205-2214.

60. Vorbach C, Capecchi MR, Penninger JM: **Evolution of the mammary gland from the innate immune system?** *Bioessays* 2006, **28**(6):606-616.

61. Sladek Z, Ryznarova H, Rysanek D: **Macrophages of the bovine heifer mammary gland: morphological features during initiation and resolution of the inflammatory response**. *Anat Histol Embryol* 2006, **35**(2):116-124.

62. Seelig LL, Jr.: **Dynamics of leukocytes in rat mammary epithelium during pregnancy and lactation**. *Biol Reprod* 1980, **22**(5):1211-1217.

63. Hollmann KH: **Cytology and Fine Structure of the Mammary Gland**. In: *Lactation: a comprehensive treatise.* Edited by Larson BL, Smith VR, vol. 1. New York: Academic Press; 1974: 3-95.

64. Fitzpatrick JL, Wilson AD, Bland PW, Stokes CR: **Expression of major histocompatibility complex (MHC) class II antigens in the murine mammary gland**. *Immunol Lett* 1993, **35**(1):7-11.

65. Fitzpatrick JL, Mayer SJ, Vilela C, Bland PW, Stokes CR: **Cytokine-induced major histocompatibility complex class II antigens on cultured bovine mammary gland epithelial cells**. *J Dairy Sci* 1994, **77**(10):2940-2948.

66. Paulsson KM: **Evolutionary and functional perspectives of the major histocompatibility complex class I antigen-processing machinery**. *Cell Mol Life Sci* 2004, **61**(19-20):2446-2460.

67. Rupp R, Boichard D: **Genetics of resistance to mastitis in dairy cattle**. *Vet Res* 2003, **34**(5):671-688.

68. Moyes KM, Drackley JK, Morin DE, Bionaz M, Rodriguez-Zas SL, Everts RE, Lewin HA, Loor JJ: **Gene network and pathway analysis of bovine mammary tissue challenged with Streptococcus uberis reveals induction of cell proliferation and inhibition of PPARgamma signaling as potential mechanism for the negative relationships between immune response and lipid metabolism**. *BMC Genomics* 2009, **10**:542.

69. Rauw WM, Kanis E, Noordhuizen-Stassen EN, Grommers FJ: **Undesirable side effects of selection for high production efficiency in farm animals: a review**. *Livestock Production Science* 1998, **56**(1):15-33.

70. Sibaja RA, Schmidt GH: **Epinephrine inhibiting milk ejection in lactating cows**. *J Dairy Sci* 1975, **58**(3):344-348.

71. Segal SS: **Regulation of blood flow in the microcirculation**. *Microcirculation* 2005, **12**(1):33-45.

72. Linzell JL: **Mammary Blood Flow and Substrate Uptake**. In: *Lactation: a comprehensive treatise.* Edited by Larson BL, Smith VR, vol. 1. New York: Academic Press; 1974: 143-225.

73. Simon MC, Liu L, Barnhart BC, Young RM: **Hypoxia-induced signaling in the cardiovascular system**. *Annu Rev Physiol* 2008, **70**:51-71.

74. Brouty-Boye D: **Developmental biology of fibroblasts and neoplastic disease**. *Prog Mol Subcell Biol* 2005, **40**:55-77.

75. Hovey RC, Trott JF, Vonderhaar BK: **Establishing a framework for the functional mammary gland: from endocrinology to morphology**. *J Mammary Gland Biol Neoplasia* 2002, **7**(1):17-38.

76. Grosvenor CE, Mena F: **Neural and Hormonal Control of Milk Secretion and Milk Ejection**. In: *Lactation: a comprehensive treatise.* Edited by Larson BL, Smith VR, vol. 1. New York: Academic Press; 1974: 227-276.

77. Lamote I, Meyer E, Massart-Leen AM, Burvenich C: **Sex steroids and growth factors in the regulation of mammary gland proliferation, differentiation, and involution**. *Steroids* 2004, **69**(3):145-159.

78. Sarkadi B, Homolya L, Szakacs G, Varadi A: **Human multidrug resistance ABCB and ABCG transporters: participation in a chemoimmunity defense system**. *Physiol Rev* 2006, **86**(4):1179-1236.

79. Pape-Zambito DA, Magliaro AL, Kensinger RS: **17Beta-estradiol and estrone concentrations in plasma and milk during bovine pregnancy**. *J Dairy Sci* 2008, **91**(1):127-135.

80. Knight CH, Peaker M, Wilde CJ: **Local control of mammary development and function**. *Rev Reprod* 1998, **3**(2):104-112.

81. Svennersten-Sjaunja K, Olsson K: **Endocrinology of milk production**. *Domest Anim Endocrinol* 2005, **29**(2):241-258.

82. Aitken SL, Karcher EL, Rezamand P, Gandy JC, VandeHaar MJ, Capuco AV, Sordillo LM: **Evaluation of antioxidant and proinflammatory gene expression in bovine mammary tissue during the periparturient period**. *J Dairy Sci* 2009, **92**(2):589-598.
