# Supplementary material for: A Novel Dynamic Impact Approach (DIA) for Functional Analysis of Time-Course Omics Studies: Validation Using the Bovine Mammary Transcriptome
Source: PLoS One. 2012 Mar 16;7(3):e32455. doi: 10.1371/journal.pone.0032455 (PMC3306320; doi:10.1371/journal.pone.0032455)
Supplement: File S2 — Additional Materials and Methods. (DOC) [file pone.0032455.s008.doc]

ADDITIONAL MATERIALS AND METHODS

Contents

[Enrichment analysis 2](#__RefHeading___Toc310860200)

[ORA by Ingenuity Pathway Analysis (IPA) 2](#__RefHeading___Toc310860201)

[Methodological note 2](#__RefHeading___Toc310860202)

[Data mining 2](#__RefHeading___Toc310860203)

[Approach used in IPA 3](#__RefHeading___Toc310860204)

[Criteria used to interpret the IPA functional analysis 4](#__RefHeading___Toc310860205)

[Enrichment analysis by DAVID 5](#__RefHeading___Toc310860206)

[Technical limitations in data mining. 6](#__RefHeading___Toc310860207)

[Dynamic impact approach (DIA) 6](#__RefHeading___Toc310860208)

[Rationale 6](#__RefHeading___Toc310860209)

[Assumptions 8](#__RefHeading___Toc310860210)

[Advantages of DIA 9](#__RefHeading___Toc310860211)

[Calculations and detailed protocol 10](#__RefHeading___Toc310860212)

[AUTOMATIC ANALYSIS 10](#__RefHeading___Toc310860213)

[MANUAL ANALYSIS 13](#__RefHeading___Toc310860214)

[Limitations and future implementation of DIA 17](#__RefHeading___Toc310860215)

# Enrichment analysis

## ORA by Ingenuity Pathway Analysis (IPA)

## Methodological note

The data interpretation takes into account the possible influence of several cell types within the core mammary biopsy tissue. Mammary tissue of rodents has a large degree of variation in the ratio of epithelial/adipose cells which is an important confounding factor during interpretation of mammary transcriptomic data . In contrast, bovine mammary gland from multiparous cows is composed of >70% epithelial cells (excluding extra-parenchymal tissue) with very little variation between stages of lactation, except for a significant increase at peak lactation (ca. 80% at 90 days of lactation) . Remaining cells are mainly fibroblasts (or connective tissue; ca. 14%), endothelium (ca. 7%), and leukocytes (ca. 5% in the rat with a possible increase between non-lactating and lactating periods . Other cells composing the mammary gland include neurons, which seem to travel with capillaries , and “basket” which is composed of smooth muscle cells (estimated to be ca. 5% of the entire gland ). Unlike rodents, the presence of adipose cells in lactating mammary tissue of multiparous cows is negligible. In addition, the biopsy procedure used in the present experiment to harvest mammary tissue likely augments the percentage of epithelial cells over connective tissue. Therefore, epithelial cells were expected to predominate in the samples analyzed.

## Data mining

Networks, functions, and pathways analyses were generated using IPA (Ingenuity Systems, [http://www.ingenuity.com](http://www.ingenuity.com/), Redwood City, CA) which assists with microarray data interpretation via grouping DEG into known functions, pathways, and networks based primarily on human and rodent studies. In addition, data were analyzed using DAVID .

### Approach used in IPA

The entire microarray data with their associated annotation (when present) with LSmean (after back-transformation), overall FDR, and the post-hoc P-value of each comparison were uploaded into IPA. The entire microarray was used as background or reference dataset for enrichment calculation of enriched functions/pathways. Each annotated gene was mapped to its corresponding gene object in the IPA Knowledge Base. The analysis was run using the following setting in IPA: all defaults setting for the selection of dataset, no fold-change cut-off, FDR = 0.001 and P-value = 0.001. Several analyses were run in IPA:

- ***Functional Analysis***. The functional analysis in IPA identified the biological functions that were most significant to the data set. To minimize false positives among significantly-enriched functions an FDR ≤0.05 (-log *P*-value = 1.33) was used to determine the probability that each biological function assigned to that data set was due to chance alone. Also the simple P-value for the enrichment was considered and reported. All functions were used except the one related to diseases.

- ***Canonical Pathway Analysis***: canonical pathway analysis identified the pathways from the IPA library that were most significant to the data set. Genes from the data set that were associated with a canonical pathway in the IPA Knowledge Base were considered for the analysis. The significance of the association between the data set and the canonical pathway was measured in 2 ways: 1) a ratio of the DEG that mapped to the pathway divided by the total number of genes that mapped to the canonical pathway; 2) an FDR ≤ 0.05 to calculate a *P*-value determining the probability that the association between the DEG and the signaling canonical pathway was explained by chance alone. A simple P-value was also considered and reported in the results.

Analysis in IPA were run both using the Benjamini-Hochberg FDR correction and simple P-value (i.e., not FDR corrected). Results were downloaded as txt files and data shown graphically using SigmaPlot 10 and details of the functions reported as Tables.

### Criteria used to interpret the IPA functional analysis

The description of the functions in IPA was a consideration of the response of the genes (up- or down-regulated) and the "effect on function" feature in IPA as reported in Piantoni et al. . The final evaluation on the effect on any particular function was an extrapolation of the ensemble following these criteria:

- when a function in IPA "effect of function" had a number of genes in "increase/decrease function" that was <10% higher from those in "decrease/increase function" including genes in "affect function" which evidently induce or inhibit the function (assessed by carefully considering the IPA links which include IPA descriptions or the original papers for those functions) the functions were considered to be in equilibrium or not having a net effect (or not evident net effect). Further, even though the function was significantly enriched with DEG, a final judgment of a biological outcome was not feasible, thus the function was considered in equilibrium (denoted by ↔).
- when a function in IPA "effect of function" had a number of genes in "increase/decrease function" that was ≥ 10% higher from those in "decrease/increase function" including genes in "affect function", which evidently induce or inhibit the function (as reported above), the function "tends to increase/decrease (or induced/inhibited)" which for simplicity was denoted with arrows (tendency to induce or increase = ↑; tendency to inhibit or decrease = ↓);
- when the number of genes which increase/induce or decrease/inhibit the function was ≥ 100% more (or ≥2-fold) compared to decrease/inhibit or increase/induce, the function was considered to be evidently induced or inhibited (simple arrows ⇑ or ⇓);
- when all, or nearly all, the genes found in increase/induce or decrease/inhibit function or the analysis of "affect function" stated that they were involved in inducing or inhibiting the function, the function was considered to be completely induced or inhibited (⇑⇑ or ⇓⇓);
- genes which were up-regulated and were found in "decrease function" were considered to actively decrease or inhibit the function;
- genes which were down-regulated and were found in "decrease function" were considered to decrease the function and also to allow the function to take place;- genes which were up-regulated and were associated with "increase function" were considered to increase or induce the function;
- genes which were down-regulated and were associated with "increase function" were considered as failing to increase or induce the function;- the final evaluation on the state of a particular function was a sum of all up- and down-regulated genes.

## Enrichment analysis by DAVID

The lists of up-regulated and down-regulated DEG with relative Entrez Gene ID for each comparison (i.e., FDR ≤ 0.001 and P-value between comparison ≤ 0.001) were uploaded in DAVID (<http://david.abcc.ncifcrf.gov/>) and the whole annotated microarray was used as background for the enrichment analysis. The results obtained by the default conditions in DAVID (i.e., minimum 2 genes per term, EASE score ≤ 0.10) were downloaded. The annotations for each general group of annotation down-loaded were:

COG_ONTOLOGY, SP_PIR_KEYWORDS, and UP_SEQ_FEATURE among Functional_Categories;

- all GOTERM among Gene_Ontology (but presented are only GOTERM_BP_FAT, GOTERM_CC_FAT, and GOTERM_MF_FAT);
- CHROMOSOME among General Annotations;
- KEGG_PATHWAY among Pathways;
- INTERPRO, PIR_SUPERFAMILY, SMART, and SSF among Protein_Domains;
- UP_TISSUE among Tissue_Expression.

The P-value and the Benjamini-Hochberg FDR were used to determine significance of enrichment or overrepresentation of terms for each annotation (e.g., Gene Ontology Biological process).

# Technical limitations in data mining.

Several limitations exist for data mining mainly due to the lack of mammary specific annotations as recognized previously , as well as lack of a complete bovine annotation. Additional limitations were present within IPA, which is based on human and rodent literature. Besides bioinformatics-related limitations, our approach for interpretation of IPA and DAVID data can present limitations, because the thresholds used to judge the direction of a function in IPA (⇑ or ⇓, see above) and the cut-off selected for DAVID were selected by the authors on grounds that can be arguable. Completion of the bovine annotation in concert with refinement of bioinformatics tools could in the future provide new insights using the same data generated in the present experiment.

# Dynamic impact approach (DIA)

## Rationale

Although it is likely that certain randomness in transcriptome expression could exist, this has to be minor considering that cells finely regulate the transcription of genes in order to orchestrate all the functions and pathways to survive and accomplish the tasks required for the survival of the whole organism. More importantly, this potential randomness (plus the “noise” due to the techniques used, i.e., microarray) in general is accounted for by the statistical analysis of the data; thus, all the genes found to be significantly affected can be considered to have consequences on the biology. In summary, we believe that the randomness is corrected by the statistical analysis of the transcriptomics data and all significant data are non-random; thus, no additional statistical analysis is required to interpret the biology of the tissue at study when evaluating the dynamism of the transcriptome.

Three factors are considered in the calculation of the Impact and Direction of the Impact in the DIA. The factors are:

1. % DEG vs. total genes in the array involved in the pathway/function: The observation that a treatment or change in physiological state causes a significant change in expression of a large number of proteins involved in a specific pathway would prompt us to conclude that the conditions under study have a large impact on that pathway. However, the final flux of the pathway is not just determined by the number of proteins that change but also by the direction of the change (i.e., down-regulated or up-regulated). For instance, a pathway where all proteins are significantly affected by a specific treatment can be highly impacted, but the direction of the flux is determined (considering all the proteins having the same positive effect on the pathway) by the number of up-regulated vs. the number of down-regulated proteins. When the ratio of up-regulated/down-regulated = 1, the overall flux can be considered unchanged, despite the fact that the treatment has a large impact on the pathway.
2. Magnitude of change of DEG: the magnitude of change of protein content also determines the impact on metabolic or signaling flux. For instance, if two treatments affect expression of the same proteins with the same direction of change (e.g., up-regulation) but one treatment changes the amount of proteins on average by 2-fold compared with the other treatment we would expect to have ca. twice as much flux in the former treatment compared to the latter.
3. Average significance of DEG: When considering the fact that we routinely use few biological samples to infer the effect of treatments or physiological state on the population at large, we also need to account for the significance of the change in protein/gene expression. For instance, if on the previous example the number of proteins and the magnitude of change (and direction of change) of the proteins was equal between the two treatments but in the first treatment the average significance was 100 × lower (e.g., P-value = 0.01 vs. P-value = 0.0001), then we can expect that the second treatment has 100-fold more chance to affect the pathway; thus a more pronounce effect on the pathway in the universal population. In other words, the fold change calculated is more likely to be real in the second treatment compared to the first.

In view of all the above points we propose that the transcriptome allows us to infer that a specific condition impacts a pathway (or any biological term) by examining 1) the proportion of significantly affected genes which code for proteins involved in such pathway compared to all genes coding for proteins involved in such pathway potentially measurable (i.e., by the microarray), 2) the average magnitude of the effect on DEG, and 3) the average significance of the DEG. For microarray analysis we only measure gene expression and not protein expression, but we assume that the expression of genes is proportional to expression of proteins. This is a gross, but unavoidable, assumption because the correlation between mRNA and protein is highly-variable between types of mRNA and estimated to be, in general, less than 0.5 .

## Assumptions

The DIA is based on the following assumptions which are common with the approaches using enrichment analysis such as Ingenuity Pathway Analysis and DAVID:

- All the expressed mRNA are translated into protein (i.e., does not and cannot account for alternative splice variants, inconsistency in mRNA/protein ratio), and no additional “control points”, besides the modulation in expression of genes, are considered. Thus, the method assumes an equivalence between quantity of change in genes and quantity of change in the encoded protein (i.e., differentially expressed genes [DEG] = differentially expressed protein => biological effect);
- All the DEG (=protein) have the same weight on the pathway and/or function (an approach for addressing this limitation has been proposed previously );

Assumptions that are unique to DIA are:

- If a gene is deemed to be significantly affected by the treatments or change in physiological state through a stringent statistical analysis the gene (=encoded protein) has to have an effect on the overall biology of the cell and no additional randomness can be considered (i.e., not additional statistical analysis need to be run); thus, all the DEG need to be accounted for to interpret the biological changes due to treatments and/or change in physiological state;
- The biological significance of the change on a pathway and/or function by a treatment and/or change in physiological state (=impact) is determined by:
  - ***Proportion of DEG compared to all the genes that can be measured*** (i.e., proportion of DEG with a role on the pathway and/or function relative to all the genes present on the microarray with a role on the pathway and/or function). The higher the proportion of DEG with a role on the pathway and/or function, the higher the overall impact of the treatment or change in physiological state on the pathway and/or function;
  - ***Amount of change of the DEG*** (i.e., fold change). The higher the mean of change in amount of mRNA of the DEG composing the pathway and/or function, the higher the biological impact.
  - ***Significance of change of the DEG***. The higher the mean of statistical significance of DEG composing the pathway and/or function in the samples, the higher the likelihood that the pathway and/or function is affected in the population at large.
- The overall direction of the impact (or flux when considering pathways) is determined by the total positive impact (i.e., activation; in general up-regulated genes) – total of negative impact (i.e., inhibition; in general down-regulated genes) on the function and/or pathway. For the KEGG pathway analysis, the genes that have a known negative impact on the pathway are considered to have an opposite effect (i.e., if the expression is increased the function and/or pathway is more inhibited).

## Advantages of DIA

The advantages of the DIA compared to enrichment analysis are:

- It allows visualizing the dynamism of each term (or cluster of terms) elicited by a treatment(s) and/or changes in physiological state. In turn, this allows to follow the behavior of each term through the experiment;
- It allows for an integrative view of all the terms simultaneously. This is particularly useful within the several annotation categories such as CHROMOSOME and KEGG pathway analysis, where relatively few terms (i.e., chromosomes and pathways) are present;
- For KEGG pathways analysis (but potentially implementable in other annotation categories as well) an overview of the main categories of pathways is provided, thus, allowing a rapid overall interpretation of the pathway analysis;
- It allows for an easy interpretation of the functional analysis because it provides an overall direction of the impact (or flux) on biological terms. This allows evaluating if the terms are induced or inhibited overall by the treatment(s) and/or change in physiological state.
- Because it allows comparing several different datasets from any kind of experiment (e.g., two separate tissues in the same organism, or the same tissue in different experimental conditions), it is a more suitable platform for implementing a true system biology approach, particularly for integrative system biology. The method, however cannot account for technical differences between techniques or tools (e.g., if microarray data are from different platforms differences in sensitivity of a platform can introduce biases in the comparison). An integrative system biology approach can be used if the same microarray platform is used, and the same statistical approach to determine DEG is undertaken.

## Calculations and detailed protocol

Two main ways for data analysis were implemented, one completely automatic and the other manual. The automatic calculation was implemented for use with small databases such as KEGG pathway, UP-TISSUE, and Chromosome; while the manual calculation was implemented for very large databases such as Gene Ontology and others.

### AUTOMATIC ANALYSIS

Free annotations for pathways in KEGG (Kyoto Encyclopedia of Genes and Genomes; at <ftp://ftp.genome.jp/pub/kegg/>, last up-date downloaded was April 2011, **now available only by subscription**, see note at <http://www.genome.jp/kegg/docs/relnote.html>) and UP-TISSUE and CHROMOSOME annotations available in DAVID (<http://david.abcc.ncifcrf.gov/>; gently sent upon request) with relative Entrez Gene ID (for *Homo sapiens*, *Mus musculus*, *Rat norvegicus*, and *Bos taurus*) were obtained. A general approach was undertaken with all the annotation databases, with an additional step when dealing with KEGG pathway.

The following system was implemented in Excel:

- The central core of the system is the calculation of the impact for % DEG compared to the array, mean log2 fold change, and mean –log10 P-value for DEG in each term. This provides the foundation for all the other calculations.
- The Excel file is composed of 5 (6 for KEGG pathway analysis) visible sheets:

1. A “General Information” sheet where general settings can be selected/applied, and provides basic information after running the calculations. The following information are required to be selected/applied:
   1. Species (selected by a dropdown menu of available species; at the present time the system is fully equipped to work with Bovine, Human, and Mouse, other species are listed but not yet implemented in the system);
   2. P-value cut-off between comparisons;
   3. The user has to indicate if the dataset has an FDR for the overall treatments/time effect or interactions (dropdown menu with YES or NO);
   4. If the FDR are available, then a cut-off needs to be applied;
   5. The user has to upload (by simple copy and paste) the complete Entrez Gene ID of the microarray used in ascending order (duplicates are allowed). The first Entrez Gene ID needs to be entered on the second row.
2. The complete microarray dataset (sheet named “DATA-DEG”) with the following information in columns from left to right:
3. Oligo ID of the microarray;
4. The Entrez Gene ID in ascending order;
5. Overall FDR for treatment(s) or time effect or interactions for each Entrez Gene ID;
6. For each comparison and for each Entrez Gene ID the first column (on the left) needs to have the ratio of gene expression (treatment/control or time point X/time point 0) and the second column (on the right) the p-value for the comparison. The system can run up to 20 comparisons simultaneously;
7. The results sheet (named “Flux and Impact”) with the reported name of the biological terms and associated overall impact (shown by a horizontal bar), and the direction of the impact or flux (shown in an adjacent cell [red = overall induced; green = overall inhibited; and yellow = no change in the flux) for each comparison. In addition, the most-impacted terms are highlighted in a specific column.
8. A macro that sorts the terms from the most impacted to the least both as overall mean impact;
9. A macro that sorts the terms from the most impacted to the least for each comparison;
10. For the KEGG pathway analysis an additional sheet is included where the calculation of the mean impact and flux for each category of pathways (as provided by KEGG at <http://www.genome.jp/kegg/pathway.html>) is performed. Both, for the main categories (Metabolism, Genetic Information Processing, Environmental Information Processing, Cellular Processes, Organismal Systems, and Human Diseases) and for the sub-categories of pathways. This allows for a quick overview of the overall effect of a treatment(s) and/or time points on a pathway.

- The Excel file contains 4 hidden calculation sheets. The calculations performed are:

1. The first step is to provide the correct list of terms for the selected species;
2. The second step is the calculation of the % genes on the microarray used that are associated with the annotated terms relative to the whole bovine genome. This increases the reliability of the data by selecting a minimum representation of terms on the microarray (see below).
3. The third step is the transformation to log2 fold change and –log10 P-value of data from all the comparisons of the genes present in all the terms (i.e., KEGG pathways) that pass the overall FDR threshold selected;
4. The fourth step is the calculation for each term and each comparison of the % DEG compared to the genes present on the array platform, the mean log2 fold change of the DEG, and the mean –log P-value of the DEG. This calculation is done separately by up- or down-regulated genes. This sheet also eliminates the terms which are deemed to be not adequately represented on the microarray compared to the whole genome (see second step above). This threshold can be selected in the “Flux and Impact” sheet. The minimum cut-off we suggest is 20% but we recommend using a higher cut-off such as 30% or 40%.

- Additional sheets with annotation datasets for each species are available to be used for calculations.

The results can be easily copied and used for further analysis.

### MANUAL ANALYSIS

The manual analysis has to be performed with very large annotation databases, such as Gene Ontology. The method makes use of DAVID (<http://david.abcc.ncifcrf.gov/>):

1. The lists of DEG with relative Entrez Gene ID separated for up- and down-regulated genes are uploaded using the “Multi-List File” option in DAVID. The entire annotated microarray (Entrez Gene ID) is uploaded as background (this is done in order to also download the enrichment analysis, but it is important in DIA for the calculation of % DEG vs. array platform, see below).
2. All the annotation terms of interest are selected and the “Functional Annotation Chart” function is launched. When the Functional Annotation Chart results are obtained the threshold options are changed (i.e., minimum of 1 gene and EASE score = 1) in order to obtain the entire association of Entrez Gene ID of DEG with terms. This is done for each comparison. The Functional Annotation Chart results are downloaded for all databases of interest together.
3. The Annotation Chart for all the comparisons with separated up- and down-regulated genes is assembled in a unique Excel sheet.
4. The column containing the genes within each annotation term for all the comparisons is copied and pasted it after the last column of data. Each gene symbol in this column is then separated by the “Text to columns” feature in Excel (in the “Data” tab)
5. Using the filter option in Excel each annotation database for all comparisons is selected and pasted in a news Excel file.

The Sheet 2 should have the columns as follows:


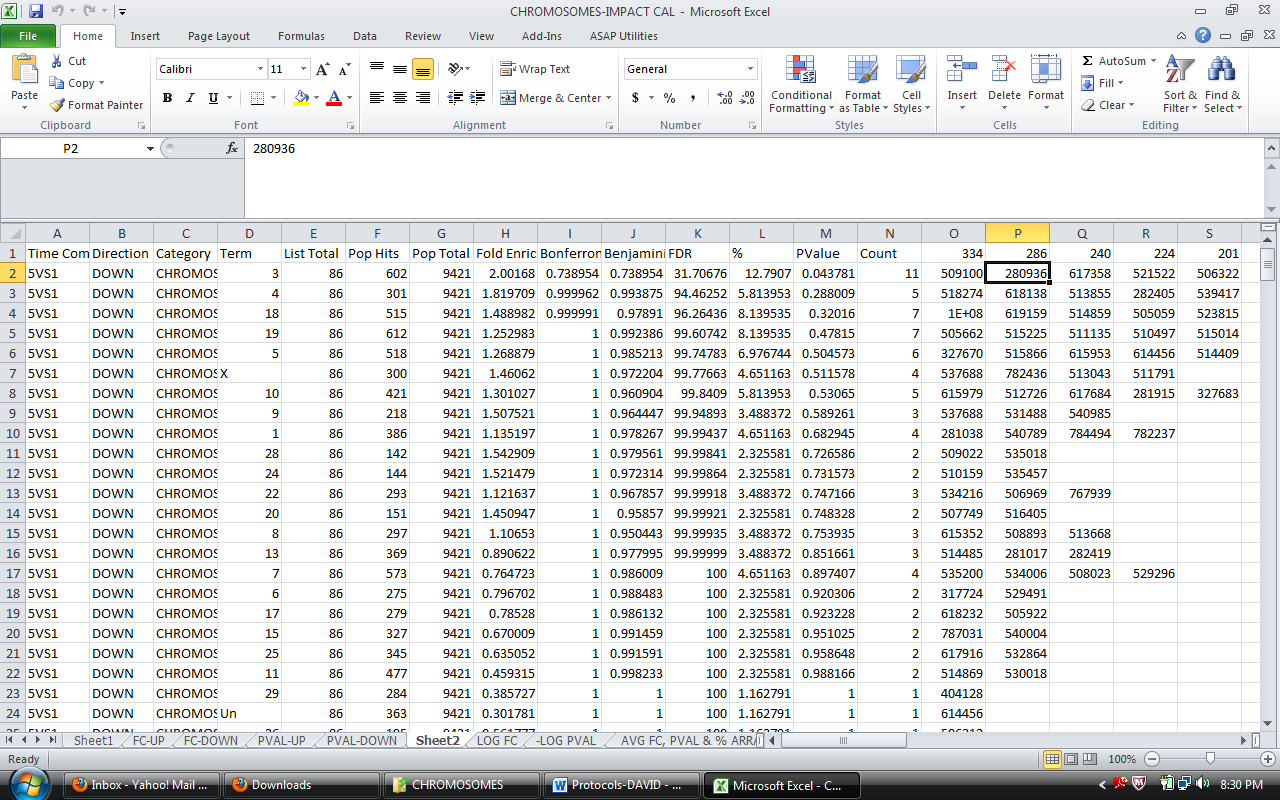


1. ***Preparation of input file***. The input file with all the microarray dataset plus the FDR value for the overall effect and the P-value between comparisons is prepared as follows:
2. Sort the data based on FDR. Eliminate the genes that do not pass the selected FDR cut-off. This will give equal number of genes in all comparisons.
3. Sort the data for each comparison based on respective P-values. Use the P-value cut-off for each comparison, and filter the genes that do not pass the cut off. This will give different number of genes across different comparisons.
4. Sort the data based on fold change (or expression ratio, NOT log-transformed data). Separate the up-regulated and down-regulated genes within each comparison based on fold change values.
5. Prepare the file in 4 different sheets.
6. Gene ID and ***fold change*** of up regulated genes for each comparison (called FC-UP);
7. Gene ID and ***fold change*** of down regulated genes for each comparison (called FC DOWN);
8. Gene ID and ***P-value*** of up regulated genes in each comparison (called PVAL-UP);
9. Gene ID and P-value of down regulated genes in each comparison (called PVAL_DOWN).

The sheets should look as follows:

***Example: Sheet with FC-UP***


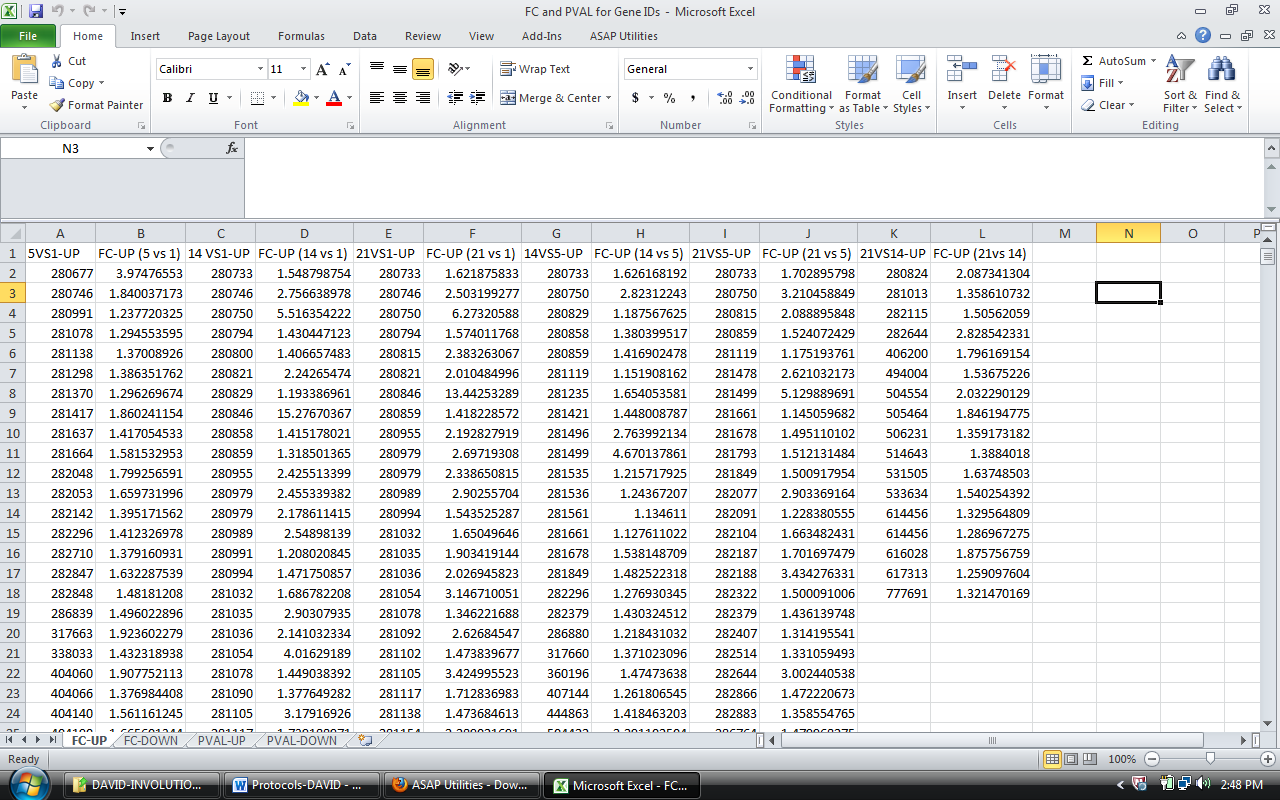


***Example: Sheet with PVAL-UP***


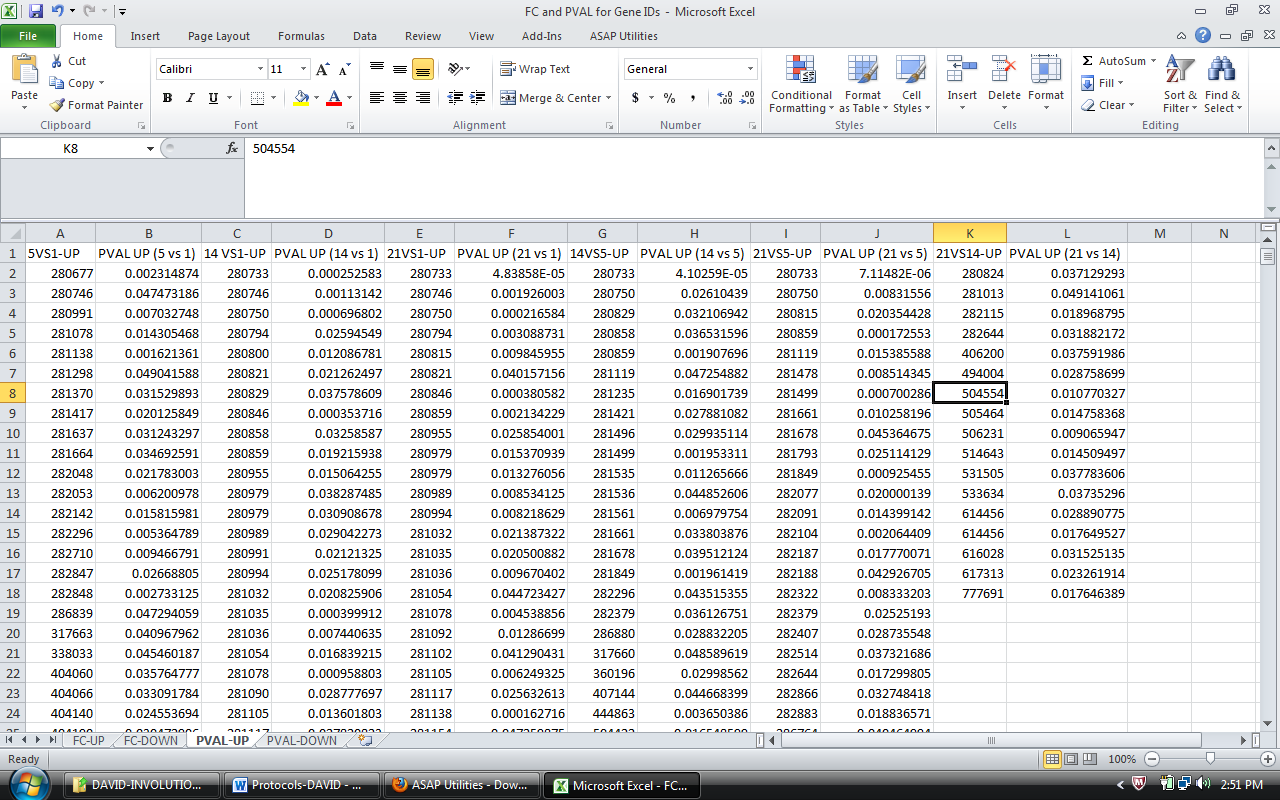


1. Insert a new sheet and name it LOGFC. All the columns from Sheet 2 are copied except those columns containing Gene IDs (i.e., columns after “count”)
2. Insert the formula to calculate the log-transformed values of fold changes (log_2_) in the second cell in the column after the “count” column (cell ‘O2’). This formula is extended to all the columns containing the gene IDs for each comparison and to all rows (the specific formula is not presented here for simplicity, a step-by-step protocol including the details of the formula is available upon request).
3. Similarly, in another sheet (named as ‘PVAL’) a formula is implemented for the calculation of the-log_10_ p-values of all the gene IDs in the sheet 2 starting from the cell ‘O2’.
4. Once the ‘LOGFC’ and ‘PVAL’ are ready, the calculation of average log2 FC, average –log10P value and % array is calculated in a new sheet. The % array is calculated with the number of genes in the term/’pop hit’ in DAVID (i.e., all the genes in the background or microarray).
5. After calculation of % array, -Log10 P-value and log2 fold change a “Pivot Table” is used to obtain a sheet for subsequent calculations. The Pivot Table is obtained as follows:

- Row label should have the term.
- The column labels should have the time comparison and direction of gene expression.
- Under each comparison for up regulation of genes, the calculated % array, average log2 fold change and average-log p-values should be inserted.
- For each term in the Pivot Table the “average” should be chosen in the “Value Field Setting”.

1. The data from the Pivot Table are copied and pasted into a new sheet and the **Impact** and **Flux** (or direction of the impact) calculated as follow:


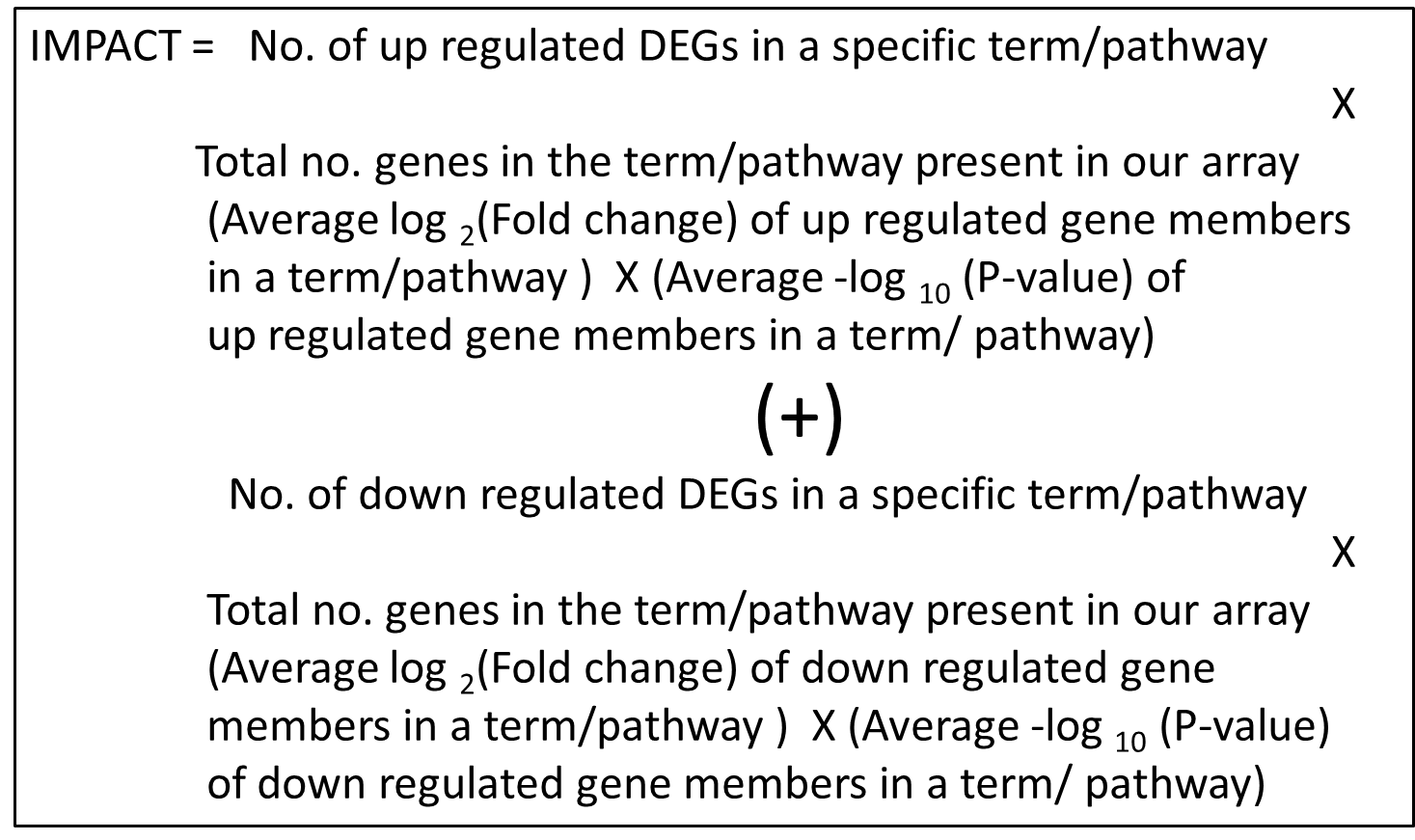

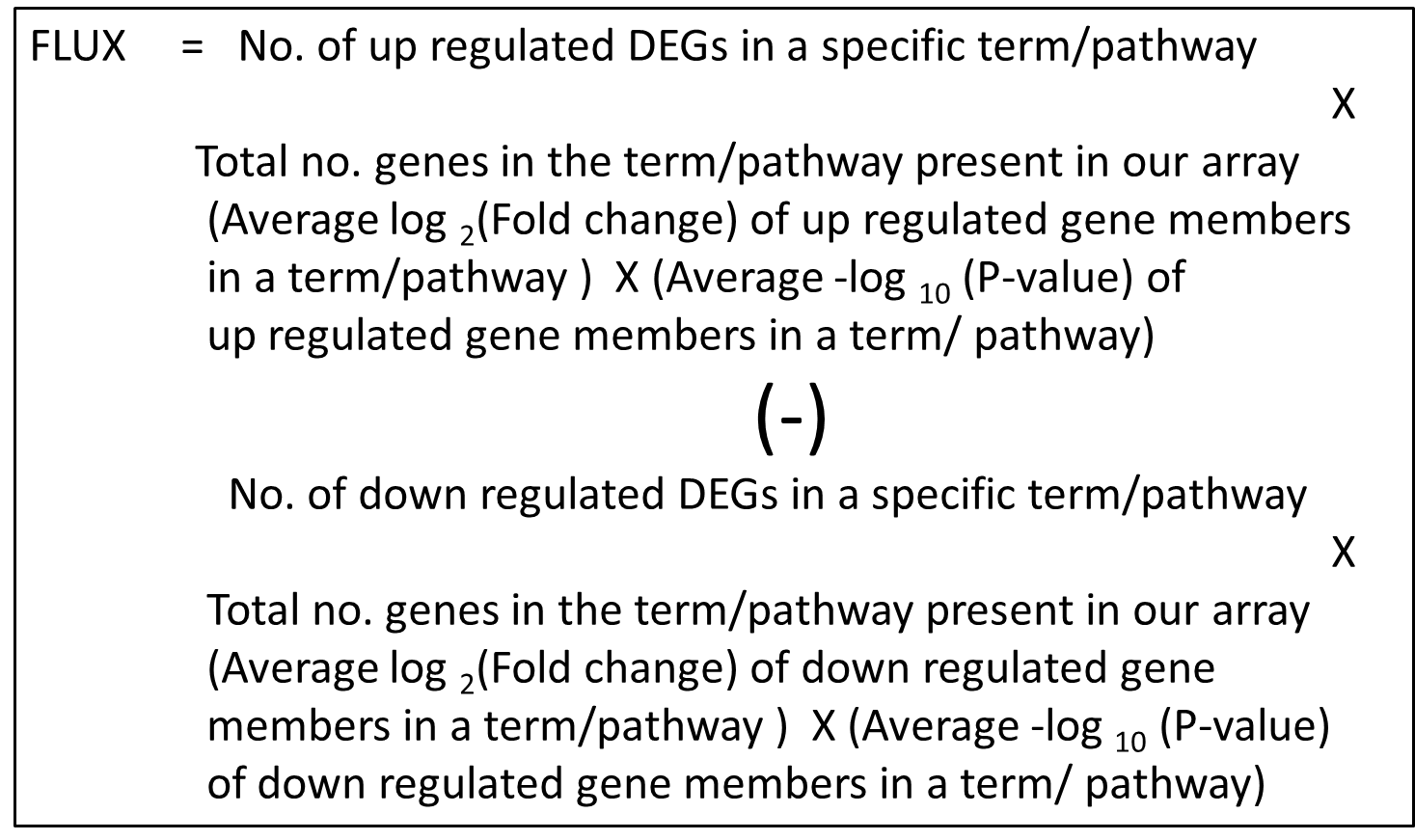


## Limitations and future implementation of DIA

We recognize that the DIA is not a flawless approach. Several limitations exist and implementation clearly could be performed in the future. Main limitations of the DIA are related to the approach itself but also are independent of it. Among the ones independent of the DIA, limitations are related to the sequence annotation (see above). There is lack of species-specific annotations (for most of the livestock species the gene/protein annotation is performed by orthology with the human or mouse annotation; however, a bovine annotation consortium has started to address such issue ), the annotation is still underway also for human and mouse (i.e., new functions for known proteins are continuously discovered) , and proteins can have cell/tissue specific functions which can differ between cell/tissue type (e.g., fatty acid binding proteins ); in the case of molecular biology, the reliance on transcriptome data to determine the biology of the cell is reductive considering that there is not a 1:1 ratio between mRNA and protein, the mRNA can have splice variants, its translation is strongly regulated (i.e., miRNA), the mRNA coded proteins can be subjected to differential post-translational modifications, and the proteins do not work in isolation but in a complex network of interactions. None of these factors are considered by the functional annotation tools available, including the DIA.

Additional limitations of the DIA (but also present in all the ORA approaches) are the fact that the system does not, and cannot, account for real flux, because it cannot account for the reagent amount (or amount of signaling molecules, in the specific case of signaling pathways) and all the genes products are considered to have the same effect on the pathways/functions.

The limitations related to the annotations will probably be solved in future studies. The limitations related to the DIA can be partly solved by future improvement of the system. The DIA can be easily associated with other systems able to provide network analysis. An additional variable that accounts for the relative weight of the gene product might help in adjusting for the importance of it in the pathway/functions, as previously implemented ; however, this addition might increase the error of the interpretation because it appears that the increase or decrease of any of the proteins in a pathway in isolation does not affect the flux (i.e., there are no “key” proteins per se) and all, or almost all, the proteins need to be modified in order to change the flux .

The DIA has been implemented in Microsoft Excel software with all the limitations related to memory usage. In the future the DIA will have to be implemented in other system more suitable to handle large amounts of data and providing faster calculations.

**References**

1. Loor JJ, Everts RE, Bionaz M, Dann HM, Morin DE, et al. (2007) Nutrition-induced ketosis alters metabolic and signaling gene networks in liver of periparturient dairy cows. Physiol Genomics 32: 105-116.

2. Bionaz M, Loor JJ (2008) Gene networks driving bovine milk fat synthesis during the lactation cycle. BMC Genomics 9: 366.

3. Bionaz M, Loor JJ (2011) Gene networks driving bovine mammary protein synthesis during the lactation cycle. Bioinform Biol Insights 5: 83-98.

4. Edgar R, Domrachev M, Lash AE (2002) Gene Expression Omnibus: NCBI gene expression and hybridization array data repository. Nucleic Acids Res 30: 207-210.

5. Bionaz M, Loor JJ (2007) Identification of reference genes for quantitative real-time PCR in the bovine mammary gland during the lactation cycle. Physiol Genomics 29: 312-319.

6. Wang M, Master SR, Chodosh LA (2006) Computational expression deconvolution in a complex mammalian organ. BMC Bioinformatics 7: 328.

7. Capuco AV, Wood DL, Baldwin R, McLeod K, Paape MJ (2001) Mammary cell number, proliferation, and apoptosis during a bovine lactation: relation to milk production and effect of bST. J Dairy Sci 84: 2177-2187.

8. Capuco AV, Akers RM, Smith JJ (1997) Mammary growth in Holstein cows during the dry period: quantification of nucleic acids and histology. J Dairy Sci 80: 477-487.

9. Seelig LL, Jr. (1980) Dynamics of leukocytes in rat mammary epithelium during pregnancy and lactation. Biol Reprod 22: 1211-1217.

10. Linzell JL (1974) Mammary Blood Flow and Substrate Uptake. In: Larson BL, Smith VR, editors. Lactation: a comprehensive treatise. New York: Academic Press. pp. 143-225.

11. Keenan TW, James Morré D, Huang CM (1974) Membranes and the Mammary Gland. In: Larson BL, Smith VR, editors. Lactation: a comprehensive treatise. New York: Academic Press. pp. 191-233.

12. Hollmann KH (1974) Cytology and Fine Structure of the Mammary Gland. In: Larson BL, Smith VR, editors. Lactation: a comprehensive treatise. New York: Academic Press. pp. 3-95.

13. Huang da W, Sherman BT, Lempicki RA (2009) Systematic and integrative analysis of large gene lists using DAVID bioinformatics resources. Nat Protoc 4: 44-57.

14. Piantoni P, Bionaz M, Graugnard DE, Daniels KM, Everts RE, et al. (2010) Functional and gene network analyses of transcriptional signatures characterizing pre-weaned bovine mammary parenchyma or fat pad uncovered novel inter-tissue signaling networks during development. BMC Genomics 11: 331.

15. Lemay DG, Neville MC, Rudolph MC, Pollard KS, German JB (2007) Gene regulatory networks in lactation: identification of global principles using bioinformatics. BMC Syst Biol 1: 56.

16. Gygi SP, Rochon Y, Franza BR, Aebersold R (1999) Correlation between protein and mRNA abundance in yeast. Mol Cell Biol 19: 1720-1730.

17. Schwanhausser B, Busse D, Li N, Dittmar G, Schuchhardt J, et al. (2011) Global quantification of mammalian gene expression control. Nature 473: 337-342.

18. Draghici S, Khatri P, Tarca AL, Amin K, Done A, et al. (2007) A systems biology approach for pathway level analysis. Genome Res 17: 1537-1545.

19. Reese JT, Childers CP, Sundaram JP, Dickens CM, Childs KL, et al. (2010) Bovine Genome Database: supporting community annotation and analysis of the Bos taurus genome. BMC Genomics 11: 645.

20. Kim IY, Shin JH, Seong JK (2010) Mouse phenogenomics, toolbox for functional annotation of human genome. BMB Rep 43: 79-90.

21. Storch J, Thumser AE (2010) Tissue-specific functions in the fatty acid-binding protein family. J Biol Chem 285: 32679-32683.

22. Morandini P (2009) Rethinking metabolic control. Plant Science 176: 441-451.
